# Commonality and variability in functional networks in children under 5 years old

**DOI:** 10.1038/s42003-026-10599-w

**Published:** 2026-07-07

**Authors:** Jiaxin Cindy Tu, Chenyan Lu, Trevor K. M. Day, Robert Hermosillo, Lucille A. Moore, Anxu Wang, Xintian Wang, Donna Dierker, Aidan Latham, Jeanette K. Kenley, Damien A. Fair, Jed T. Elison, Chad M. Sylvester, Barbara B. Warner, Joan L. Luby, Cynthia E. Rogers, Deanna M. Barch, Christopher D. Smyser, Timothy O. Laumann, Evan M. Gordon, Adam T. Eggebrecht, Muriah D. Wheelock

**Affiliations:** 1 Mallinckrodt Institute of Radiology, Washington University in St. Louis, St. Louis, MO USA; 2 Department of Psychological and Brain Sciences, Dartmouth College, Hanover, NH USA; 3 Masonic Institute for the Developing Brain, University of Minnesota, Minneapolis, MN USA; 4 Institute of Child Development, University of Minnesota, Minneapolis, MN USA; 5 Center for Brain Plasticity and Recovery, Georgetown University, Washington, DC USA; 6 Department of Neurology, Washington University in St. Louis, St. Louis, MO USA; 7 Department of Psychiatry, Washington University in St. Louis, St. Louis, MO USA; 8 The Taylor Family Institute for Innovative Psychiatric Research, Washington University in St. Louis, St. Louis, MO USA; 9 Department of Pediatrics, Washington University in St. Louis, St. Louis, MO USA; 10 Department of Psychological and Brain Sciences, Washington University in St. Louis, St. Louis, MO USA

**Keywords:** Development of the nervous system, Neuroscience, Network models

## Abstract

Functional brain networks support human cognition, yet how individualized network architecture emerges in early childhood remains poorly understood. Averaging across participants can obscure age-specific organization and person-to-person differences, particularly in slowly developing association cortices. We developed an age-appropriate functional reference that captured common structure across toddlers without averaging away individual variability, enabling estimation of each child’s networks from resting-state fMRI. Across cohorts of 8-60-month-old children, we found individualized network organization-including finer-scale subdivisions and emerging language lateralization well before age five. Network layouts showed longitudinal stability, with greater consistency in sensory than association regions. Within-network connectivity was stronger and explained age-related variance when networks were defined using individualized rather than group-consensus topography. Left-lateralization of language networks tracked age-normalized verbal ability, linking early functional architecture to emerging cognition. These findings show that behaviorally relevant brain networks arise far earlier than previously recognized, providing a foundation for studying typical development and early biomarkers.

## Abstract (web summary)

Functional brain networks in toddlers show individualized organization, emerging language lateralization, and behaviorally relevant architecture years earlier than previously recognized.

## Introduction

Functional network patterns in young children (<5 years) have been characterized at the group level^1–6^. However, these group-average pediatric brain networks may not comprehensively represent the organization of the developing brain. Recent studies in adults have demonstrated idiosyncratic yet reliable features in functional network topography across individuals that are qualitatively different from group-average estimates^7–13^. These trait-like topographical features are stable across both sessions^14^ and task states^15^, and may be related to individual behavioral characteristics^16^ or clinical phenotypes^17^. Group-averaging procedures not only fail to capture these individual-specific features, but they also tend to obscure networks with highly variable spatial topographies across individuals^18^. Therefore, we reasoned that using an approach that circumvents group averaging could potentially reveal finer network details in a pediatric population, which may reveal unique developmental changes and variability.

Reliable identification of individual-specific functional networks with unsupervised clustering/community detection techniques often requires extended data acquisition. For example, with the commonly used Infomap algorithm^19,20^, more than 90–100 min of data was recommended^10,13^. However, starting these analyses de novo for every new individual might not be necessary, given that most resting-state functional network topography is consistent across individuals^9,12,21^. Consequently, several prior-based methods have been developed to derive individual-specific functional networks in populations with limited data available per individual^22–28^, significantly lowering the data requirement^24,25^.

Although the aforementioned prior-based methods have significantly advanced the mapping of individual-specific network organizations, the selection of network priors remains a critical decision that has been frequently overlooked, especially when applying these methods to populations beyond healthy, young adults. For example, previous work has used a set of adult network priors for data acquired in children younger than 5 years old^29,30^, which assumes that the number and spatial topography of functional networks in pediatric cohorts are reasonably similar to the adult population average. However, mounting evidence suggests functional connectivity (FC) and network features in young children consistently differ from older children and adults^1–6,31–35^. Notably, while some studies have proposed deriving infant-specific network templates by averaging BOLD time series at cortical locations defined by adult networks to calculate network-average FC profiles^29^, it remains unclear whether this approach necessarily yields truly “age-specific” networks. To date, few studies have delineated individual-specific networks in children younger than 5 years old using age-specific group priors^36^.

Hemispheric lateralization of functional networks has been consistently observed in prior literature, especially in heteromodal association cortices^28,37,38^. The language network exhibits a left-hemisphere bias^28,39,40^ that strengthens from childhood into young adulthood^41–46^. However, it remains unclear how early hemispheric lateralization emerges and whether it relates to the development of behavioral functions, such as language. The language network is therefore a particularly well-suited model system for evaluating individualized network mapping in early childhood: it has a well-characterized lateralization profile in adults, a protracted developmental trajectory, and a direct link to verbal ability, making it a sensitive testbed for detecting whether individualized network organization captures meaningful neurodevelopmental variation.

Here, we investigate functional network organization and its variability in children aged 1–5 years across two pediatric datasets, using age-specific group-level network templates as priors to estimate vertex-level individual-specific networks. We find that individualized network organization, including finer-scale subdivisions not visible in group averages, is present well before age five, with network topographies showing longitudinal stability from age two to three. Individualized topography outperformed group-consensus definitions in within-network FC and explained substantially more age-related variance, with language network left-lateralization increasing across the first five years of life and specifically associating with age-normalized verbal ability but not non-verbal ability. These findings demonstrate that behaviorally relevant individualized brain networks emerge far earlier than previously recognized, providing a foundation for studying typical neurodevelopment and identifying early functional biomarkers.

## Results

### Identification of age-appropriate functional connectivity patterns using concatenated individual data

**Fig. 1:**
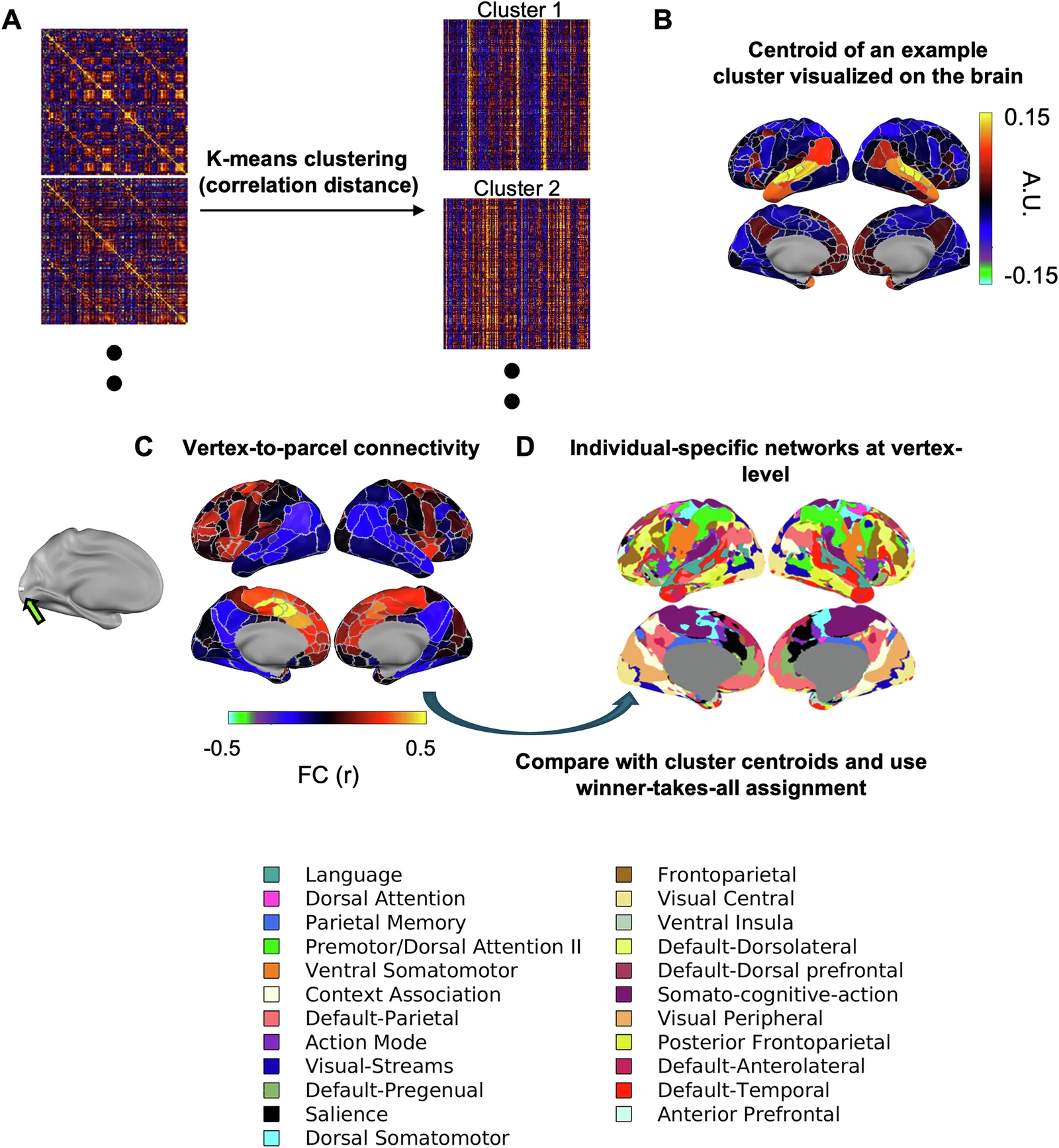
Creation of group-level reference template and individual-specific network mapping. **A** Example FC matrices being concatenated and clustered into internally similar clusters of FC profiles. **B** Example template FC profiles (centroids) for the Language network. **C** Vertex-to-parcel connectivity. **D** Individual-specific networks at vertex-level. Network names and colors were chosen with reference to prior literature.

Previously, we identified functional networks based on 2-year-old children using group-averaged FC computed within an age-specific area parcellation^5^. While this approach captures dominant population-level organization, anatomically matched cortical areas can exhibit distinct FC profiles across individuals during early development. As a result, averaging FC across participants may obscure both inter-individual variability and connectivity patterns that are consistently expressed only in subsets of children (Supplementary Fig. 1**;** Supplementary Note 1). To mitigate this limitation, we identified age-appropriate FC patterns by clustering concatenated individual FC profiles, rather than group-averaged connectivity (Fig. 1A). Using the same area parcellation as in prior work^5^, FC was computed between all pairs of parcels within each session, yielding parcel-wise FC profiles where each parcel served both as a feature (connectivity target) and as a sample, pooled across individuals. Clustering these parcel-wise FC profiles across participants allows common connectivity patterns to emerge without requiring them to be present in every individual. We clustered FC profiles from 81 fMRI sessions (72 unique children aged 2–3 years, where 9 out of 72 children had longitudinal data at Y2 and Y3; all sessions >20 minutes of low-motion data; eLABE Dataset 1; demographics in Supplementary Fig. 2), analogous to prior literature^47,48^. A split-half stability analysis revealed a local peak at 23 clusters (Supplementary Fig. 3). Full methodological details are provided in the Methods section.

### Individual-specific network mapping

The resulting 23 resting-state network profiles represent common FC patterns observed across subjects at 2–3 years. The cluster centroids, therefore, serve as age-appropriate reference FC profiles or “network templates” (example in Fig. 1B; all 23 networks in Supplementary Fig. 4). These reference profiles were subsequently used to delineate individual-specific network organization at finer spatial resolution. Specifically, vertex-level FC profiles (vertex-to-parcel connectivity maps, Fig. 1C) were compared with the 23 reference profiles using Pearson’s correlation, and each vertex was assigned to the most similar network using a winner-take-all procedure, following prior work^22^. This step enables detailed vertex-wise network mapping (Fig. 1D) while maintaining correspondence with the age-appropriate connectivity patterns identified above. For completeness, we repeated the template-matching procedure using network templates derived from the 19-network group-average FC solution from our prior work in this age range^5^. Although the resulting network topographies were broadly similar, the data-driven templates more finely differentiated certain regions and yielded fewer vertices with low template correlations (Supplementary Fig. 5–7**;** Supplementary Note 2).

### The 23 networks match the fine network divisions in adults and refined our understanding of functional networks in young children

**Fig. 2:**
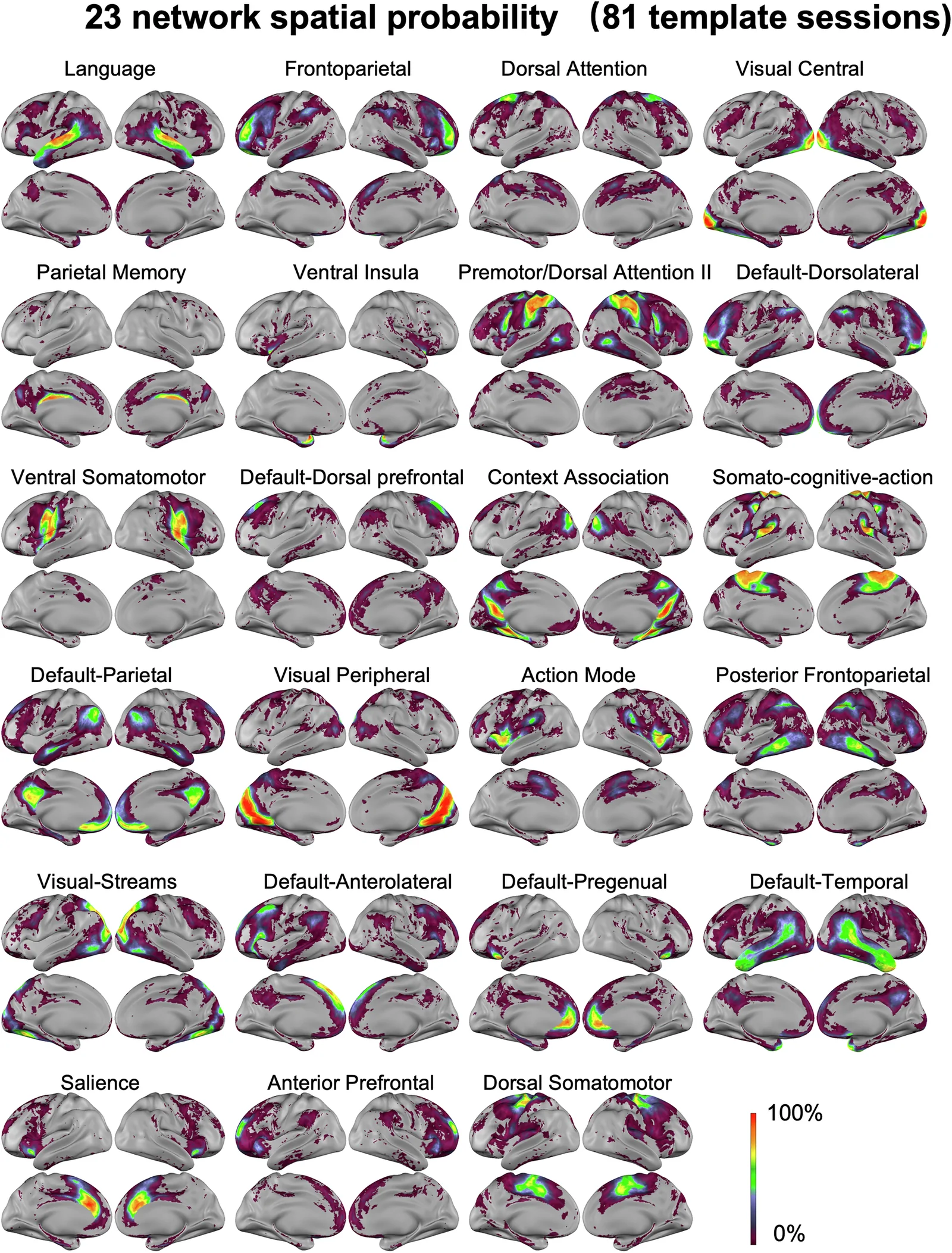
Functional networks spatial probability. For each vertex, the spatial probability of the network is quantified as the percentage of sessions within the 81 template sessions with that matching network assignment.

Network spatial probability plots^9,17,24,40^ show the most and least common locations of a network across the 81 sessions (Fig. 2). To help put the networks into context and relate them to potential behavioral significance, we further applied the Network Correspondence Toolbox^49^ and “NiMARE: Neuroimaging Meta-Analysis Research Environment” version 0.5.0^50^ to compare the topography of our 23 networks to prior literature (Supplementary Figs. 8–19, Supplementary Note 3). Because task-based fMRI or multimodal data were not available in the present study, functional interpretations of the identified networks are necessarily indirect and based on spatial correspondence with prior reports rather than direct functional validation. Despite the age difference between young children and adults, some of the 23 functional networks have a spatial topography that overlaps with previously identified fine network divisions in adult studies^17,25,51,52^. Additionally, the location of less common network occurrences was also consistent with results using highly sampled resting-state fMRI data. For example, we found that the salience network occasionally extends to regions normally assigned to the parietal memory network in some individuals, consistent with a prior report in adults^53^. The interdigitation of the Salience (black) and Default-Pregenual (pistachio green) networks at the frontal operculum and the medial prefrontal cortex also resembled the interdigitation of the Salience and Default Mode networks in resting-state fMRI from highly-sampled adult individuals^8,10,17^.

To further compare and group the 23 fine network divisions identified here with prior reports in adults, we quantified the similarity between each network to the 20 finely divided adult networks from 45 highly sampled adult individuals^17^ based on both network spatial probability maps (Fig. 2) as well as the average network FC (Supplementary Fig. 20) to calculate a weighted similarity score (Supplementary Fig. 21) as described in this paper^17^. The average network FC for a given network was obtained by averaging FC across all vertices assigned to that network in each subject, then averaging across subjects. These 20 finely divided adult networks were selected because Lynch and colleagues^17^ derived their spatial probability maps and average FC from high-density precision fMRI data, enabling finer network parcellation than other group-based approaches, and allowing us to assess whether our divisions reflect established adult networks or children-specific ones. Although most of the 23 networks had clear correspondence with the 20 adult networks, some of them (e.g., “Anterior Prefrontal”, “Default-Dorsal prefrontal” and “Default-Temporal”) did not, potentially suggesting some networks that are present in childhood are later incorporated into other networks in adulthood (Supplementary Fig. 22). Additionally, there is a much smaller Action Mode Network in young children compared to adults, especially around the dorsal anterior cingulate cortex. Parts of the ventral insula and dorsal somatomotor network in young children might later be incorporated into the Action Mode Network (Supplementary Fig. 22). Furthermore, while the frontoparietal network was divided into more cohesive pieces in other studies^47,51^, there was less anterior-posterior bias in the divisions than those reported here. A summary of putative counterparts of our networks in existing literature can be found at Supplementary Table 1.

### Individual-specific functional networks in young children are stable within sessions and across longitudinal sessions one year apart

**Fig. 3:**
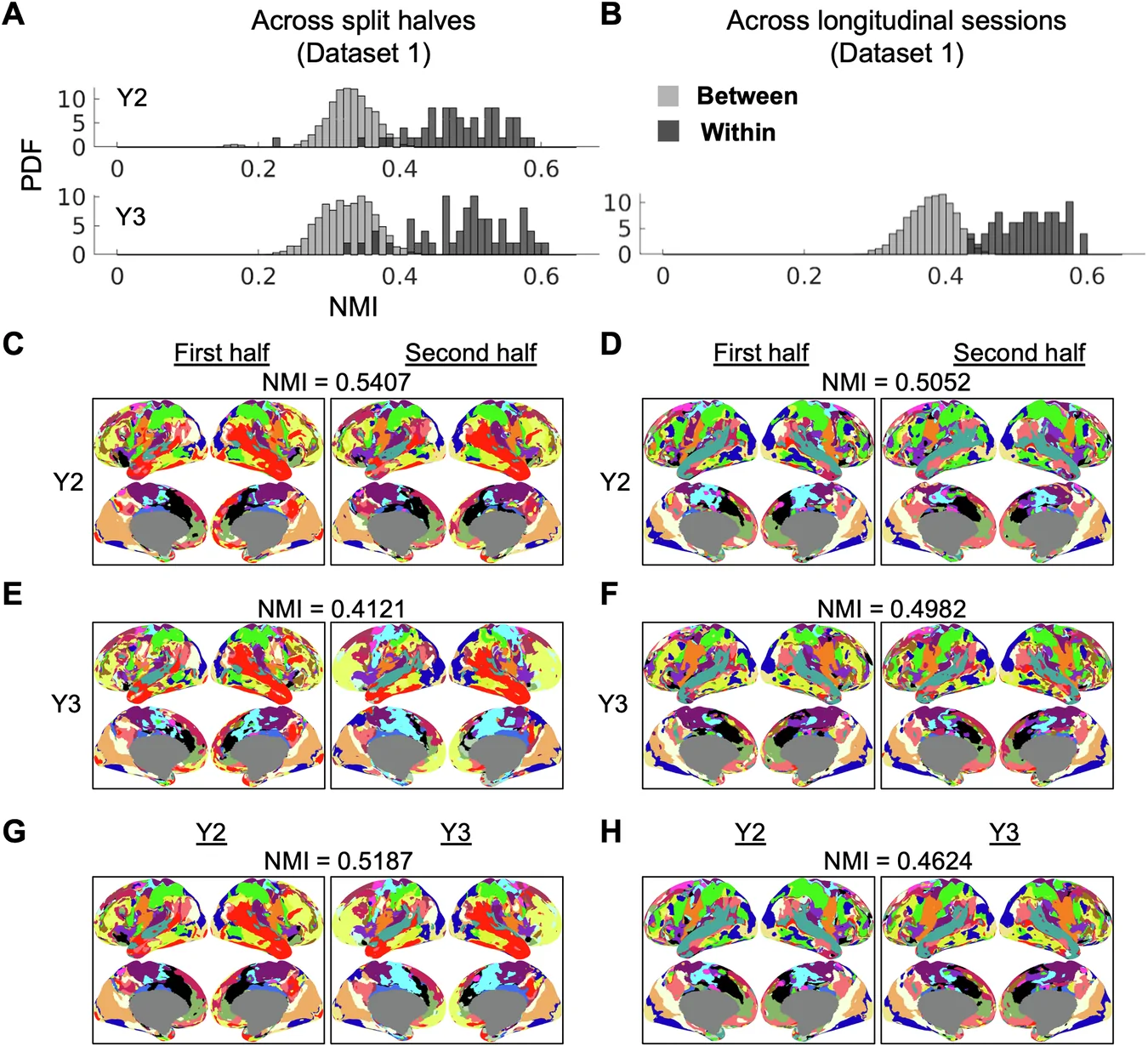
Individual-specific network topography for 23 networks (Dataset 1, *N* = 49 participants each contributing one session at Y2 and one session at Y3). **A** Within- versus between-individual NMI computed across split halves at each time point separately. PDF probability density function. **B** Within- versus between-individual NMI computed across the two longitudinal sessions. **C**, **D** Network assignments for example individuals at Y2. **E**, **F** Same as (**C–E**) but at Y3. **G**, **H** Network assignments for example individuals across the Y2 and Y3 time points.

To examine the individual-specificity of these functional networks, we obtained the individual functional network assignments using the subset of 49 participants in Dataset 1 with two longitudinal sessions at Y2 and Y3 time points (2.08 ± 0.12 and 3.16 ± 0.24 years old, respectively) with the same procedure described above (Fig. 1). First, we derived functional networks from the first half and second half of each fMRI session. The consistency of network assignment across the split halves (measured with normalized mutual information, NMI) was significantly higher (two-sample t-test *p* < 0.001) within subject (Y2: NMI = 0.4822 ± 0.0669, Y3: NMI = 0.4919 ± 0.0686) than between subjects (Y2: NMI = 0.3274 ± 0.0383, Y3: NMI = 0.3250 ± 0.0379) (Fig. 3a, C–F), with NMI higher within subject in almost all subjects (Y2: 49, Y3: 47 out of 49), suggesting that the network assignment contain individual-unique features. As a sanity check, the NMI between the network assignments in the first half and the randomly permuted network assignments in the second half is much lower, at approximately 0.001: for Fig. 3C, the 95% confidence interval of the permutation null is [0.0013, 0.0016]. To address the potential inflation of NMI by including frames from the same run in the two split halves, we repeated the analysis without overlapping runs, and the within-individual NMI was still significantly higher (Supplementary Fig. 23**;** Supplementary Note 4). To ensure the robustness of our results, we conducted the split-half network reliability analysis in an additional dataset of 8-60 months’ children from the Baby Connectome Project (Dataset 2, demographics described in Supplementary Fig. 24). Qualitatively similar results with higher within-subject NMI were obtained using N = 178 participants from Dataset 2 using their longitudinal session with the most low-motion data (Supplementary Fig. 25A-C). However, within-subject NMI was positively correlated with the length (in minutes) of low-motion data in each split-half (*r* = 0.6447, *p* < 0.001), with the total length (in minutes) of scan time (*r* = 0.5781, *p* < 0.001), and was negatively correlated with the mean framewise displacement of the scan session (*r* = -0.1336, *p* = 0.0204) but was not significantly correlated with age (*r* = -0.0553, *p* = 0.3390; Supplementary Fig. 25D). This suggests that the ability to retrieve reliable networks was somewhat limited by the data amount and quality.

To assess the longitudinal stability of the individual-specific networks, we also derived individual-specific functional networks from the whole fMRI session for the same 49 subjects but across Y2 and Y3 time points (Dataset 1). Because of the relationship between NMI and length of data reported above, we opted to use the whole session (Y2: 19.0 ± 4.1 min low-motion data, Y3: 18.7 ± 3.7 min low-motion data) for this analysis, unlike the prior split-half analysis, which used about half of the data amount to generate the networks. The consistency of network assignment across the two longitudinal sessions was significantly higher (two-sample t-test *p* < 0.001) within subject (NMI = 0.5190 ± 0.0444) than between subjects (NMI = 0.3788 ± 0.0330) (Fig. 3B, G, H), and the NMI was higher within subject for all 49 subjects, indicating the individual-specific networks were stable from 2 to 3 years old. Although the within-subject NMI values may seem low, they are in line with those reported using a similar template-matching approach in the MSC dataset (a high-quality adult dataset with ~5 h of resting-state fMRI). In that study^24^, within-subject NMI ranged from 0.527 to 0.648 using all available low-motion data after frame censoring, and was around 0.5 using only 15 min of contiguous low-motion data. To further explore the amount of data required to achieve reliability and individual-specificity of the networks, we conducted additional analysis with different lengths of contiguous low-motion data (minimum 2.5 min and maximum of 20 min in steps of 2.5 min; Supplementary Fig. 26**;** Supplementary Note 5). We found that the within-subject NMI was higher than between-subject NMI with as little as 2.5 min of low-motion data *t*(2399) = 14.30, *p* < 0.0001 (Supplementary Table 2), and the two diverged by 7.5 minutes based on confidence interval (Supplementary Fig. 26E**;** Supplementary Note 5). Taken together, we found that relatively stable individual-specific functional networks can be observed within a scan session and also across longitudinal sessions in children under 5 years old.

### Population consensus of individual-specific network assignments demonstrated high variability in the lateral frontal cortex, temporal-parietal junction, and the occipital-temporal cortex

**Fig. 4:**
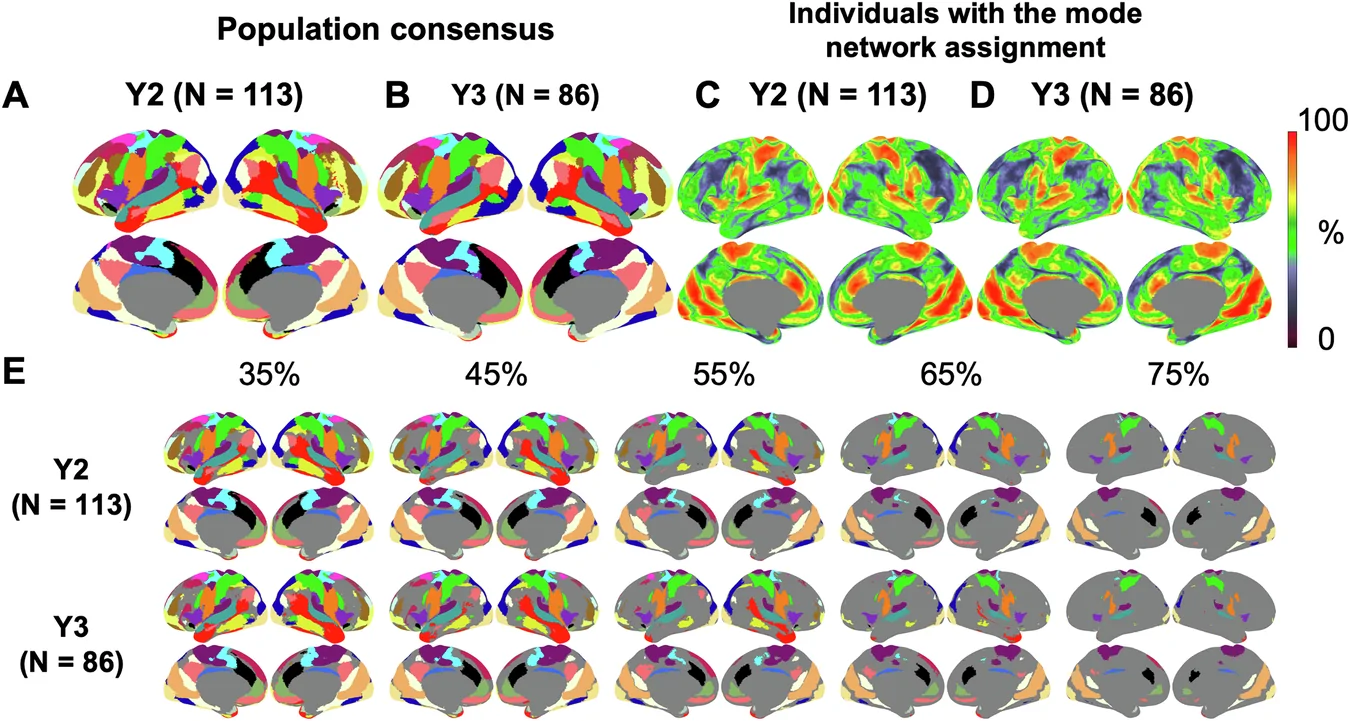
Population consensus network patterns (most frequent assignment) across subjects (Dataset 1). **A** Population consensus (Y2, *N* = 113 participants). **B** Population consensus (Y3, *N* = 86 participants). **C**, **D** Percentage of subjects with the same assignment as the most frequent assignment. **E** Population consensus of network identity for only the locations with consistent assignment among at least 35, 45, 55, 65, and 75% subjects (*N* = 113 at Y2, *N* = 86 at Y3).

Population consensus (i.e., the most frequent network at each vertex across the population) across all available subjects at Y2 (*N* = 113 subjects, Fig. 4A) and at Y3 (*N* = 86 subjects, Fig. 4B) of Dataset 1 appears to be similar to each other. Consistent with previous results from adults^11,12,25,26,54^, we found low variability at unimodal processing regions, such as the auditory, visual, and somatosensory and somatomotor cortices, and high variability at the lateral frontal cortex, temporal-parietal junction (TPJ), and the occipital-temporal cortex based on the percentage of subjects with the most frequent assignment (Fig. 4C, D). Since this percentage only considered the most frequent assignment but not the frequency of other assignments, we also calculated a complementary “versatility”^55^ measure (Supplementary Fig. 27), which captures how broadly a vertex distributes its assignments across networks rather than just whether it switches. Results from this measure did not change our conclusions. To facilitate comparison to the spatial distribution of the most consistent network assignment reported in older children at 9–10 years^24^, we also applied a threshold of at least 35, 45, 55, 65, and 75% of individuals on the population consensus network assignments in Dataset 1 (Fig. 4E). The locations with the least agreement among participants aligned with the boundaries between network patches, consistent with previous findings in adults where differences in borders among individual participants contributed to the majority of network variability^11,56^.

### Within-network connectivity is higher and reflects more age-related variance for individualized network topography compared to the group consensus network topography

**Fig. 5:**
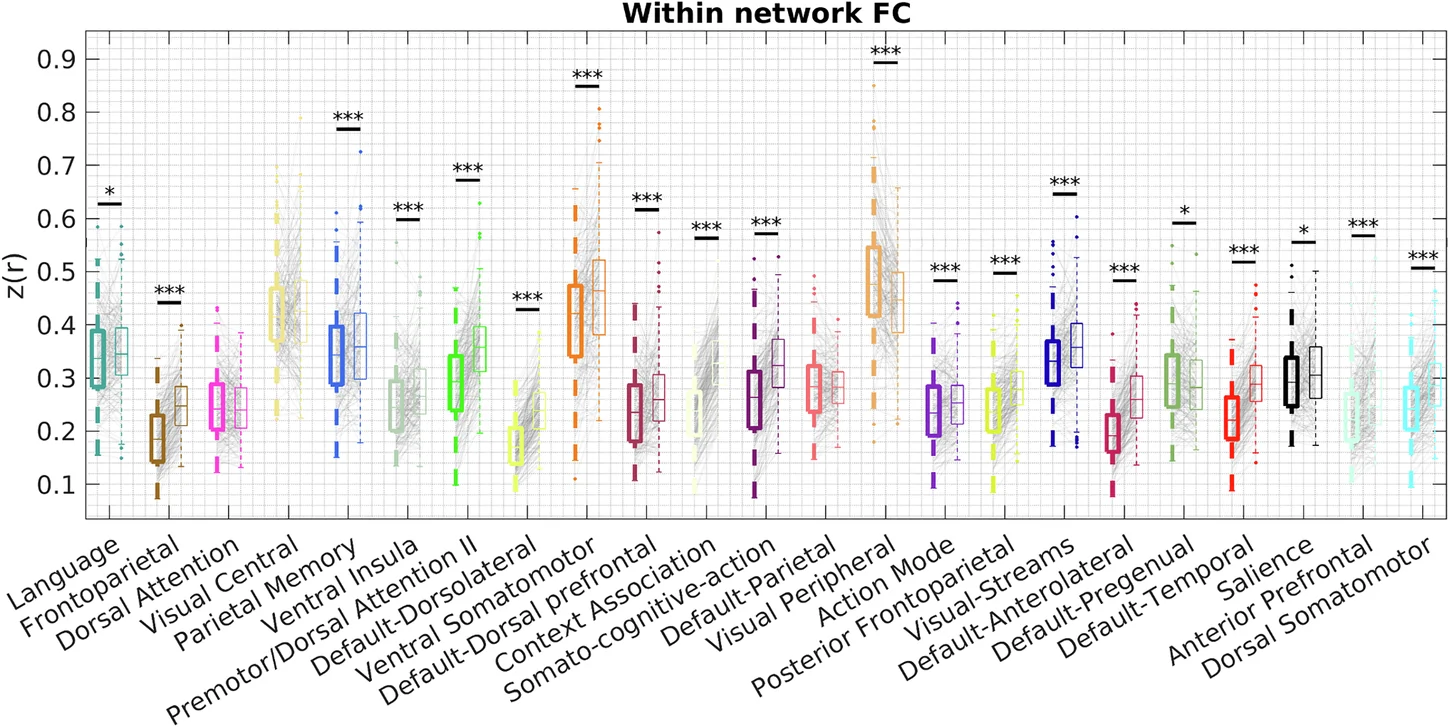
Within-network FC using network topography defined by group consensus or individualized network (Dataset 1, *N* = 199 independent sessions). **A** Within-network FC with network topography defined by group consensus (bolded, left) or individualized network (right). * *p* < 0.05, ** *p* < 0.01, *** *p* < 0.001. FDR-corrected.

So far, we have demonstrated the individual-specificity of the individualized network and the locations of variability. It remains unclear whether the individualized networks were superior to the group-level networks in capturing truly meaningful, biologically relevant signals in individuals. If a network is correctly delineated, the regions assigned to it should be more strongly connected to one another. We compared within-network FC for networks defined by individualized versus group consensus topography across all available sessions (Y2 and Y3 combined, *N* = 199). The group consensus was derived from the full dataset rather than age-specific subsets to avoid confounding the comparison with age-related differences in the consensus map itself, even though practically it made little difference. We found that within-network FC was significantly higher for the individualized network topography compared with the group consensus network topography for 18 out of 23 networks (FDR-corrected *p* < 0.05, paired t-test, Fig. 5). Dorsal Attention, Visual Central and Default-Parietal networks did not demonstrate a significant difference, while Visual Peripheral and Default-Pregenual had a significantly lower within-network FC for individualized network topography compared with the group consensus network topography (FDR-corrected *p* < 0.05). The Visual Peripheral network result is potentially due to the group consensus network capturing the most strongly connected region, which was highly consistent across individuals, whereas individualized networks sometimes exhibit weaker FC at the boundaries.

To assess whether within-network FC captures developmental information beyond motion, we compared models using mean framewise displacement (FD) alone to models that additionally included within-network FC features. Mean FD alone explained negligible variance in age (R² ≈ 0). Adding FC from individualized network topographies explained substantially more variance than adding FC from group-consensus topographies (ΔR² = 0.35 vs. 0.21; adjusted R² = 0.26 vs. 0.11), despite identical model complexity. To evaluate generalization at the subject level, we ran 5-fold cross-validation grouped by subject using ridge regression, with mean FD and within-network FC from all 23 networks as predictors. Sessions from the same subject were kept within the same fold, and prediction accuracy was assessed by correlating out-of-sample predicted and true ages. Prediction accuracy was evaluated by aggregating out-of-sample predictions across folds and computing the correlation between predicted and true age. Models using individualized topographies again outperformed those using group-consensus topographies (r = 0.456, R² = 0.208 vs. r = 0.288, R² = 0.083), confirming that individualized network representations capture developmentally relevant information that group-level definitions miss.

### Developmental variability in hemispheric lateralization is reflected in the individual-specific functional network topography

Building on prior literature documenting network lateralization, particularly in language systems, we tested two hypotheses: that language-related networks become increasingly lateralized across early childhood, and that this lateralization relates to individual language ability. We focused on two networks encompassing regions consistently implicated in language function^39,40,48^: Network 1 (“Language”) and Network 18 (“default-anterolateral”).

**Fig. 6:**
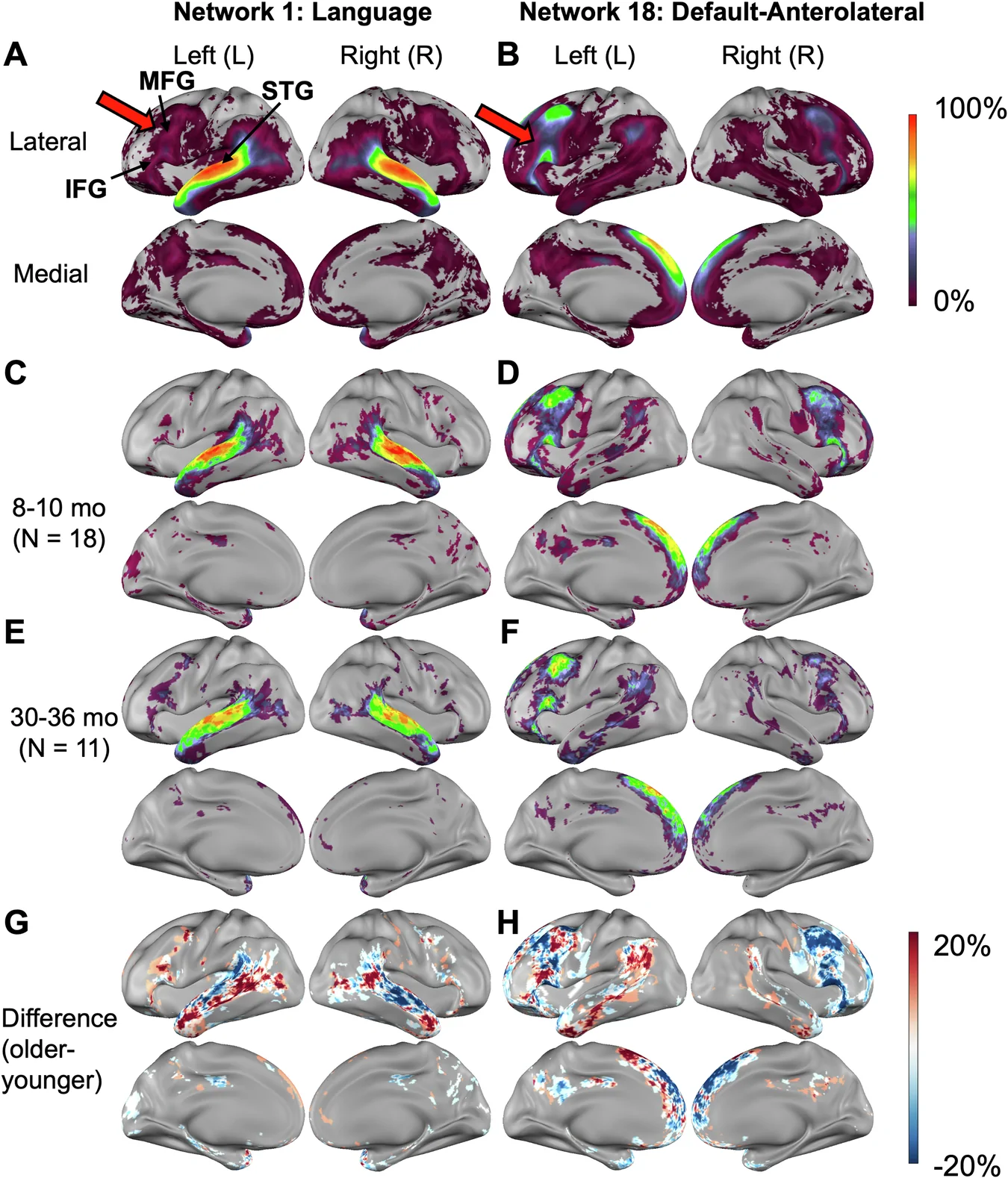
Spatial probability of Network 1 (“Language”) and Network 18 (“Default-Anterolateral”) across age groups (Dataset 2). **A**, **B** 8–60 months (*N* = 301 sessions from 178 participants). **C**, **D** 8–10 months (*N* = 18 participants). **E**, **F** 30–36 months (*N* = 11 participants). **G**, **H** Difference between the older and younger groups. MFG Middle Frontal Gyrus, IFG Inferior Frontal Gyrus, STG Superior Temporal Gyrus.

**Fig. 7:**
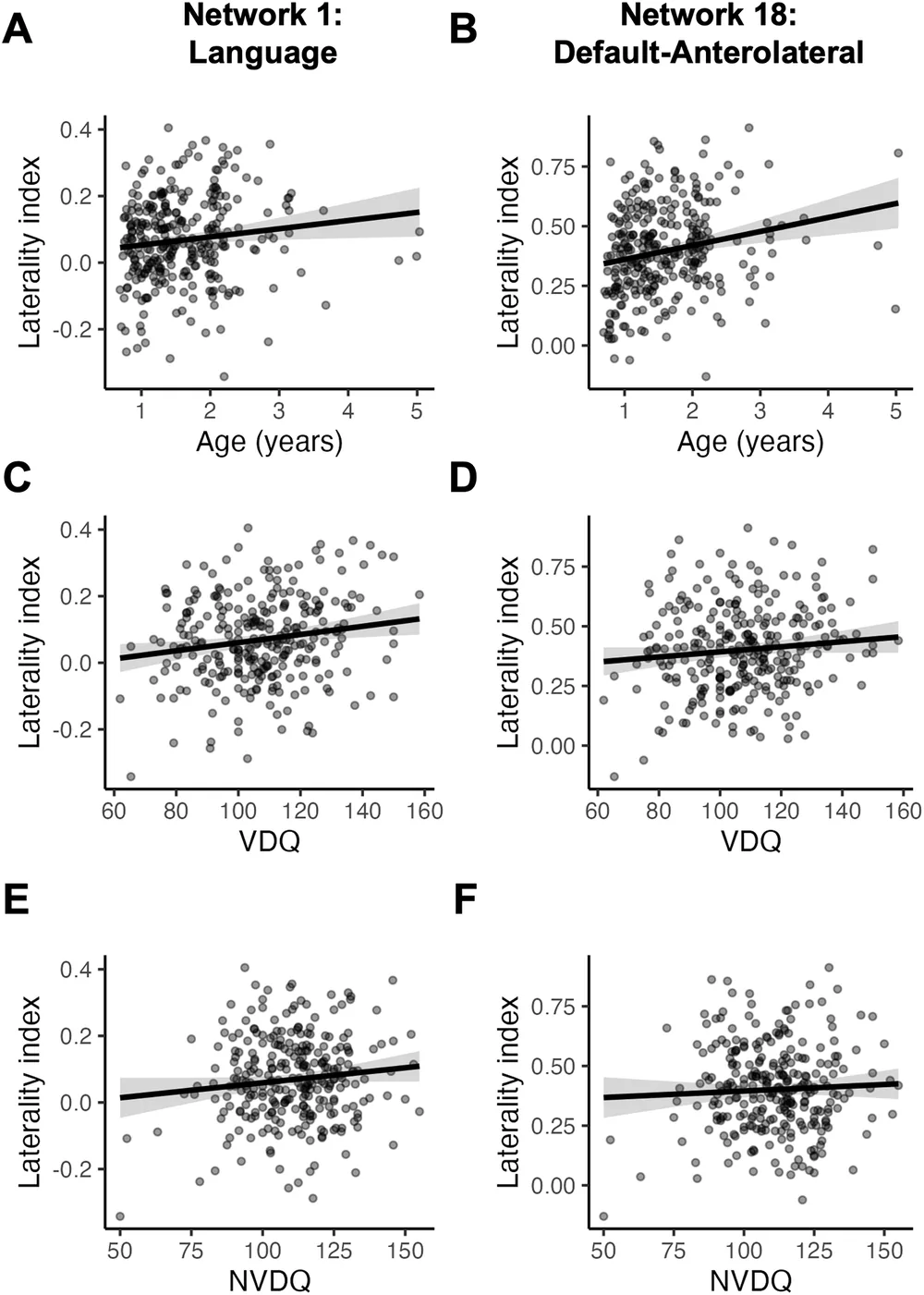
The relationship between functional network laterality and age or Mullen Scales of Early Learning developmental quotients (Dataset 2, *N* = 178 participants contributing 301 sessions total). Laterality index against **A**, **B**) age (*N* = 301 sessions), **C**, **D** verbal developmental quotient (VDQ, *N* = 280 sessions), **E**, **F** non-verbal developmental quotient (NVDQ, *N* = 282 sessions). Shaded band = 95% CI of fixed effect (age was held constant in (**C–F**)).

We first examined Dataset 2, which offers a larger cross-sectional sample spanning a relatively broad developmental window. Across children scanned between 8 and 60 months, the Network 1 was primarily anchored in the superior temporal gyrus (STG), a region central to auditory language processing. In some children, it also incorporated the inferior frontal gyrus (IFG) and middle frontal gyrus (MFG), key components of the adult language network^39,40^ that additionally contribute to domain-general processing^57,58^ (Fig. 6A, red arrows), although these regions were most commonly assigned to Network 18 (Fig. 6B, red arrows). Relative to children aged 8–10 months, those aged 30–36 months showed greater left-hemisphere involvement and reduced right-hemisphere involvement across both networks, reflecting an increase in lateralization over this developmental period (Fig. 6C–H). To quantify this network lateralization, we calculated a “laterality index” (LI) defined as the difference in the network surface area between the left and right hemispheres, expressed as a percentage of the total network surface area. We found that Network 1 showed weak left-lateralization (LI = 0.07 ± 0.13; one-sample t-test, *t*(300) = 9.2858, *p* < 0.001), whereas the Network 18 showed strong left-lateralization (LI = 0.40 ± 0.19; *t*(300) = 37.1876, *p* < 0.001). Using linear mixed-effects models with a random intercept for subject, laterality of Network 1 increased with age (*β* = 0.025 per year, *SE* = 0.011, *t* = 2.29, *df* = 299.0, *p* = 0.023, *standardized β* = 0.131, Fig. 7A). Laterality of Network 18 also increased with age (*β* = 0.059 per year, *SE* = 0.015, *t* = 3.82, *df* = 299.0, *p* < 0.001, *standardized β* = 0.216, Fig. 7B). These associations were robust to the addition of mean FD of the scan session.

Associations between network laterality and behavioral measures were then assessed using linear mixed-effects models, including age and behavioral scores as fixed effects and a random intercept for subject. Verbal and non-verbal abilities were quantified using the Mullen Verbal Developmental Quotient and Non-verbal Developmental Quotient (VDQ and NVDQ; age-normalized scores from the Mullen Scales of Early Learning^59^), excluding sessions without available scores (21 without VDQ and 19 without NVDQ). Network 1 laterality was positively associated with verbal ability beyond the effect of age (*β* = 0.00122 per VDQ point, SE = 0.00044, t = 2.79, df = 271.6, *p* = 0.0057, standardized *β* = 0.169, Fig. 7C). Network 18 showed a similar trend, though this did not reach statistical significance (β = 0.00107 per VDQ point, SE = 0.00062, t = 1.72, df = 269.1, *p* = 0.087, standardized *β* = 0.102, Fig. 7D). To assess specificity to verbal ability, neither Network 1 nor Network 18 laterality significantly associated with non-verbal ability (NVDQ). Network 1 laterality did not significantly associate with NVDQ (*β* = 0.00090 per NVDQ point, SE = 0.00049, t = 1.85, df = 254.0, *p* = 0.066, standardized *β* = 0.115, Fig. 7E), nor did Network 18 (*β* = 0.00055 per NVDQ point, SE = 0.00069, t = 0.79, df = 247.2, *p* = 0.430, standardized *β* = 0.0482, Fig. 7F). Associations in the same direction were observed in the longitudinal dataset at age 2-3 years (Dataset 1), but statistical significance was reduced potentially due to a smaller sample size, a more restricted age range centered around 2–3 years, and the use of the Bayley Scales of Infant and Toddler Development language composite as a proxy for verbal ability (Supplementary Fig. 28**;** Supplementary Note 6). No outcome based exclusion was applied based on the results from outlier analysis (Supplementary Table 3**;** Supplementary Note 7), and this brain-behavioral relationship is robust to alternative non-parametric permutation tests (Supplementary Note 8).

## Discussion

Many existing studies have demonstrated residual differences in functional network organization across adult individuals despite anatomical alignment^7–12^. With young children, there may be even more variability in functional template localization, given the substantial amount of developmental changes in the brain during this period^60^. Smaller and more variable networks risk being obscured by the averaging of anatomically matched locations, and templates created from averaging within a generic network mask will be contaminated by signals from neighboring networks in individual subjects.

By design, individual networks generated by template matching and their population consensus resemble the network priors used to create templates. Although some studies have tried to derive infant-specific network templates^29^, they were still using a coarse adult network atlas to derive the templates. Moreover, a considerable amount of evidence suggests that there are finer network divisions in individuals than are present in many commonly used adult network atlases^8,17,52,61–63^. Additional network detail may help capture subtle changes in development.

Here, we created templates directly on concatenated data instead of group-average data. By avoiding averaging FC at matching anatomical locations across individuals, we uncovered functional network patterns that are more representative of the individual FC profiles assigned to that network. These templates also produced better separation of neighboring networks. Our findings improved upon the current standard practice of averaging FC data across individuals, even when the goal is to find group-level functional networks. For example, the population consensus division of the anterior temporal lobe reported here is more consistent with previous observations in adults^8,10,47,51,64–67^, compared to the divisions identified directly in a group-average FC^5^. Even though we did not have secondary data like task fMRI and DTI to directly verify the biological validity of our functional network divisions, we did observe alignment between our networks and biologically relevant areas that increased activation in some task-based experiments in adults (Supplementary Table 1).

Approximately 10 minutes of data can yield a moderate reliability of NMI = 0.46 in individual networks created from longitudinal sessions at 2 and 3 years old, but a longer scan than 10 min further increases the reliability of individual-specific networks up to and likely beyond 20 min (Supplementary Fig. 26). Our results suggest that scan time per individual and data quality persist as a challenge in precision brain mapping^17,51^, echoing claims from one recent study advocating for longer scan times per participant^68^. We reason that while high-quality data samples with many hours of fMRI data can be useful for data-driven exploration of individual-specific networks^10,13,17,53^ and within-subject study designs^69,70^, datasets with a more moderate amount of data (e.g., 30 min to an hour, following prior recommendations^68^) per participant in a relatively large number of participants may be used to increase the quality of group prior-based mapping of individual-specific networks. In line with this idea, recent papers found that a more extensive data collection is required to reach FC reliability in pediatric samples compared to adults, with diminishing returns after 40–50 minutes of post-censoring data^71,72^. Since precise characterization of individual functional networks has been demonstrated to have clinical significance, such as improving the efficacy of targeted brain stimulation^73–75^, fast precision functional mapping with a moderate scan length using a network template offers a scalable solution for unique populations, broadening its potential applications. Further validation is required to assess the utility and data required of prior-based network mapping in those applications.

We observed a link between functional network lateralization in the networks implicated in language functions and children’s behavioral measures of language performance, beyond the effect of age. In contrast to the strong association between Network 1 (more temporal) lateralization and the verbal developmental quotient, the association for Network 18 (more frontal) is relatively weaker and statistically insignificant. This finding is consistent with prior studies in school-age children where greater resting-state laterality in the temporal gyrus but not the inferior frontal gyrus, has been positively correlated with linguistic skills^76^. This observation might be explained by the additional domain-general processing function of the IFG and MFG beyond language processing^57,58^. While these findings alluded to the behavioral significance of the individual-specific functional networks and their potential utility in clinical applications in young children, such as presurgical or brain stimulation site planning, further validation is required to assess the amount of data needed for a reliable application of the individual-specific networks. Additionally, task-based activation is required to confirm the correspondence between the resting-state network topography and the functional co-localization.

### Functional network assignments are more consistent at sensory cortices than association cortices

Consistent with prior results in infants/toddlers^77–79^, school-age children^16,24^, and adults^9,11–13,25,26,51,54,80,81^, we observed higher inter-individual variability of functional network topography in the association cortices than the sensory cortices in young children (Fig. 4). As described in prior works^35,82^, this spatial bias in inter-individual variability may have a developmental origin: early maturation in some areas limits future plasticity. Additionally, the early-developing sensorimotor areas may be more evolutionarily primitive^82^ and therefore genetically determined to be more consistent across human individuals. One recent paper examining the cell-type abundance in resting-state network divisions in post-mortem brain tissues seemed to support both ideas: the authors observed that sensorimotor network affiliations were better predicted by cell-type than association network affiliations, suggesting more stereotypical cell-type profiles in the sensorimotor networks^83^. The contribution of each factor to this observation is awaiting future exploration.

One large difference between extant neuroimaging data in young children and other populations is that most young children were scanned during natural sleep. A large amount of literature illustrates the differences in FC during sleep and those estimated during resting wakefulness^84–88^, and even across different sleep stages^89,90^. Babies sleep much longer than adults, with up to 16 h of sleep per day in neonates and 12 h in 2-to-3-year-olds. Additionally, the sleep pattern is also different in neonates under 2 months of age, with up to 50% of sleep time spent in the REM/active sleep stage, compared to around 20% in adults^91^. Therefore, some of the developmental differences described here are potentially attributable to differences in the sleep-stage composition of fMRI data across different developmental stages.

While our choice of clustering on FC profiles with area parcels as seeds increased the computational efficiency substantially, it may have averaged out some fine spatial detail, limiting the number of functional networks that can be reliably identified across the population. Additionally, more extensive validation with task fMRI^39,51,92^ and behavioral phenotype prediction^16,17,25^ in larger cohorts with richer clinical representation will be important for further assessing the behavioral and clinical relevance of the proposed individual-specific functional networks. Such analyses may help determine whether individualized network representations can reliably distinguish clinical populations or identify early biomarkers of atypical brain development. Direct evidence from diffusion tensor imaging and cytoarchitecture would also be useful in validating the biological relevance of the identified networks. While we assign a single network label to each vertex for simplicity, network boundaries tend to exhibit more ambiguous assignments, and others have described this organization in terms of overlapping networks^7^.

Even though we only assigned cortical vertices to functional networks in the current study, the conceptual template matching framework is transferable to subcortical network assignments^67,93^. Moreover, functional profiles based on task/movie responses^94–96^ could be utilized to further increase the reliability of the mapped network.

## Methods

### Datasets

We used two high-quality infant/toddler datasets spanning approximately 1–5 years (total *N* = 328 participants) sampling distinct socioeconomic backgrounds for the current study to ensure robustness of the findings. An overview is provided in Table 1, and details of the demographics, acquisition, and processing of the data were reported in other studies with slight differences in the selection of final samples to include^5,97^ and are briefly recapitulated below. Age and sex distributions are provided in Supplementary Fig. 2 (Dataset 1) and Supplementary Fig. 24 (Dataset 2).

**Table 1:** Dataset summary

Datasets	Number of Participant	Number of Scan Sessions
Dataset 1: eLABE	150	199
Dataset 2: BCP	178	301

#### Dataset 1: early life adversity, biological embedding (eLABE)

##### Demographics

We used the Y2 (approximately 2 years old) and Y3 time (approximately 3 years old) point data from the eLABE study with a longitudinal data collection design as our discovery dataset, with basic demographic information summarized in Supplementary Fig. 2. This study was approved by the Human Studies Committees at Washington University in St. Louis, and informed consent was obtained from a parent of all participants. Recruitment oversampled mother-infant pairs facing adversity (e.g., poverty and stress). At each time point, all participants with at least 8 min of usable low-motion fMRI data and no major quality issue in the structural or functional MRI were included, resulting in a total of 150 participants and 199 sessions, of which 49 individuals had both Y2 and Y3 sessions. A subset of 81 sessions (40 from Y2 and 41 from Y3) from healthy term-born toddlers had more than 20 min of usable low-motion data were used to generate the network templates, and vertexwise assignments of individualized networks were created for all sessions. Since we wanted to sample a diverse demographic for our results and to increase statistical power, we did not exclude the 20 preterm-born subjects.

##### MRI data acquisition

MRI data were acquired during natural sleep without sedating medications using a 3 T Prisma scanner (Siemens Corp.) and 64-channel head coil loosely following HCP-style acquisition parameters. During each scan session, a T1-weighted image was collected (sagittal, 208 slices, 0.8 mm isotropic resolution, repetition time = 2400 ms, echo time = 2.22 ms). Functional imaging data (fMRI) were collected using a blood oxygen level–dependent (BOLD) gradient-recalled echo-planar multiband sequence (72 slices, 2.0 mm isotropic resolution, echo time = 37 ms, repetition time = 800 ms, multiband factor = 8, 420 volumes) for a total of 2-9 runs (5.6 minutes each). Spin-echo field maps (at least 1 anterior–posterior and 1 posterior-anterior) were obtained during each session with the same parameters. Framewise Integrated Real-time MRI Monitoring (FIRMM) software suite was used to monitor motion summary in realtime^98^.

##### MRI data preprocessing

Anatomical scan processing and segmentation were conducted using Freesurfer 7.2. Field distortion correction was performed using the FSL TOPUP toolbox. Functional data preprocessing followed established procedures^99^, which includes the basic correction of intensity differences attributable to interleaved acquisition, bias field correction, intensity normalization of each run to whole-brain mode value of 1000, linear realignment within and across runs to compensate for rigid body motion, and linear registration of BOLD images to the adult Talairach isotropic atlas performed in one single step that includes the BOLD to individual T1 to group-average T1 from this cohort to 711-2 N Talairach atlas. Subsequently, volumetric resting-state BOLD time series were projected to fs_LR-32k aligned surfaces for each individual using spherical registration and smoothed with geodesic 2D Gaussian kernels (σ  =  2.25 mm)^100^. Additional fMRI preprocessing to mitigate the effect of motion and other non-neuronal sources of signal was conducted with (i) demean and detrend, (ii) nuisance regression including white matter, ventricles, extra-axial cerebrospinal fluid, and the whole brain mean signals and the 24-parameter Friston expansion regressors derived from the 6 rigid body realignment parameters (x, y, z, pitch, yaw, roll), (iii) bandpass filtering to 0.005-0.1 Hz^99^ after the BOLD data was mapped to surface. Framewise displacement (FD) was calculated to quantify motion within each session and to create a motion-censoring time mask. Specifically, the 6 rigid body realignment parameters were first filtered with an age-specific respiratory notch filter (0.27-0.5 Hz) to reduce the impact of artifactual motion introduced by B0 field perturbation^101,102^. FD was then calculated as the sum of the absolute values (L1-norm) of the differentiated notch-filtered 6 rigid body realignment parameters (by backward differences) at every time point, with the FD of the first frame set to 0 by convention. fMRI data were then censored based on a threshold of FD_filt_ > 0.2 mm, with the additional restriction that only epochs of at least 3 consecutive frames FD_filt_ < 0.2 mm were included, and the first 5 frames were also excluded. Lastly, due to imperfect registration of the medial wall, a small subset of vertices were missing data at the hippocampal regions, and were imputed by the mean value of the nearest neighbor using a custom MATLAB script.

#### Dataset 2: baby connectome project (BCP)

##### Demographics

Full-term (gestational age of 37-42 weeks) infants free of any major pregnancy and delivery complications were recruited as part of the Baby Connectome Project^103^. All procedures were approved by the University of North Carolina at Chapel Hill and the University of Minnesota Institutional Review Boards. Informed consent was obtained from the parents of all participants. In the final cohort used following fMRI data quality control, we retained 301 sleeping fMRI sessions from 178 individuals acquired during natural sleep.

##### MRI data acquisition

All MRI images were acquired on a Siemens 3 T Prisma scanner with a 32-channel head coil at the University of Minnesota and the University of North Carolina at Chapel Hill during natural sleep without the use of sedating medications. T1-weighted (TR = 2400 ms, TE = 2.24 ms, 0.8 mm isotropic; flip angle = 8°), T2-weighted images (TR = 3200 ms, TE = 564 ms, 0.8 mm isotropic), spin echo field maps (SEFM) (TR = 8000 ms, TE = 66 ms, 2 mm isotropic, MB = 1), and fMRI data (TR = 800 ms, TE = 37 ms, 2 mm isotropic, MB = 8) were collected. A mixture of Anterior→Posterior (AP) and Posterior→Anterior (PA) phase encoding directions was used for fMRI acquisition in each session, but they were concatenated into one time series. A subset of data (88 sleeping sessions) had a 720-ms TR instead of an 800-ms TR.

##### MRI data preprocessing

Data processing used the DCAN-Labs infant-abcd-bids-pipeline (v0.0.22), which largely follows the HCP processing^104^ and the steps described previously for the ABCD dataset^105^. The processing steps were similar to the adult data except for the use of infant-specific MNI templates at ages 08-11/11-14/14-17/17-21/21-27/27-33/33-44/44-60 months^106^ to better register the structural data. In addition, segmentation of the brain structures was conducted with Joint Label Fusion (JLF). The toddler-specific brain mask and segmentation were substituted for the Freesurfer^107^ pipeline to refine the white matter segmentation and guide the FreeSurfer surface delineation for each individual scan session of each participant. The native surface data were then deformed to the fs_LR-32k template via a spherical registration.

Functional data processing was largely the same as before, except for the following minor differences: (i) across-run intensity normalization to a whole-brain mode value of 10,000 instead of 1000. (ii) An infant MNI template was used for registration. (iii) fMRI BOLD volumes were sampled to native surfaces using a ribbon-constrained sampling procedure available in Connectome Workbench and then deformed and resampled from the individual’s “native” surface to the fs_LR-32k surface with minimal smoothing at σ = 0.85 mm. Additional steps to mitigate the non-neuronal sources of artifact was conducted on the surface data similar to Dataset 1 with a few minor differences: (i) the mean signal of extra-axial cerebrospinal fluid was not included in the regression, and the mean signals of the 91k grayordinates substituted the whole-brain signal in the nuisance regression, the first-order derivative of the white matter, ventricular CSF and been grayordinate signals were also included for nuisance regression (ii) a respiratory notch filter (0.28-0.48 Hz)^101,102^ was applied to the motion parameters estimates before both FD calculation and the construction of the 24-parameter Friston expansion regressor. (iii) fMRI data were then censored based on a threshold of FD_filt_ > 0.2 mm, and the first seven frames were also excluded. Subsequently, outlier frames whose across-grayordinate standard deviation was more than three median absolute deviations from the median of all frames included, and individual runs were further excluded from a very small subset of sessions due to failure of the visual quality check. A further smoothing with a geodesic 2D Gaussian kernel (σ = 2.40 mm) was applied to give a final effective smoothing of σ = 2.55 mm. We then focused on only the cortical vertices on the fs_LR-32k surface (59412 after excluding medial masks) for the subsequent analyses.

### Functional connectivity representations

Analyses were conducted using an age-specific cortical parcellation previously demonstrated to fit this developmental period^5^. Parcels refer to anatomically defined cortical regions from this parcellation. Vertices refer to surface mesh points on the cortical surface. Two types of FC representations were used, each serving a distinct analytical role. Parcel-to-parcel FC was used to identify reproducible connectivity patterns across individuals. Vertex-to-parcel FC was used to assign network identities to individual cortical vertices based on data-driven network templates. In both cases, FC was defined as the Pearson correlation between BOLD time series and computed across the neocortex in both hemispheres.

### Identification of age-appropriate functional connectivity patterns

For each fMRI session, a parcel-by-parcel FC matrix was computed. Each parcel’s connectivity profile to all other parcels (i.e., one row of the parcel-to-parcel FC matrix) was treated as a single parcel-wise FC profile. In this representation, parcels serve both as samples (rows) and as features (connectivity targets). Parcel-wise FC profiles from all sessions were concatenated across individuals prior to clustering. The analysis included 81 fMRI sessions from 72 unique children aged 2–3 years (9 children contributed longitudinal data at Y2 and Y3; all sessions contained >20 minutes of low-motion data; Dataset 1). Clustering was performed using K-means with correlation distance. The resulting cluster centroids represent reproducible parcel-wise FC patterns and were used as age-appropriate reference network profiles. The initial centroid position was determined by the K-means + + algorithm (MATLAB default). Because the K-means solution is susceptible to the initial centroid location, we set the number of times to repeat the clustering to 1000 to minimize the possibility of stopping at a local minimum, and the final result was the global minimum solution from the 1000 repeats. The K cluster centroids were subsequently used as network templates for template matching.

### Selection of the number of clusters

The number of clusters (K) was selected using a split-half stability analysis. The 81 sessions were randomly divided into two halves 20 times. For each split, parcel-wise FC profiles were concatenated separately and clustered using k-means with K ranging from 1 to 40. Cluster stability was quantified as the normalized mutual information (NMI) between cluster assignments predicted across split halves. A local maximum in stability was observed at K = 23, which was selected for all subsequent analyses. This choice favors sensitivity to finer-grained connectivity patterns, including patterns that may be consistently expressed in only a subset of individuals.

### Individual-specific network mapping

To derive individual-specific network organization at finer spatial resolution, reference network profiles were mapped onto the cortical surface at the vertex level. For each session, vertex-to-parcel FC was computed by correlating the BOLD time series of each cortical vertex with the mean BOLD time series of each parcel, yielding a vertex-wise FC profile for every vertex. Each vertex-wise FC profile was compared with the parcel-level reference network profiles using Pearson’s correlation. Vertices were assigned to the network with the highest correlation using a winner-take-all procedure, similar to prior studies^22^. No magnitude thresholding or distance-based exclusion was applied to the FC profiles. We retained all connections, including weak and anticorrelated values, as these may contribute to network differentiation, particularly in early development.

### Reliability of individual-specific functional networks

One way to measure reliability is to compare the individual-specific functional networks generated using different data samples from the same session. We split the number of low-motion frames into the first half and the second half of the session and calculated two functional connectomes. Then, we generated the individual-specific functional networks from the two functional connectomes and compared them using the normalized mutual information (NMI). Similarly, we generated individual-specific functional networks from the two functional connectomes derived from fMRI data at the Y2 and Y3 time points for the same individual to examine longitudinal reliability.

### Nodal versatility

Nodal versatility (V)^55^ measures whether a node is consistently assigned (V ≈ 0) to a specific community, or it is inconsistently assigned to different communities (V » 0) across different partitions (in our case, different individual networks).

### Lateralization of individual-specific functional networks

Since the fsLR-32k surface template does not sample the gyral and sulcal surfaces equally, to get an accurate measure of the individual-specific functional network size, we calculated the surface area of each vertex using the Connectome Workbench (version 1.2.3) command “wb_command -surface-vertex-areas” using the participant’s midthickness surface obtained from the structural data in the same scan session. The sizes of a given network in each of the left and right hemispheres were calculated as the sum of the surface area of all vertices assigned to that network in the individual in the corresponding hemisphere. A lateralization index was then calculated as the difference in network sizes in the left hemisphere and right hemisphere, divided by the total network size.

### Behavioral measures: Mullen scales of early learning (MSEL)

Mullen Scales of Early Learning^59^ was a standardized assessment of language, motor, and perceptual abilities for children through 5 years of age. There are 5 subdomains: 1) gross motor (GM), 2) fine motor (FM), 3) visual reception (VR), 4) receptive language (RL), and 5) expressive language (EL). The assessment takes 20-45 minutes and was implemented at every behavioral visit between 3 and 60 months of age^103^. We calculated verbal and non-verbal developmental quotient scores (VDQ and NVDQ) as defined by the formula below with the age-equivalent (AE) scores from the subdomains^108^:

1 $${VDQ}\,=\,\left. \left({receptive}\,{language}\,{AE}\,{score}\,+\,{expressive}\,{language}\,{AE}\,{score}\right)/2\right)/{age}_{months} * 100$$

2 $${NVDQ}\,=\,\left. \left({visual}\,{reception}\,{AE}\,{score}\,+\,{fine}\,{motor}\,{AE}\,{score}\right)/2\right)/{age}_{months} * 100$$

### Calculating similarity between networks with spatial probability and FC

To put our 23 functional networks in the context of prior literature and determine the hierarchy in the functional networks, we adapted a method from a prior paper to calculate a similarity measure using both the spatial overlap and FC^17^. Specifically, the overall similarity is calculated as the product of FC similarity and spatial overlap. In the original code base, binarized infomap community topography was used so the spatial overlap was calculated as the average spatial probability covered by that community. In our study, our network topography was specified as continuous (probability, ranging from 0 to 1) rather than binarized value, so the spatial overlap of each of our 23 networks and the Lynch network priors was modified to be calculated as the summed product of the spatial probability of that network and the Lynch network priors, divided by the sum of the spatial probability of that network, similar to the conditional probability formula. The final script “pfm_identify_networks_precalculated.m” is provided (See code availability).

### Quality of separation of the FC profiles

To measure the quality of separations into clusters defined by the network assignments from the group-average FC and the K-means optimized assignments, we use the silhouette index (SI) metric^109^ for each individual FC profile defined as:

3 $${SI}\,=\,\frac{b-a}{\max (a,\,b)}$$

where a is the mean correlation distance (i.e. 1-correlation) to all other FC profiles within the assigned cluster, and b is the mean correlation distance to all FC profiles within the best alternative cluster. The SI ranges from +1 to -1 with the sign indicating whether the area has a more similar FC profile within the assigned cluster (+) or to FC profiles in an alternative cluster (-). The magnitude indicates the degree of separation between the clusters.

### Statistics and reproducibility

Associations between network laterality and age or behavioral measures were assessed using linear mixed-effects models implemented in R (“lme4”), with fixed effects for predictors of interest and a random intercept for subjects to account for repeated scans. Age was included as a covariate in all models due to its developmental relevance; mean framewise displacement was included in secondary analyses to assess robustness to motion. Model diagnostics of “outliers” included inspection of standardized residuals and subject-level influence using Cook’s distance (Supplementary Note 7); no outcome-based exclusions were applied. Analyses were hypothesis-driven and focused on predefined language-related networks and measures, and *p*-values are therefore reported without correction for multiple comparisons.

### Declaration of generative AI and AI-assisted technologies in the writing process

During the preparation of this work the author(s) used ChatGPT, Deepseek and Claude in order to help with writing style and rephrasing to improve the clarity of the sentences. After using this tool/service, the author(s) reviewed and edited the content as needed and take(s) full responsibility for the content of the published article.

### Reporting summary

Further information on research design is available in the Nature Portfolio Reporting Summary linked to this article.

## Supplementary information

Transparent Peer Review file

Supplementary Materials

Reporting Summary

## Data Availability

The fMRI and phenotypic data supporting this study are available from the corresponding datasets. Data from the Baby Connectome Project can be accessed via the NIH Data Archive (https://nda.nih.gov/edit_collection.html?id=2848). Data from the eLABE study are available upon request at https://eedp.wustl.edu/research/elabe-study/

## Code availability

The analysis code has been made publicly available to ensure reproducibility of the results^110^.
