# Supplementary material for: Commonality and variability in functional networks in children under 5 years old
Source: Commun Biol. 2026 Jul 7;9:1421. doi: 10.1038/s42003-026-10599-w (PMC13719990; doi:10.1038/s42003-026-10599-w)
Supplement: Supplementary file 1 [file 42003_2026_10599_MOESM1_ESM.pdf]

nature portfolio

Peer Review File

Commonality and Variability in Functional Networks In Children Under 5 Years Old

Corresponding Author: Dr Muriah Wheelock

This file contains all reviewer reports in order by version, followed by all author rebuttals in order by version.

Version 0:

Reviewer comments:

Reviewer #1

(Remarks to the Author)
The work done by Tu et al. is about detection of individualized functional networks for infants aged 2-3. The idea of the work is taking concatenated FC profiles with clustering as the group-level initial parcellation or prior, and then comparing individual-level profiles at the voxel/vertex level with these group prior profiles to determine the individual-level parcellations using a winner-take-all strategy. The method is simple and easy to understand, however, my concern is mainly about 1) how to evaluate the individual-level networks, 2) how to test the clinical value of the individual-level networks, both did not fully covered in the current version of manuscript.

The essential, or more important question is that, how to validate the derived individual network? Are these networks really reflect individual correctness? How does the author compare their results with previous methods such as Wang ... Liu*'s iterative method (Nature Neuroscience) and how to directly demonstrate the value of using individualized networks vs. group-level networks in clinical diagnosis (with machine learning for example, as a good demonstration of individualized evaluation)? For example, did they use preterm individuals to test the clinical usefulness?

The reviewer appreciate the authors who use many statistical analyses to "indirectly" test the validity of individualized networks, however, more "direct" tests should be conducted. For example, the aims also consists of the evaluation of lateralization; however, lateralization per se does not qualify to be a good evaluation index, at least it does not mean everything about individualized definition of the functional networks.

Another issue is about the generation of the group-level initial templates. While the authors make strong argument that group average removed a significant amount of information, another point is that by using simple Pearson’s correlation also removes a significant amount of information, especially when regions of interest are one of the final goals for individualized parcellation (e.g., from Fig. 1C to D and F), not even mention that winner-take-all strategy also cause information loss.

It is not a good (at least quantitative) way to compare 23 infants’ networks with adults without any spatial correspondence index. As an original paper, Tab. 1 doesn’t seem to be required in the manuscript. Dorsal attention network and task-control or cingulo-opercular system seem not referring the same network (the latter one is more like salience network that involves ACC and insula). Action mode and context association seem not quite familiar to the field. Please comments on the possible functions of these networks and the way interpretations were made. A paragraph from line 178 describes quantification of the derived 23 fine network divisions, but interestingly, not by comparing with infant data-derived results, but adult-based ones. They use network extensions as criteria and use the result to highlight the necessity of generating infant atlases, but this criterion is not quantitative.

For evaluation of the correctness of individualized network detection, it may not be a good practice to use two sample t tests on NMI indices. Statistical significance does not necessarily mean individual information preservation.

Abstract: What is the exact sample size? N should be specific (not as >300)

Fig. 6 show correlations between imaging results and behavioral scores but these correlations are marginally significant, or with a small effect size (r value), and may not survive multiple comparison corrections.

Reviewer #2

(Remarks to the Author)
In this manuscript entitled “Commonality and Variability in Functional Networks in Children Under 5 Years Old”, Tu et al. investigated the individualized large-scale functional brain networks during early childhood. The authors characterize person-specific network organization and its developmental stability across two independent cohorts of toddlers, providing valuable data on early functional brain architecture. The finding that individualized and behaviorally relevant network patterns, including early language-network lateralization, appear before the age of five offers important insights into the maturation of functional organization in the developing brain. Overall, this is a well-designed study that makes a meaningful contribution to our understanding of early functional network development.
I have only a few minor comments that the authors may consider addressing to further improve clarity.

1. It would be helpful for the authors to further clarify the rationale for selecting K = 23 as the optimal number of networks. According to Supplementary Figure 3, K = 8 also appears to represent a local peak. Moreover, the study defines 23 functional networks in the developing brain, yet large-scale functional organization is still maturing during early childhood. It would therefore be important to discuss how stable these fine-grained network subdivisions are across individuals and sessions, and whether this level of parcellation is biologically meaningful and justified for this age range.

2. Regarding the results reported in lines 344–345, when assessing the associations between children’s verbal and nonverbal skills and network lateralization, it would be helpful to clarify whether permutation testing was performed to validate the statistical significance (e.g., by shuffling the correspondence between children’s verbal and nonverbal skills and network measures).

3. In line 500, the reported number of participants (n = 341) appears to be inconsistent with Table 2 (150 + 178 = 328). Please check and clarify this discrepancy.

4. It would be helpful to include information on the age distribution of participants from each dataset in Table 2.

5. In line 586, please specify which infant MNI template was used and provide the corresponding reference.

Reviewer #3

(Remarks to the Author)
In this study, the authors highlight the importance of individualized brain network extraction in developmental cognitive neuroscience and propose a novel framework for identifying subject-specific functional networks from single resting-state fMRI scans. Their findings suggest that several large-scale brain networks—and their hemispheric lateralization—emerge before the age of five. Notably, they report that left-lateralization within language-related networks is associated with cognitive abilities such as verbal performance. Overall, the manuscript presents an interesting concept with potentially meaningful implications for understanding early brain functional organization. However, the paper exhibits notable limitations in structure, clarity, and methodological transparency, particularly concerning the presentation and interpretation of methods and results. Major concerns include an overly broad and methodologically diffuse (“kitchen-sink”) analytical approach, ambiguously defined network “templates,” insufficient justification for evaluation metrics, and an unclear rationale for dataset selection. The following points outline key issues and recommendations for improvement.

1. Insufficient Dataset Description and Selection Criteria
The manuscript lacks a clear and consistent description of the datasets utilized. The reported sample sizes in the text do not align with those presented in the tables, and no explanation is provided for these discrepancies. The study appears to involve two independent datasets, yet their respective roles and comparative purposes are not clearly articulated. For instance, the authors mention using a subset of the first dataset to build the network template, but it remains unclear how the remaining data were incorporated into subsequent analyses. Moreover, if the study does not aim to examine pathological or atypical populations, the inclusion of certain datasets requires further justification.
2. Clarity of Abstract and Introduction
The abstract is underdeveloped and omits several key elements. For example, it does not explicitly mention the development of functional reference templates or report statistical significance levels. Additionally, some statements in both the abstract and introduction are inaccurate or overstated. The concept of individualized network extraction is not novel per se; numerous approaches—including ICA, PCA, and other data-driven frameworks—can achieve similar objectives. Prior research has also delineated individualized brain networks in children and infants, including hemispheric lateralization patterns, and several age-specific brain templates have been established in earlier work. The authors should more clearly delineate how their approach advances beyond existing methodologies or provides distinct contributions to the field.
3. Presentation of Results
The results section lacks clear organization and coherence, making it difficult for readers to follow the progression of analyses. Critical methodological details—such as the parcellation scheme and the mapping between functional connectivity measures and brain networks—are insufficiently described or introduced too late. As a result, interpretation of the results is substantially impeded. Improved figure organization and clearer linkage between text and visualizations would greatly enhance comprehensibility.
4. Presentation of the Methods Section
The methods section is difficult to follow and would benefit from substantial reorganization and clarification. The authors are encouraged to move detailed preprocessing steps to the Supplementary Information and summarize the key procedures

more concisely in the main text. The sudden introduction of numerical quantities (e.g., “59,412”) without context is confusing. The transition from preprocessing to the construction of the functional connectome lacks clear definitions of critical terms, such as “vertex,” “parcel,” and “vertex-to-parcel connectome.” A schematic flowchart could help guide readers through the analytical pipeline. Moreover, the description of the individual-specific network derivation is unclear. After introducing the network template, the term “seed FC” is ambiguously defined—does this refer to parcel-wise functional connectivity within each scan? The statement “we identified the network template with … to the seed FC” is particularly opaque. It remains unclear how the “individual functional networks” are ultimately derived from the template.

5. Statistical Analysis

The statistical framework is insufficiently described. It is unclear how associations were modeled or whether random effects were included to account for repeated scans within subjects. The manuscript does not specify whether outlier detection or exclusion procedures were applied (as seen clear outliers in the figures), nor does it indicate whether multiple comparison corrections were implemented. Furthermore, the choice of covariates in the regression models is not discussed. These omissions raise concerns regarding the robustness and interpretability of the reported findings.

6. Overall Recommendation

The manuscript would benefit from substantial reorganization, greater methodological transparency, and a more comprehensive review of relevant literature. The introduction and discussion sections should more clearly situate the study within the context of existing work, emphasizing its distinct contributions and methodological advances. A thorough rewrite focused on improving clarity, coherence, and scientific rigor is strongly recommended prior to resubmission.

Version 1:

Reviewer comments:

Reviewer #2

(Remarks to the Author)

The authors have addressed my comments appropriately in the revision.

Reviewer #4

(Remarks to the Author)

In "Commonality and Variability in Functional Networks In Children Under 5 Years Old", Tu and authors deploy and validate an individualized network approach in a cohort of young children. They then utilize this approach to specifically explore lateralization and behavioral associations of the language network. Overall, the paper demonstrates methodological novelty in using a precision functional network approach with such a young subject cohort, providing a useful resource for the field. Furthermore, I found the review and discussion of the existing literature to be very thorough. While I was not a reviewer for the original submission, I see that the authors have made extensive and meaningful changes to address the initial comments. However, I do share some of the previous reviewer’s concerns regarding the clarity, organization, and claims within the manuscript – especially with the behavioral modeling. I have a couple of points that the authors could address to enhance the impact and rigor of the submission.

1) I find the overall organization and flow of the paper to be lacking in clear direction. The choice of modeling language specifically feels a bit arbitrary and unmotivated. Given the wide scope of the dataset generated, a more comprehensive evaluation of networks, metrics (such as lateralization), and behavioral associations is warranted to better motivate the focus on language specifically. Additionally, the emphasis on network definitions impedes the overall narrative and could be condensed. For instance, Table 1 could be moved to the supplemental document and the in-text discussion could be shortened.

2) The language behavior modeling analysis (figure 7) lacks depth and the proper motivation. Ex. Why was the default-anterolateral network chosen specifically to be the comparison? Furthermore, the effect sizes reported are very small with seemingly minimal differences between networks, the wording in the manuscript should better reflect this. A more comprehensive comparison across networks and behavioral measures would help. For instance, a statistically stringent identification/ranking of networks that associate with a given behavior. At the very least, a more detailed explanation for the analysis choices made and a more thorough behavioral comparison of the networks is warranted.

Thank you to the authors for their hard work and an interesting read!

Open Access This Peer Review File is licensed under a Creative Commons Attribution 4.0 International License, which permits use, sharing, adaptation, distribution and reproduction in any medium or format, as long as you give appropriate credit to the original author(s) and the source, provide a link to the Creative Commons license, and indicate if changes were made.
In cases where reviewers are anonymous, credit should be given to 'Anonymous Referee' and the source.
The images or other third party material in this Peer Review File are included in the article's Creative Commons license, unless indicated otherwise in a credit line to the material. If material is not included in the article's Creative Commons license and your intended use is not permitted by statutory regulation or exceeds the permitted use, you will need to obtain permission directly from the copyright holder.
To view a copy of this license, visit https://creativecommons.org/licenses/by/4.0/

We appreciate the reviewers' time and effort to read and comment on our manuscript. Please see below for the response to specific points in blue, with the changes in text in *Italics*.

Reviewers' comments:

Reviewer #1 (Remarks to the Author):

The work done by Tu et al. is about detection of individualized functional networks for infants aged 2-3. The idea of the work is taking concatenated FC profiles with clustering as the group-level initial parcellation or prior, and then comparing individual-level profiles at the voxel/vertex level with these group prior profiles to determine the individual-level parcellations using a winner-take-all strategy. The method is simple and easy to understand, however, my concern is mainly about 1) how to evaluate the individual-level networks, 2) how to test the clinical value of the individual-level networks, both did not fully covered in the current version of manuscript.

The essential, or more important question is that, how to validate the derived individual network? Are these networks really reflect individual correctness? How does the author compare their results with previous methods such as Wang ... Liu*'s iterative method (Nature Neuroscience) and how to directly demonstrate the value of using individualized networks vs. group-level networks in clinical diagnosis (with machine learning for example, as a good demonstration of individualized evaluation)? For example, did they use preterm individuals to test the clinical usefulness?
The reviewer appreciate the authors who use many statistical analyses to "indirectly" test the validity of individualized networks, however, more "direct" tests should be conducted. For example, the aims also consists of the evaluation of lateralization; however, lateralization per se does not qualify to be a good evaluation index, at least it does not mean everything about individualized definition of the functional networks.

We thank the reviewer for these important and constructive comments. It highlights two closely related issues: (1) how to validate the biological meaningfulness of individualized networks in the absence of ground truth, and (2) how to demonstrate their added value relative to group-consensus networks and prior individualized methods. We have substantially revised the manuscript to address these points, as detailed below.

1. Validation of individualized network correctness in the absence of ground truth

We agree that without a definitive ground truth for functional network organization, validation must rely on principled internal and external criteria rather than direct correctness. Therefore, a conservative and widely adopted validation strategy is to test whether individualized networks (i) exhibit coherent internal functional organization and (ii) capture biologically meaningful individual-level information beyond group averages.

To address this directly, we added a new analysis comparing within-network functional connectivity (FC) derived from individualized network topographies versus group-consensus

network topographies (new Figure 5). If a network is correctly delineated, regions assigned to that network should exhibit stronger internal connectivity. The text was updated to read as:

"Within-network connectivity is higher and reflects more age-related variance for individualized network topography compared to the group consensus network topography

So far, we have demonstrated the individual-specificity of the individualized network and the locations of variability. It remains unclear whether the individualized networks were superior to the group-level networks in capturing truly meaningful biologically relevant signals in individuals. If a network is correctly delineated, regions assigned to it should be more strongly connected to each other. Therefore, we compared the within-network FC for networks defined by individualized network topography or the group consensus network topography (from the whole dataset to avoid leakage of age, Y2 and Y3, N = 199, Figure 5A). We found that within-network FC was significantly higher for the individualized network topography compared with the group consensus network topography for 18 out of 23 networks (FDR-corrected p < 0.05, paired t-test). Dorsal Attention, Visual Central and Default-Parietal networks did not demonstrate a significant difference, while Visual Peripheral and Default-Pregenual had a significant lower within-network FC for individualized network topography compared with the group consensus network topography (FDR-corrected p < 0.05). The Visual Peripheral network result is potentially due to the group consensus network capturing the most strongly connected region within the network, which was highly consistent across individuals, but individualized networks sometimes include weaker FC at the boundaries.

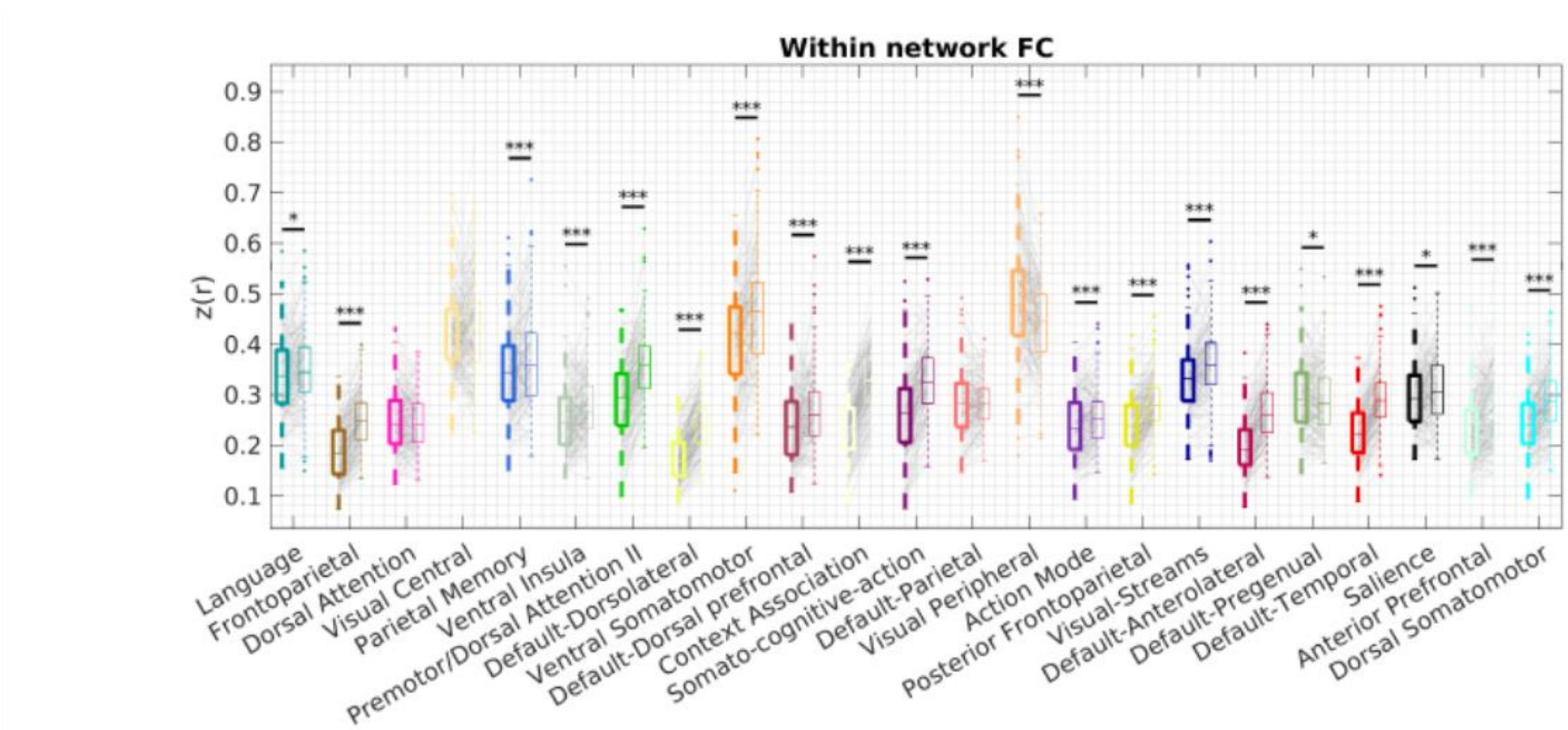

*Figure 5. Within-network FC using network topography defined by group consensus or individualized network. A) Within-network FC with network topography defined by group consensus (bolded, left) or individualized network (right). * p<0.05, ** p<0.01, *** p<0.001. FDR-corrected."*

2. Demonstrating added value beyond group-level networks

To further assess whether individualized networks capture biologically meaningful information beyond motion artifacts and group-level organization, we examined their ability to explain and predict developmental variation. The relevant results were included in the manuscript to read as:

"To assess the developmental information captured by within-network FC beyond motion, we compared models including mean framewise displacement (FD) alone to models additionally incorporating within-network connectivity features. Mean FD alone explained negligible variance in age ($R^2 \approx 0$). Adding the 23 within-network FC measures defined by individualized topographies explained substantially more age-related variance than adding the 23 within-network FC measures defined by group-consensus topographies ($\Delta R^2 = 0.35$ vs. 0.21; adjusted $R^2 = 0.26$ vs. 0.11), despite identical model complexity. To assess subject-level generalization, we performed 5-fold cross-validation grouped by subject using ridge regression, ensuring the different sessions of the same subject fall in the same fold, with mean FD and within-network FC from all 23 networks as predictors. Prediction accuracy was evaluated by aggregating out-of-sample predictions across folds and computing the correlation between predicted and true age. Models using individualized network topographies achieved substantially higher prediction accuracy ($r = 0.435$, $R^2 = 0.190$) than models using group-consensus network topographies ($r = 0.276$, $R^2 = 0.076$), indicating that individualized network representations capture developmentally relevant information beyond group-level network definitions."

3. Clinical validation and preterm classification

We agree that clinical classification (e.g., preterm vs. term-born) would be a valuable demonstration of individualized network utility. We carefully considered this analysis; however, the available dataset includes only 27 sessions from 20 preterm children compared with 172 term-born children, resulting in severe class imbalance and limited statistical power. Under these conditions, classification performance would be highly unstable and difficult to interpret. We therefore elected not to pursue this analysis and instead focused on developmental associations, which are better powered in the current dataset. We now clarify this limitation explicitly in the Discussion and view clinical validation as an important direction for future work in larger cohorts:

"Additionally, more extensive validation with task fMRI (Braga et al., 2020; Du et al., 2024; Nielsen et al., 2023) and behavioral phenotype prediction (Cui et al., 2020; Kong et al., 2019; Lynch et al., 2024) *in larger cohorts with richer clinical representation will be important for further assessing the behavioral and clinical relevance of the proposed individual-specific functional networks. Such analyses may help determine whether individualized network representations can reliably distinguish clinical populations or identify early biomarkers of atypical brain development.*"

4. Comparison with prior individualized parcellation methods

We acknowledge the importance of prior individualized network methods, including Wang et al. (2015) and related iterative approaches. As discussed in the Introduction, many prior-based

methods have substantially advanced individualized network mapping by reducing per-subject data requirements. Our contribution is complementary rather than competitive.

Specifically, our innovation lies in the definition of cohort-appropriate network priors, derived by clustering concatenated functional connectivity profiles rather than relying on group-average FC or predefined adult network templates. This distinction is particularly important in developmental populations, where adult-derived priors may not be appropriate. In principle, our finely divided 23-network prior could be used as input to iterative methods such as Wang et al. (2015). A direct comparison between iterative optimization and template-matching approaches is beyond the scope of the current study, particularly given that the original iterative algorithm was not released publicly and prior attempts to obtain the code did not receive a response from the authors.

Importantly, both approaches rely on priors optimized from data; however, unlike prior methods, our framework does not assume a fixed number or spatial configuration of networks derived from adult populations. The validation analyses added here demonstrate that this strategy yields individualized networks that are internally coherent and more informative for developmental modeling than group-consensus networks."

In summary, while no ground truth exists for functional network organization, we provide converging evidence that our individualized networks (i) exhibit stronger within-network coherence, (ii) capture developmentally meaningful individual differences beyond motion and group averages, and (iii) improve subject-level prediction relative to group-consensus networks. These results directly address the reviewer's concerns regarding validation, comparison to prior methods, and practical value.

Another issue is about the generation of the group-level initial templates. While the authors make strong argument that group average removed a significant amount of information, another point is that by using simple Pearson's correlation also removes a significant amount of information, especially when regions of interest are one of the final goals for individualized parcellation (e.g., from Fig. 1C to D and F), not even mention that winner-take-all strategy also cause information loss.

We thank the reviewer for raising this important methodological point. We agree that, beyond group averaging, additional sources of information loss can arise from the use of Pearson correlation and hard winner-take-all assignment during individual network mapping. These design choices involve trade-offs between interpretability, robustness, and representational richness.

Pearson correlation to the network template was used in individualized network mapping because it provides a scale-invariant measure of functional similarity that is robust to inter-subject differences in signal amplitude, particularly in developmental datasets with variable data quality. More importantly, the network template was on a normalized scale, representing the centers of the clusters by correlational distance, so the choice of a scale-invariant similarity

measure was intentional. Similarly, winner-take-all assignment produces a hard, non-overlapping parcellation that simplifies interpretation and downstream analyses, but necessarily collapses continuous similarity structure and does not represent graded or overlapping network membership.

In practice, ambiguity in network assignment is concentrated near network borders, where multiple networks show similar functional connectivity profiles. We examined assignment confidence, defined as the silhouette index based on correlational distance to the best and second best-match template, and found that most vertices show a clear preference for the assigned network, with low-confidence assignments largely confined to boundary regions (Supplementary Figure 30I-M).

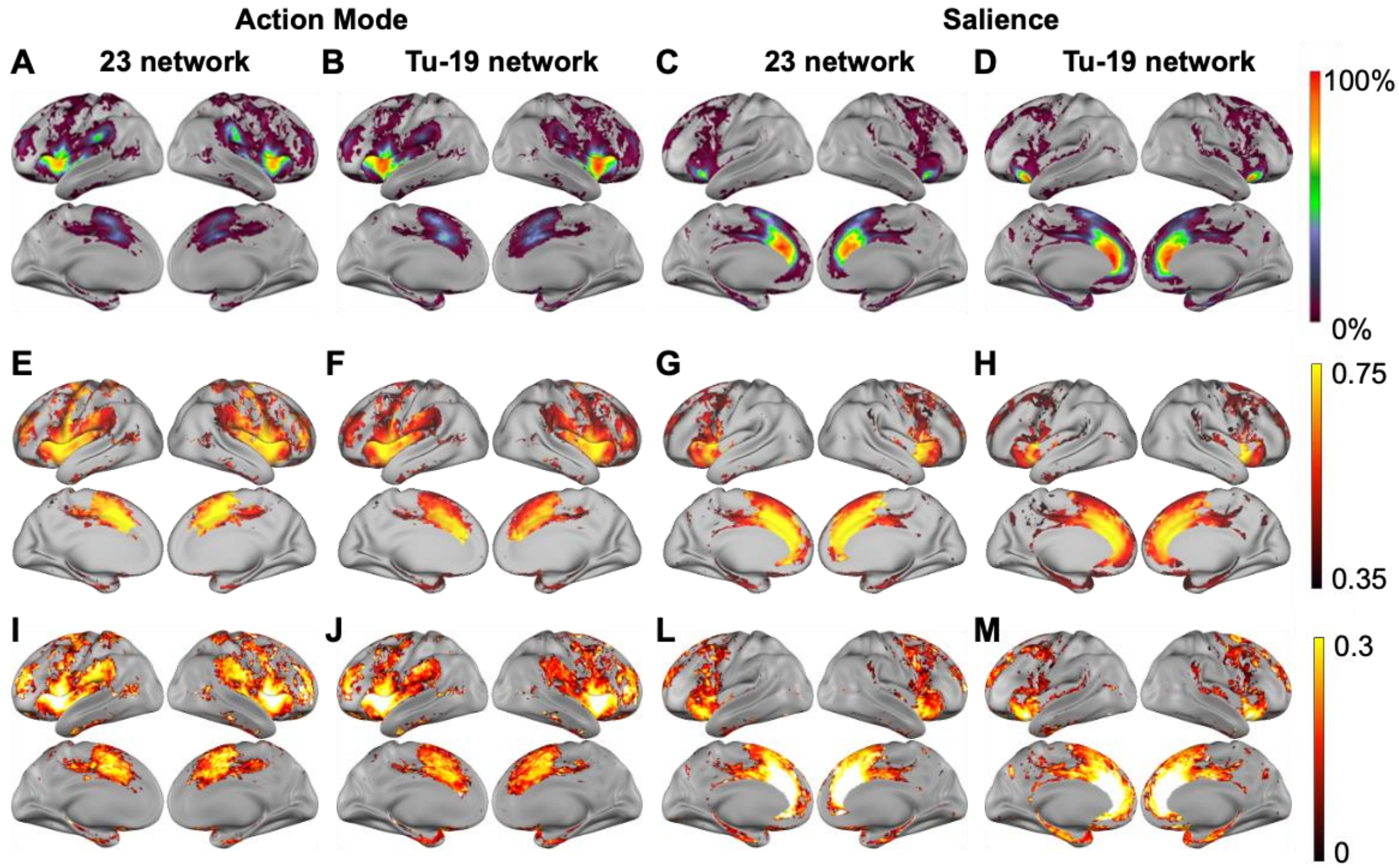

Supplementary Figure 30. *Comparing the network topography of the same network using different network templates*. A-B) Spatial probability E-H) Mean Pearson's correlation to the network template. *I-M) Silhouette Index (Confidence) with correlational distance.*

We emphasize that the use of correlation-based template matching with hard assignment is consistent with prior individualized network mapping approaches, including Wang et al. (2015) and related work cited by the reviewer. Nevertheless, we acknowledge that hard parcellation does not capture overlapping or probabilistic network structure. We now explicitly discuss this limitation in the Discussion and note that future extensions could incorporate soft or overlapping assignments, which reads *"While we assign a single network label to each vertex for simplicity, network boundaries tend to exhibit more ambiguous assignments, and others have described this organization in terms of overlapping networks (Bijsterbosch et al., 2023)."*

To improve clarity and avoid potential confusion between template construction and individualized network assignment, we have reorganized Figure 1 and revised the corresponding Results and Methods sections following Reviewer 3's suggestions.

It is not a good (at least quantitative) way to compare 23 infants' networks with adults without any spatial correspondence index. As an original paper, Tab. 1 doesn't seem to be required in the manuscript. Dorsal attention network and task-control or cingulo-opercular system seem not referring the same network (the latter one is more like salience network that involves ACC and insula). Action mode and context association seem not quite familiar to the field.

We thank the reviewer for this thoughtful comment regarding the interpretation and presentation of Table 1. We agree that, in isolation, qualitative comparison without an explicit spatial correspondence index would be insufficient for quantitatively comparing infant and adult networks. We therefore want to clarify the intent and construction of Table 1, as well as the role it plays relative to our quantitative analyses.

First, we acknowledge that not all previously reported network maps—particularly those from infant and developmental studies—are available in a standardized format suitable for direct quantitative spatial correspondence analysis. Differences in anatomical references, spatial resolution, and in some cases subject-specific representations limit the feasibility of comprehensive quantitative comparisons across all prior studies. For this reason, Table 1 was not intended as a quantitative validation of network equivalence, but rather as a contextual guide linking our identified networks to prior literature.

Importantly, Table 1 was constructed using a combination of quantitative correspondence measures (spatial probability overlap and functional connectivity similarity, described in the Supplementary Materials), meta-analysis of potential network function using neurosynth, and expert visual assessment to complement these measures when quantitative comparison was not feasible. We have revised the manuscript to explicitly state this, adding the following sentence:

"This table was made both with the quantitative spatial and connectivity correspondence measures mentioned above, and with expert visual assessment of spatial correspondence, used to complement the quantitative measures."

We also want to clarify that Table 1 is not asserting one-to-one equivalence between our network labels and any single network label in prior work. Instead, the table lists prior network labels in the literature that partially overlap with each identified network, acknowledging that correspondence may be incomplete or context-dependent. For example, the network we labeled "Dorsal Attention" in early childhood shows partial spatial overlap with regions that have been described under different labels in the literature (including CO1/task-control cingulo-opercular subsystem labels in Gratton et al., 2022). Our intent is not to claim these are the same network, but to provide a parsimonious naming scheme for the present manuscript while helping readers—especially non-specialists—map our labels onto commonly encountered terminology

across studies. For clarity, we revised the Table 1 header to read as "Prior network definitions in literature that overlap with this network".

Finally, while Table 1 necessarily includes a qualitative component, we believe it improves interpretability for an interdisciplinary readership by situating early-childhood networks within the broader literature. We have revised the text to more explicitly delimit the scope of Table 1 and to reduce the risk of overinterpretation.

Please comments on the possible functions of these networks and the way interpretations were made. A paragraph from line 178 describes quantification of the derived 23 fine network divisions, but interestingly, not by comparing with infant data-derived results, but adult-based ones. They use network extensions as criteria and use the result to highlight the necessity of generating infant atlases, but this criterion is not quantitative.

We thank the reviewer for this thoughtful comment regarding the interpretation and validation of the identified networks. We agree that, in the absence of task fMRI or diffusion imaging data, functional interpretations of the networks must be made cautiously and can only be inferred indirectly based on spatial correspondence with previously reported networks in the literature, or meta-analysis of the reported previously reported function with Neurosynth (Supplementary Figures 5-16), with the caveat that many of the reports were from the adult literature. The underlying assumption was that the function of regions has been pre-programmed by the biological circuit (e.g. visual cortex), despite the increased complexity or change in fine-grained representation over development. In the original manuscript, quantitative correspondence analyses were performed primarily against finely divided adult networks derived from highly sampled precision fMRI data (Lynch et al., 2024). The rationale for this choice was methodological rather than developmental: these adult networks provide spatial probability maps and average functional connectivity profiles at a finer granularity than most group-based parcellations, allowing us to test whether the fine subdivisions identified here align with previously described network motifs or instead reflect patterns that may be specific to early childhood. We have now clarified this motivation explicitly in the revised text.

We agree with the reviewer, however, that limiting quantitative comparisons to adult networks does not fully address developmental context, particularly given the growing literature on infant and early-life functional network organization. In response, we have extended our network correspondence analysis (Supplementary Figures 5-16) to include two group-level network atlases at early childhood, enabling direct quantitative comparison between our identified networks and prior infant network definitions. This addition allows us to more explicitly assess which network patterns show continuity with infant organization and which may reflect developmental refinement or reconfiguration during early childhood. The revised texts in Supplementary texts read as:

"For the analysis with the Network Correspondence Toolbox, *nine representative atlases from different research groups were selected as references: "Du2024-15" (Du et al., 2024), "Woodward2024-Task-12"(Percival and Woodward, 2025), "Yan2023-400+Kong2019-17"(Kong*

et al., 2019; Yan et al., 2023), “Gordon2024-5"(Gordon et al., 2023), “Gordon2017-17”(Gordon et al., 2017), “Shen2013-268-8"(Shen et al., 2013) and “Glasser2016-360+Ji2019-12”(Glasser et al., 2016; Ji et al., 2019), “Tu2025-19”(Tu et al., 2025), “Kardan2022-10”(Kardan et al., 2022).”

And also to emphasize our raw numerical reports of the statistics in addition to plots:
“*The significantly overlapping networks in each atlas and the corresponding dice coefficient are visualized in circular bar chart plots (Supplementary Figures 5-16), and the raw dice coefficients and p-value statistics of all networks in all atlases are available in csv files at https://github.com/cindyhfls/Tu-2026-IndividualBabyNetworks/tree/main/Supplementary/NetworkCorrespondenceToolboxResults.*”

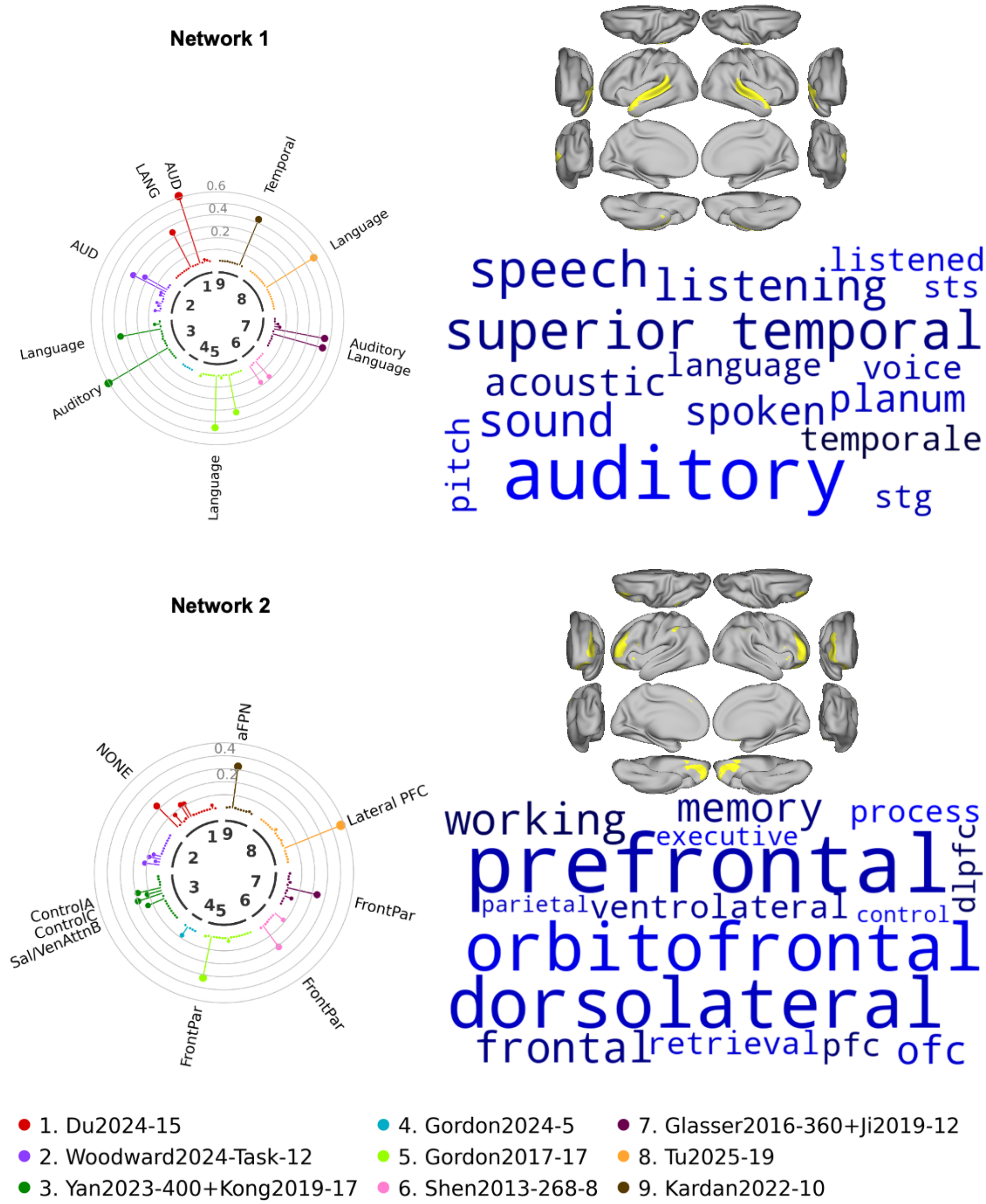

Supplementary Figure 5. *Correspondence between the 23 networks and previously reported functional networks/decoded terms (Networks 1-2)*. Circular bar chart plot from the network correspondence toolbox for spatial correspondence with the network locations with >0.35 probability and 7 atlases from the literature. Circles illustrate the magnitude of the dice coefficient in intervals of 0.1. Only significant networks from the spatial permutation test (p<0.05) were named. Top right: the network locations with >0.35 probability visualized on a template brain surface. Top bottom: terms with highly correlated meta-analytic activation maps to the network topography.

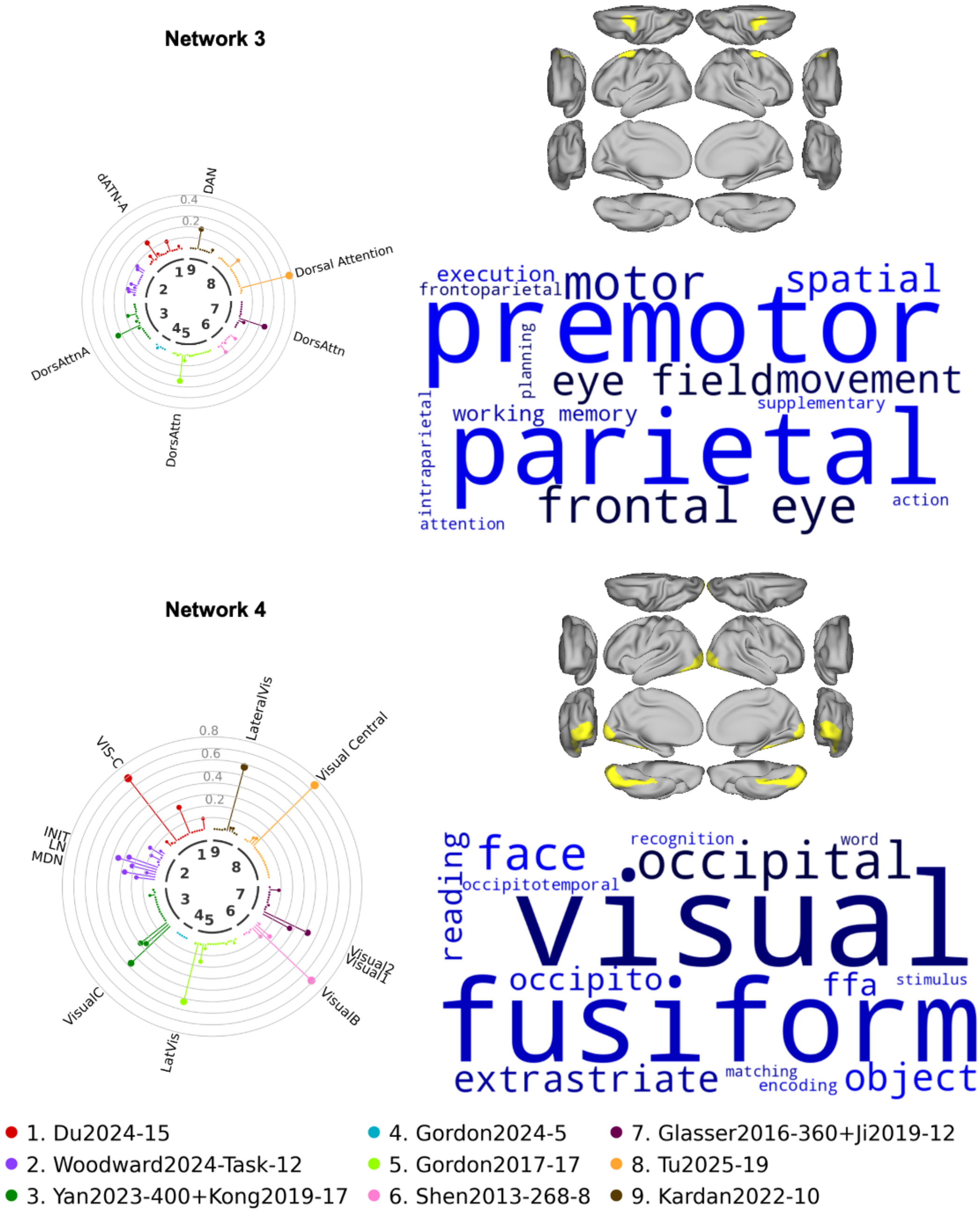

Supplementary Figure 6. *Correspondence between the 23 networks and previously reported functional networks/decoded terms (Networks 3-4)*.Left: Circular bar chart plot from the network correspondence toolbox for spatial correspondence with the network locations with >0.35 probability and 7 atlases from the literature. Circles illustrate the magnitude of the dice coefficient in intervals of 0.1. Only significant networks from the spatial permutation test (p<0.05) were named. Top right: the network locations with >0.35 probability visualized on a template brain surface. Top bottom: terms with highly correlated meta-analytic activation maps to the network topography.

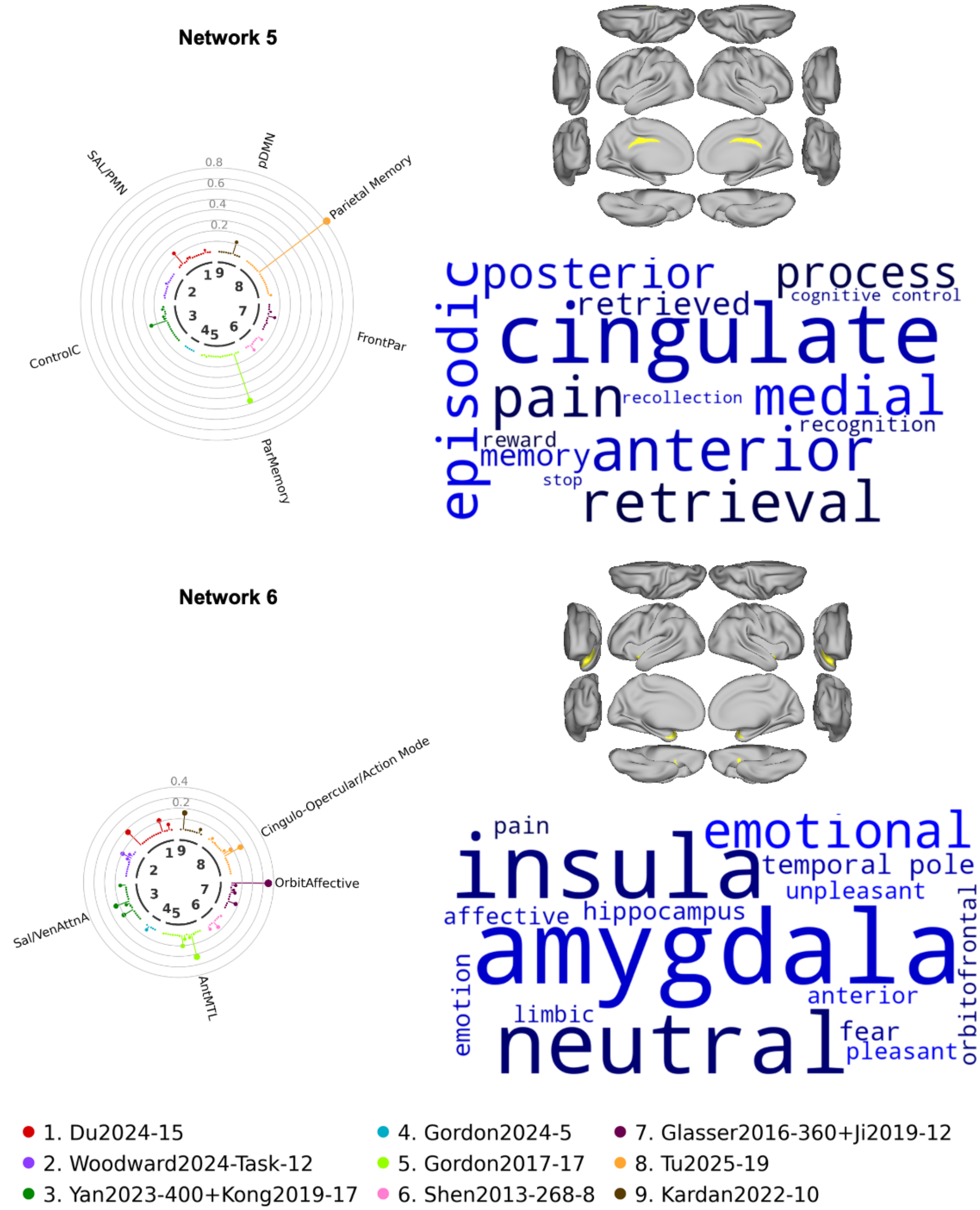

Supplementary Figure 7. *Correspondence between the 23 networks and previously reported functional networks/decoded terms (Networks 5-6)*. Left: Circular bar chart plot from the network correspondence toolbox for spatial correspondence with the network locations with >0.35 probability and 7 atlases from the literature. Circles illustrate the magnitude of the dice coefficient in intervals of 0.1. Only significant networks from the spatial permutation test (p<0.05) were named. Top right: the network locations with >0.35 probability visualized on a template brain surface. Top bottom: terms with highly correlated meta-analytic activation maps to the network topography.

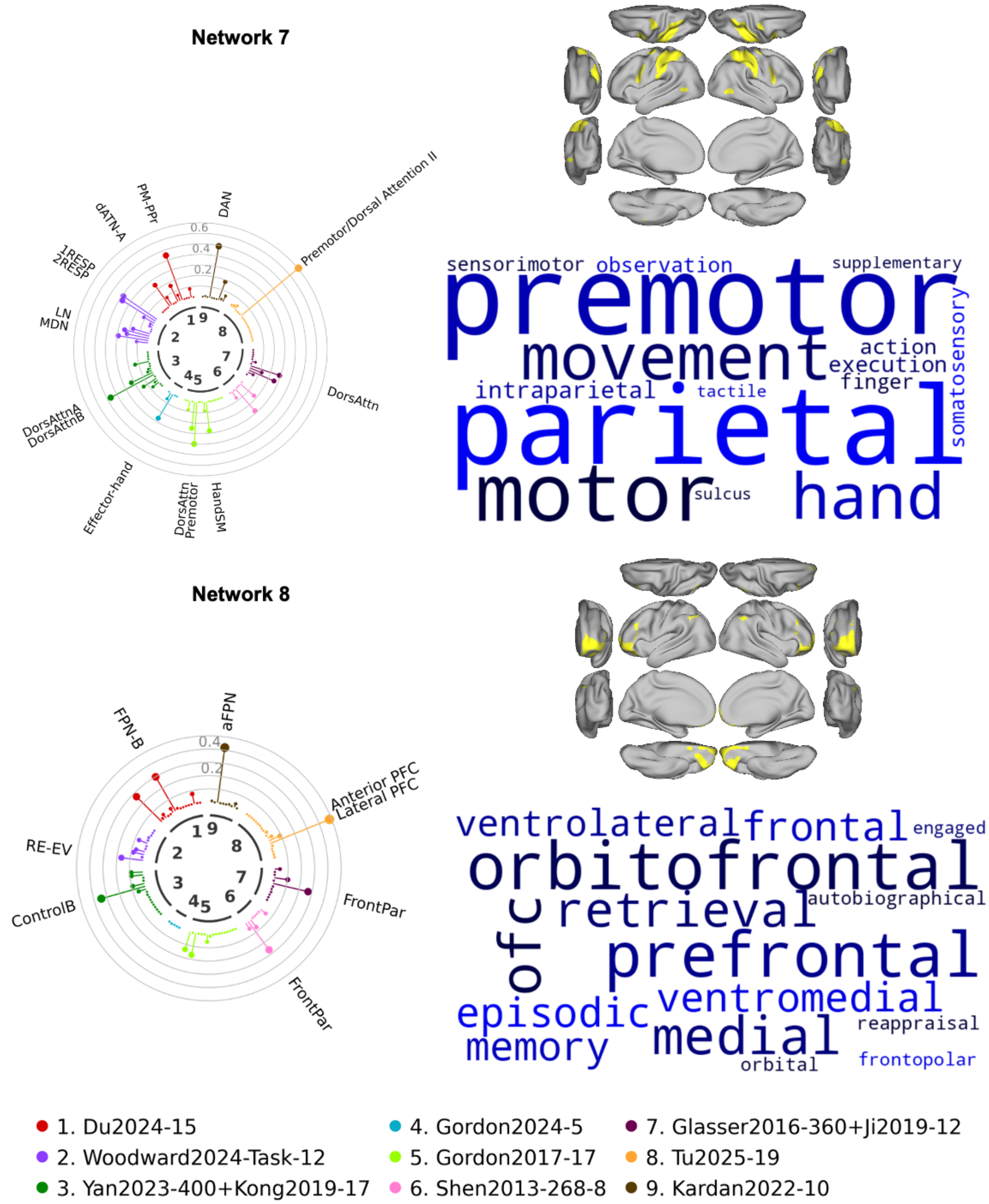

Supplementary Figure 8. *Correspondence between the 23 networks and previously reported functional networks/decoded terms (Networks 7-8)*. Left: Circular bar chart plot from the network correspondence toolbox for spatial correspondence with the network locations with >0.35 probability and 7 atlases from the literature. Circles illustrate the magnitude of the dice coefficient in intervals of 0.1. Only significant networks from the spatial permutation test ($p<0.05$) were named. Top right: the network locations with >0.35 probability visualized on a template brain surface. Top bottom: terms with highly correlated meta-analytic activation maps to the network topography.

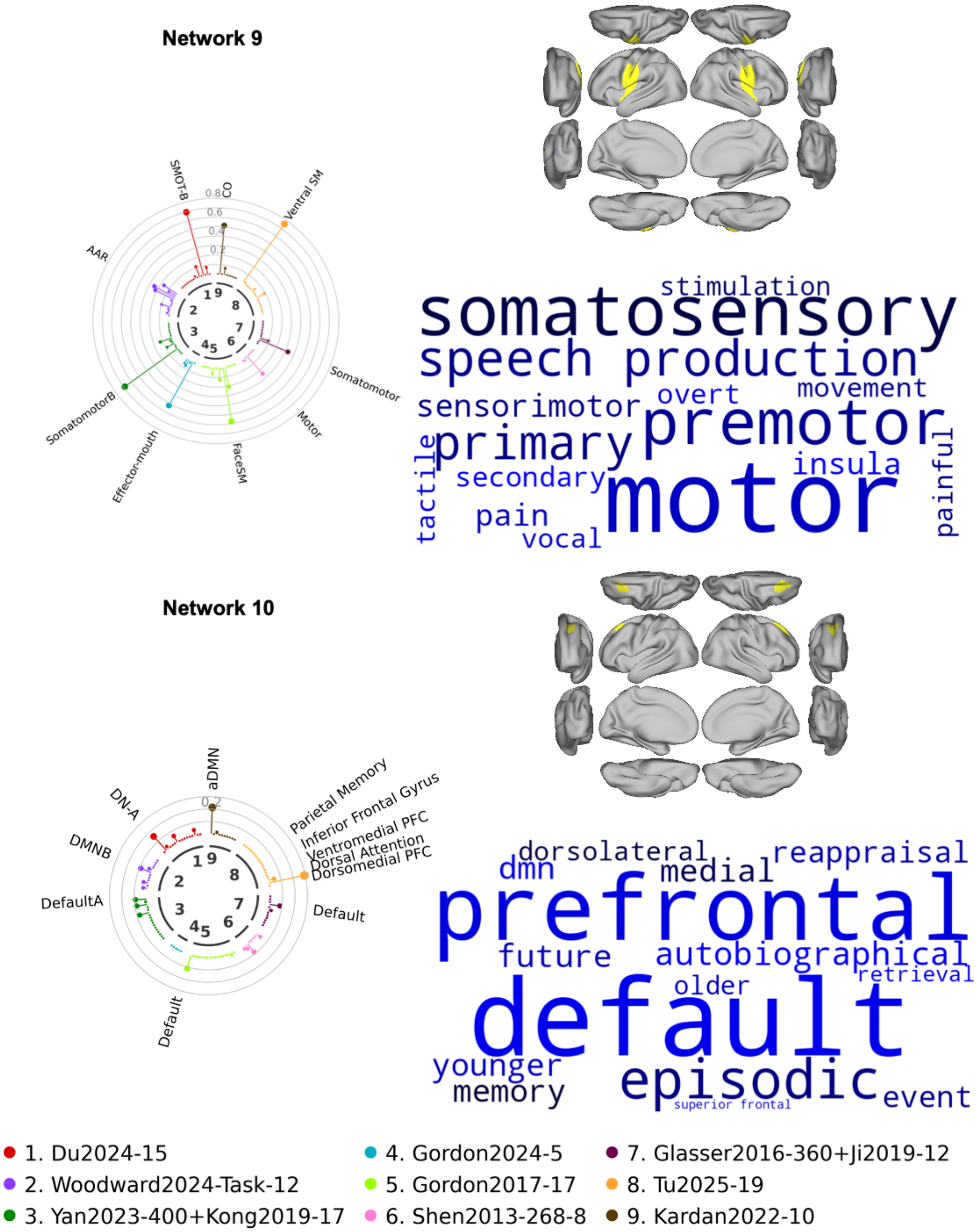

Supplementary Figure 9. *Correspondence between the 23 networks and previously reported functional networks/decoded terms (Networks 9-10)*. Left: Circular bar chart plot from the network correspondence toolbox for spatial correspondence with the network locations with >0.35 probability and 7 atlases from the literature. Circles illustrate the magnitude of the dice coefficient in intervals of 0.1. Only significant networks from the spatial permutation test ($p<0.05$) were named. Top right: the network locations with >0.35 probability visualized on a template brain surface. Top bottom: terms with highly correlated meta-analytic activation maps to the network topography.

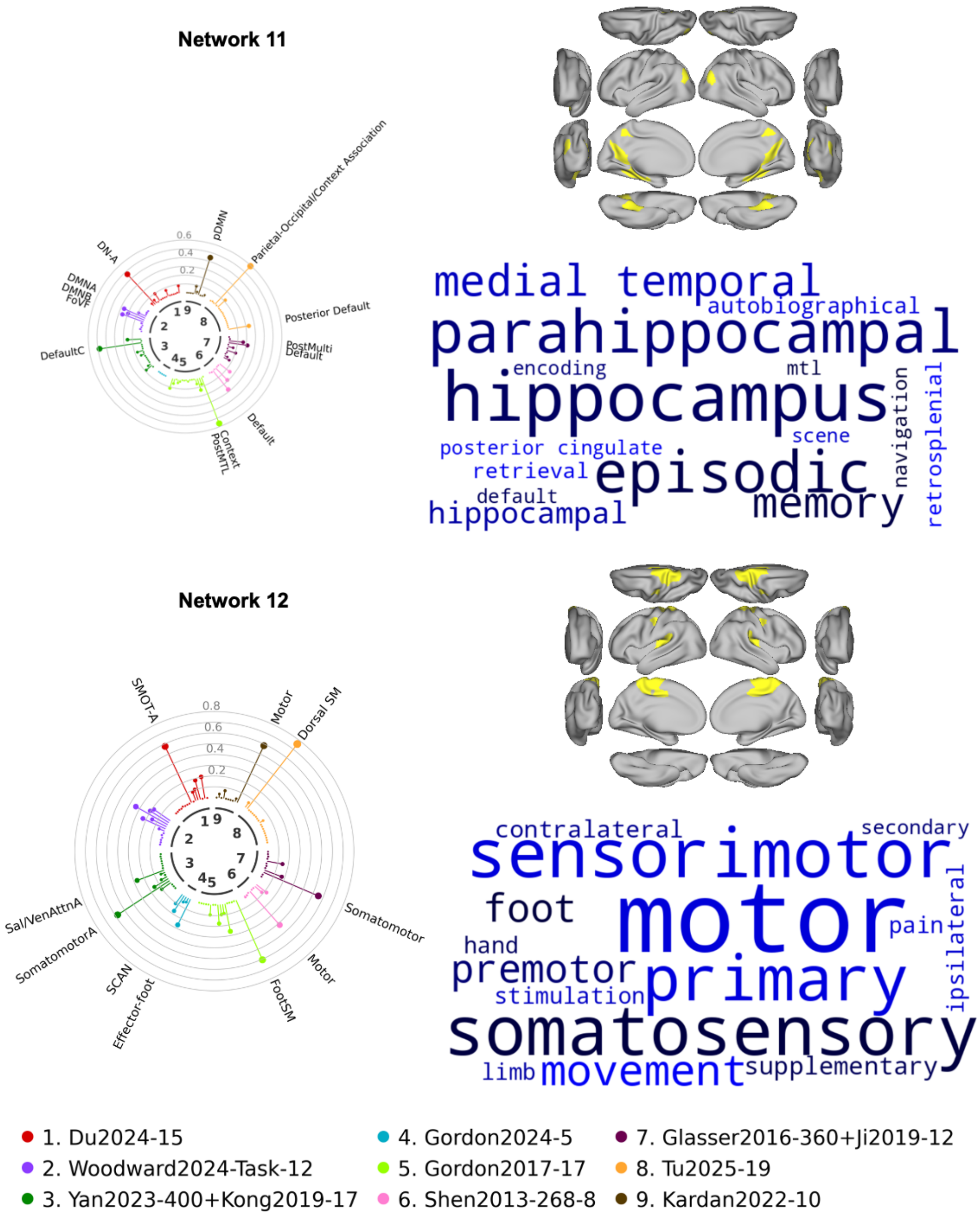

Supplementary Figure 10. *Correspondence between the 23 networks and previously reported functional networks/decoded terms (Networks 11-12)*. Left: Circular bar chart plot from the network correspondence toolbox for spatial correspondence with the network locations with >0.35 probability and 7 atlases from the literature. Circles illustrate the magnitude of the dice coefficient in intervals of 0.1. Only significant networks from the spatial permutation test ($p<0.05$) were named. Top right: the network locations with >0.35 probability visualized on a template brain surface. Top bottom: terms with highly correlated meta-analytic activation maps to the network topography.

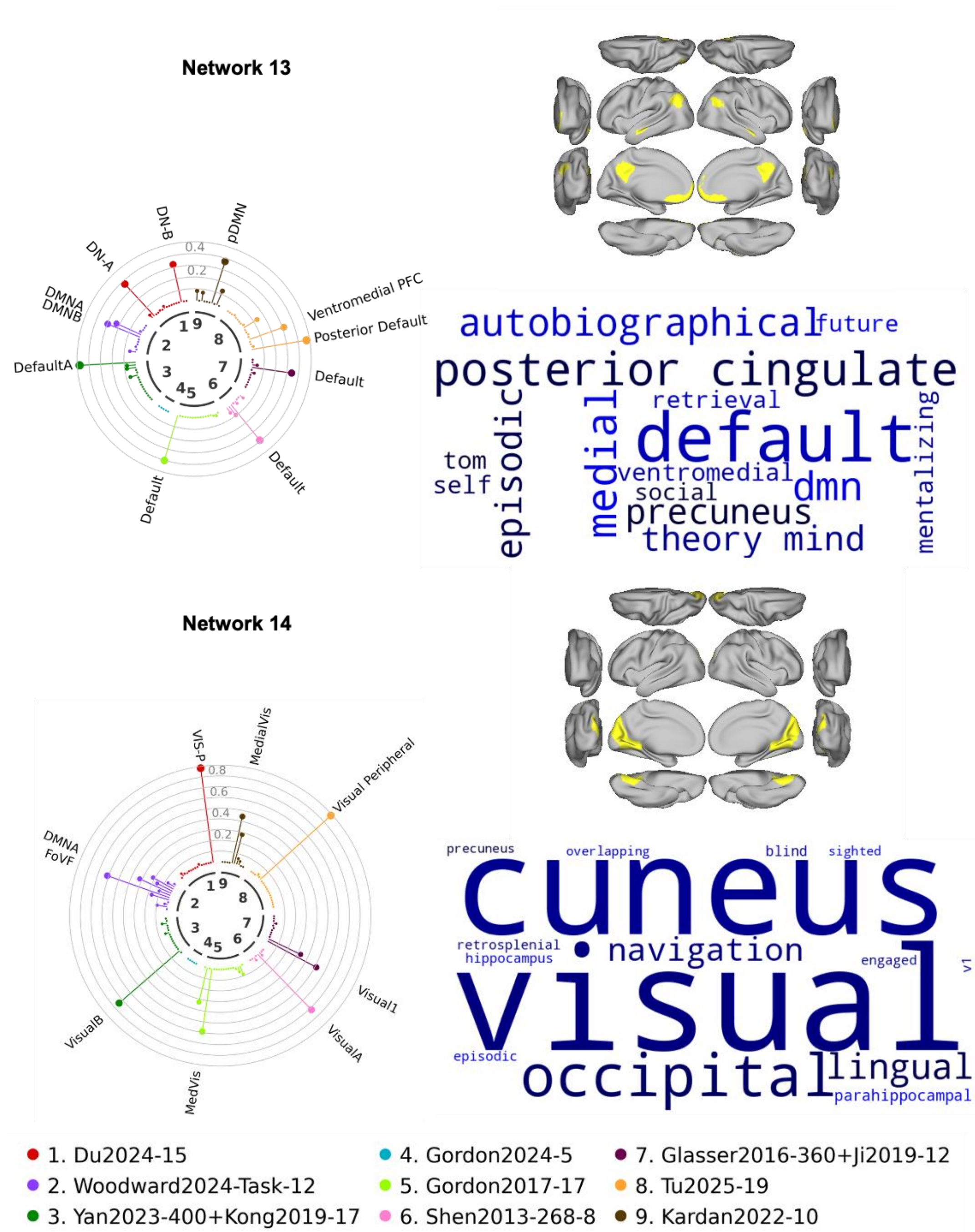

Supplementary Figure 11. *Correspondence between the 23 networks and previously reported functional networks/decoded terms (Networks 13-14)*. Left: Circular bar chart plot from the network correspondence toolbox for spatial correspondence with the network locations with >0.35 probability and 7 atlases from the literature. Circles illustrate the magnitude of the dice coefficient in intervals of 0.1. Only significant networks from the spatial permutation test ($p<0.05$) were named. Top right: the network locations with >0.35 probability visualized on a template brain surface. Top bottom: terms with highly correlated meta-analytic activation maps to the network topography.

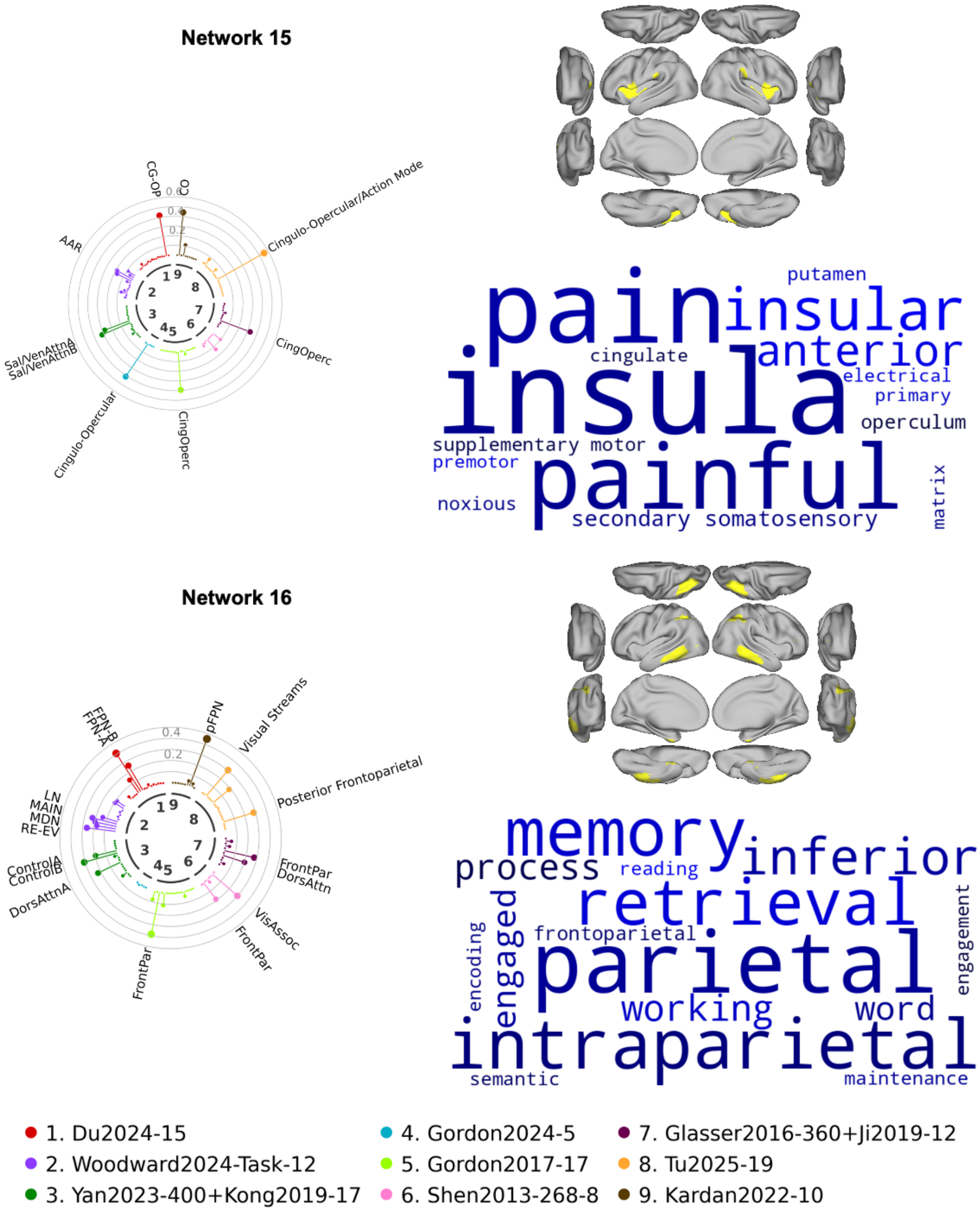

Supplementary Figure 12. *Correspondence between the 23 networks and previously reported functional networks/decoded terms (Networks 15-16)*. Left: Circular bar chart plot from the network correspondence toolbox for spatial correspondence with the network locations with >0.35 probability and 7 atlases from the literature. Circles illustrate the magnitude of the dice coefficient in intervals of 0.1. Only significant networks from the spatial permutation test (p<0.05) were named. Top right: the network locations with >0.35 probability visualized on a template brain surface. Top bottom: terms with highly correlated meta-analytic activation maps to the network topography.

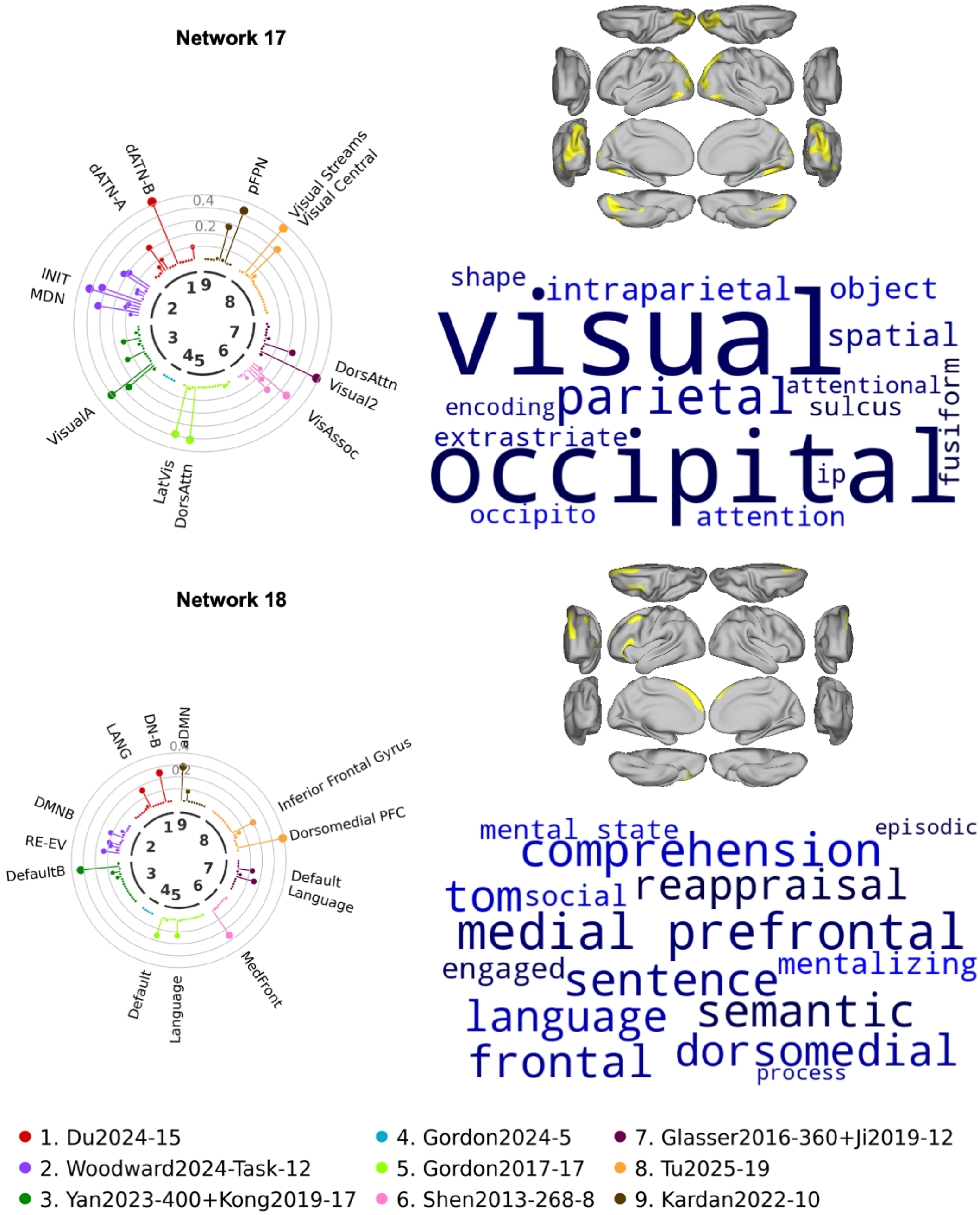

Supplementary Figure 13. *Correspondence between the 23 networks and previously reported functional networks/decoded terms (Networks 17-18)*. Left: Circular bar chart plot from the network correspondence toolbox for spatial correspondence with the network locations with >0.35 probability and 7 atlases from the literature. Circles illustrate the magnitude of the dice coefficient in intervals of 0.1. Only significant networks from the spatial permutation test ($p<0.05$) were named. Top right: the network locations with >0.35 probability visualized on a template brain surface. Top bottom: terms with highly correlated meta-analytic activation maps to the network topography.

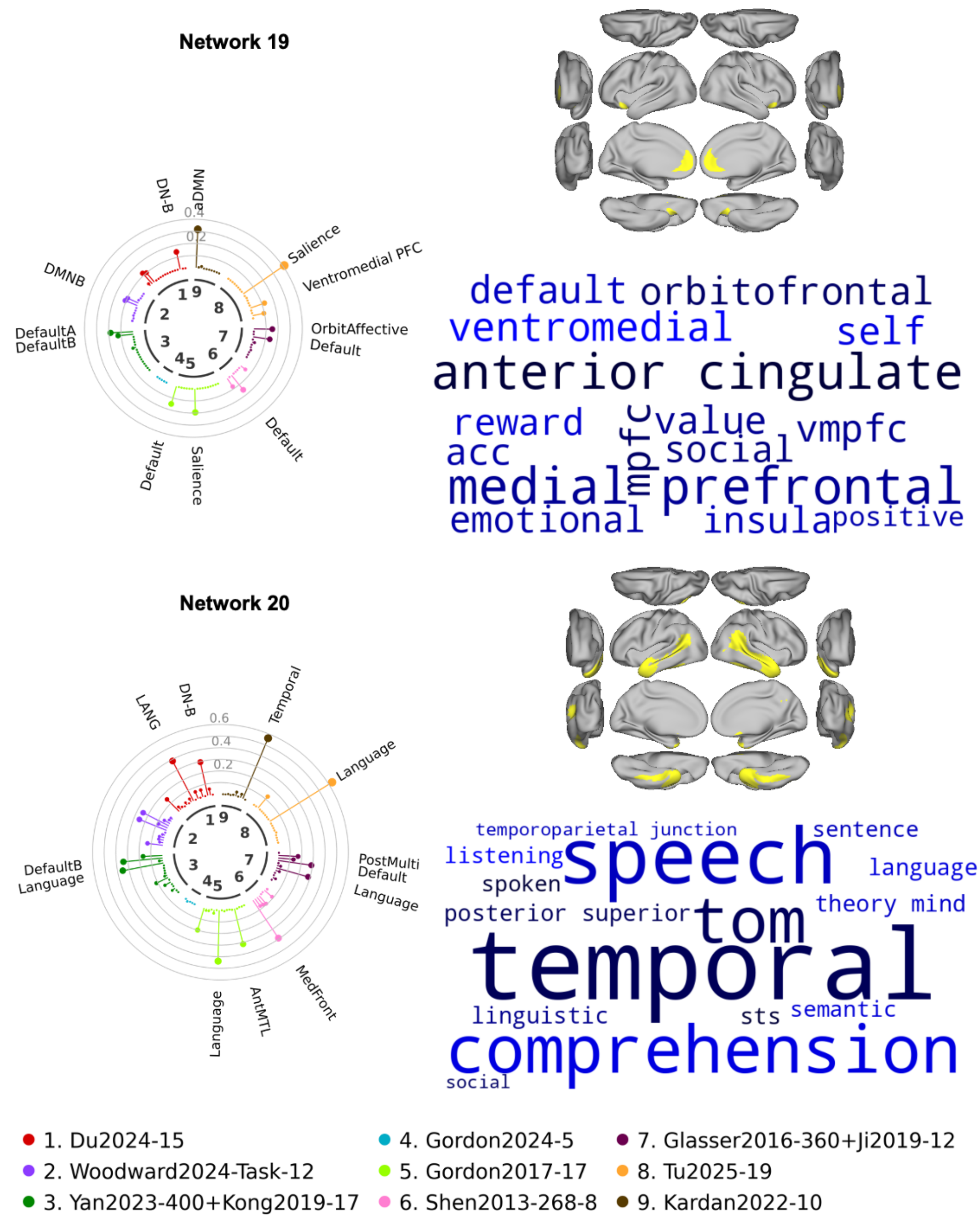

Supplementary Figure 14. *Correspondence between the 23 networks and previously reported functional networks/decoded terms (Networks 19-20)*. Left: Circular bar chart plot from the network correspondence toolbox for spatial correspondence with the network locations with >0.35 probability and 7 atlases from the literature. Circles illustrate the magnitude of the dice coefficient in intervals of 0.1. Only significant networks from the spatial permutation test ($p<0.05$) were named. Top right: the network locations with >0.35 probability visualized on a template brain surface. Top bottom: terms with highly correlated meta-analytic activation maps to the network topography.

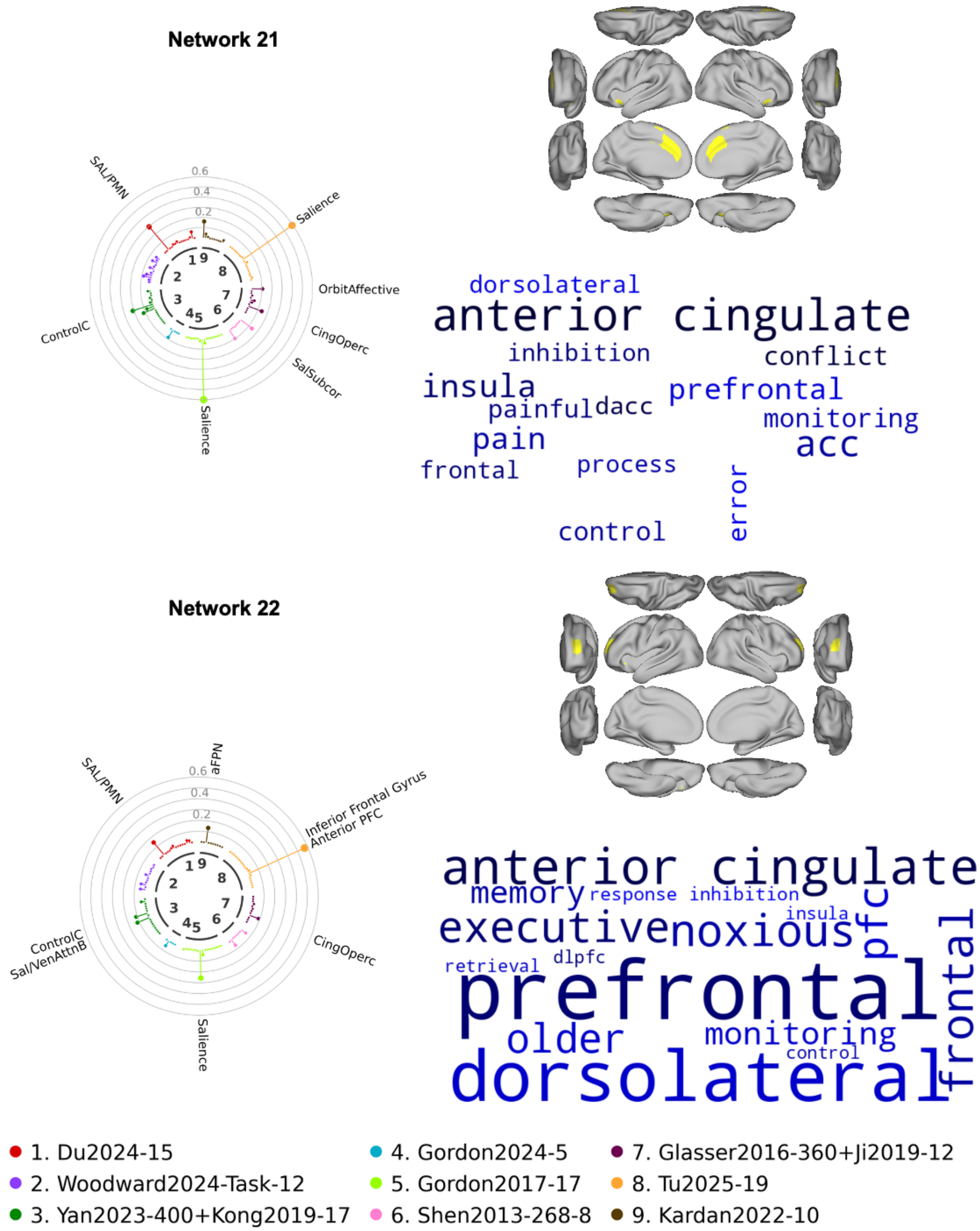

Supplementary Figure 15. *Correspondence between the 23 networks and previously reported functional networks/decoded terms (Networks 21-22)*. Left: Circular bar chart plot from the network correspondence toolbox for spatial correspondence with the network locations with >0.35 probability and 7 atlases from the literature. Circles illustrate the magnitude of the dice coefficient in intervals of 0.1. Only significant networks from the spatial permutation test (p<0.05) were named. Top right: the network locations with >0.35 probability visualized on a template brain surface. Top bottom: terms with highly correlated meta-analytic activation maps to the network topography.

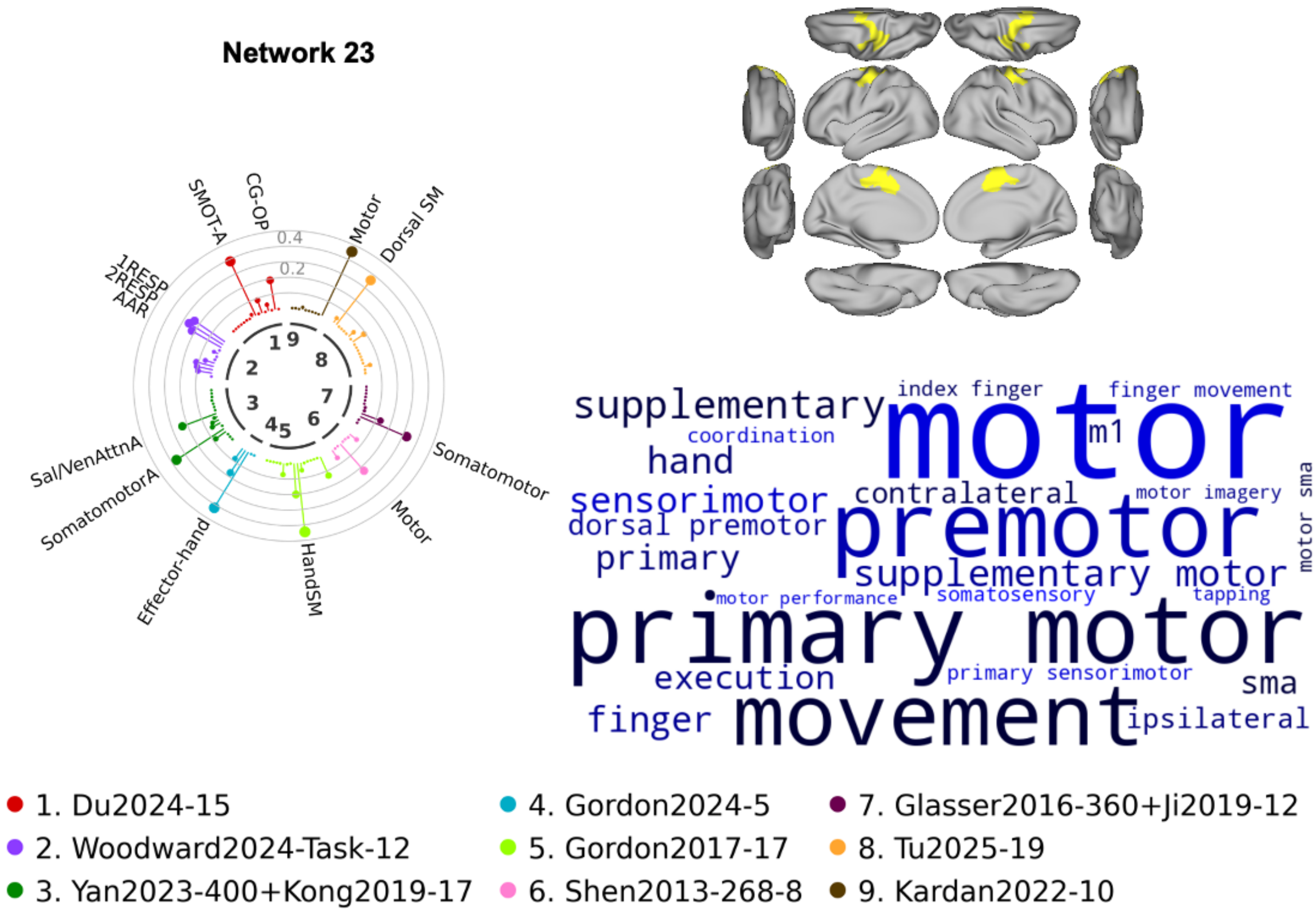

Supplementary Figure 16. *Correspondence between the 23 networks and previously reported functional networks/decoded terms (Networks 23)*. Left: Circular bar chart plot from the network correspondence toolbox for spatial correspondence with the network locations with >0.35 probability and 7 atlases from the literature. Circles illustrate the magnitude of the dice coefficient in intervals of 0.1. Only significant networks from the spatial permutation test ($p<0.05$) were named. Top right: the network locations with >0.35 probability visualized on a template brain surface. Top bottom: terms with highly correlated meta-analytic activation maps to the network topography.

Below are the modified main text edits that discuss this change:

"To help put the networks into context and relate them to potential behavioral significance… *Because task-based fMRI or multimodal data were not available in the present study, functional interpretations of the identified networks are necessarily indirect and based on spatial correspondence with prior reports rather than direct functional validation.*" and

"To further compare and group the 23 fine network divisions identified here with prior reports in adults…*The main reason these finely divided adult networks were chosen was that the spatial probability map and average FC in Lynch et al. 2024 was created from highly sampled precision*

fMRI data with a finer division than other group-based network identifications, allowing us to test whether our fine divisions correspond to prior adult literature or they represent children-specific networks."

For evaluation of the correctness of individualized network detection, it may not be a good practice to use two sample t tests on NMI indices. Statistical significance does not necessarily mean individual information preservation.

It is possible that the reviewer was referring to the concern that pooling all within-subject NMI values and all between-subject NMI values and comparing them using a two-sample t-test may not capture whether within-subject NMI is higher for all subjects. To address this, we additionally quantified the number of subjects for whom within-subject NMI exceeded between-subject NMI. This subject-level comparison more directly assesses whether individual-specific information is preserved relative to the group. We have updated the analysis accordingly and revised the relevant text as follows:

"The consistency of network assignment across the split halves (measured with normalized mutual information, NMI) was significantly higher (two-sample t-test $p<0.001$) within subject (Y2: NMI = 0.4822 ± 0.0669, Y3: NMI = 0.4919 ± 0.0686) than between subjects (Y2: NMI = 0.3274 ± 0.0383, Y3: NMI = 0.3250 ± 0.0379) (Figure 3A, 3C-F), *with NMI higher within subject in almost all subjects (Y2: 49, Y3: 47 out of 49)",*

and

"The consistency of network assignment across the two longitudinal sessions was significantly higher (two-sample t-test $p<0.001$) within subject (NMI = 0.5190 ± 0.0444) than between subjects (NMI = 0.3788 ± 0.0330) (Figure 3B, G-H), *and the NMI was higher within subject for all 49 subjects*, indicating the individual-specific networks were stable from 2 to 3 years old."

Alternatively, the reviewer may be raising a more fundamental concern regarding the absence of a ground-truth reference for validating individualized networks. In the absence of additional information such as task activation, the best we can do is to demonstrate that the resulting networks capture reliable, subject-specific variability beyond the population average. The analyses above directly address this point by showing robust within-subject consistency and clear individual specificity. Please also see the response to an earlier point for our attempts to further validate the networks in the absence of ground truth.

Abstract: What is the exact sample size? N should be specific (not as >300)

We have now updated the sample size in the abstract to 328, which is the sum from two datasets in Table 2.

Fig. 6 show correlations between imaging results and behavioral scores but these correlations are marginally significant, or with a small effect size (r value), and may not survive multiple comparison corrections.

The reviewer's observation was correct about significance for the Default-Anterolateral network, and we have dedicated a paragraph in the discussion section for that. As for the effect size, a small effect size of 0.1 to 0.2 is not uncommon for neuroimaging studies (Marek et al. 2022 Nature). Figure 6 and the relevant text were substantially revised to provide better statistical details and choices, and a random effect of the subject was incorporated. Please see response to Reviewer 3.

Reviewer #2 (Remarks to the Author):

In this manuscript entitled "Commonality and Variability in Functional Networks in Children Under 5 Years Old", Tu et al. investigated the individualized large-scale functional brain networks during early childhood. The authors characterize person-specific network organization and its developmental stability across two independent cohorts of toddlers, providing valuable data on early functional brain architecture. The finding that individualized and behaviorally relevant network patterns, including early language-network lateralization, appear before the age of five offers important insights into the maturation of functional organization in the developing brain. Overall, this is a well-designed study that makes a meaningful contribution to our understanding of early functional network development.
I have only a few minor comments that the authors may consider addressing to further improve clarity.

1. It would be helpful for the authors to further clarify the rationale for selecting K = 23 as the optimal number of networks. According to Supplementary Figure 3, K = 8 also appears to represent a local peak. Moreover, the study defines 23 functional networks in the developing brain, yet large-scale functional organization is still maturing during early childhood. It would therefore be important to discuss how stable these fine-grained network subdivisions are across individuals and sessions, and whether this level of parcellation is biologically meaningful and justified for this age range.

We thank the reviewer for raising this important point regarding the choice of K and the biological interpretability of fine-grained network subdivisions in early childhood. According to Supplementary Figure 3, we do not observe evidence that K = 8 constitutes a distinct local stability peak; rather, K = 8 yields a solution similar to commonly used coarse parcellations (e.g., K = 7). As described in the Methods, we selected K = 23 as the largest value of K prior to a decline in stability, thereby balancing reproducibility with sensitivity to finer-scale functional organization.

Specifically, the number of clusters (K) was determined using a split-half stability analysis in which the 81 sessions were randomly divided into two halves 20 times. For each split, parcel-wise functional connectivity profiles were concatenated and clustered using k-means with K

ranging from 1 to 40. Cluster stability was quantified using normalized mutual information (NMI) between cluster assignments predicted across split halves. A local maximum in stability was observed at K = 23, which was selected for all subsequent analyses. This choice was motivated by the goal of examining fine-level network organization while maximizing sensitivity to connectivity patterns that may be consistently expressed in only a subset of individuals. The Methods section related to Figure 1 has also been heavily revised for clarity, please see response to reviewer 3.

Importantly, in our framework, K reflects the number of stable functional connectivity patterns present across the cohort, rather than the number of networks that must be present in every individual or at all developmental stages. This distinction is particularly relevant during early childhood, when large-scale functional organization is still maturing and certain patterns may be transient, heterogeneous, or subgroup-specific. By clustering concatenated individual functional profiles rather than group-averaged data, our approach retains such patterns if they recur reliably across individuals, even if they are not ubiquitous.

Conceptually, in a developmental scenario where two distinct patterns (A and B) are expressed earlier and later converge into a single pattern (C), group averaging could yield only a C-like prior. In contrast, clustering concatenated individual profiles allows A-, B-, and C-like patterns to be retained as distinct clusters, provided each pattern is sufficiently represented. While we did not observe such extreme transitions in the present age range, this consideration motivates the use of a higher yet stable K to capture developmental heterogeneity.

Regarding stability and biological relevance, I believe the reviewer wanted to know whether these networks 1) are present in all individuals and all ages, 2) whether they match the underlying structural and cellular processes. For the first point, as explained above, we did not start with the assumption that all 23 networks had to be present for all individuals and all ages, but it was true for these two cohorts. For the second point, although we did not have diffusion imaging data or tissue analysis to directly validate the biological relevance, we provided extensive comparison with prior literature (e.g., Table 1, Supplementary Figures 5-16, Supplementary Figures 18-19). The close correspondence between many of the identified network topographies and those reported in independent adult and pediatric studies supports the biological plausibility of these fine-grained networks. Notably, the breadth of our efforts to validate the biological plausibility of the identified networks exceeded that reported in most prior network identification studies in pediatric populations. However, direct validation of evidence from other kinds of biological data, such diffusion tensor imaging and cytoarchitecture would be helpful to assess the biological relevance of the identified networks. We have added this limitation to our discussion to read as:
“Direct evidence from diffusion tensor imaging and cytoarchitecture would also be useful in validating the biological relevance of the identified networks.”

2. Regarding the results reported in lines 344–345, when assessing the associations between children’s verbal and nonverbal skills and network lateralization, it would be helpful to clarify whether permutation testing was performed to validate the statistical significance (e.g., by

shuffling the correspondence between children's verbal and nonverbal skills and network measures).

We thank the reviewer for this suggestion. However, the permutation test is non-trivial in this case because there are multiple scans for some subjects. Therefore, we adopted a permutation strategy that preserves age effects and within-subject dependence, and added this analysis to the Supplementary Materials:

"Non-parametric permutation test for brain-behavioral relationship

To validate the statistical significance of associations between functional network laterality and behavioral measures, we performed nonparametric permutation testing in addition to the parametric linear mixed-effects analyses reported in the main text. Because behavioral measures varied across scan sessions and some children contributed multiple scans, permutation testing was implemented using a subject-level residual sign-flipping procedure derived from a reduced mixed-effects model (Winkler et al., 2014). Specifically, we first fit a reduced model including age as a fixed effect and a random intercept for subject (LI ~ age + (1 | subject)) to obtain fitted values and residuals under the null hypothesis of no behavioral effect. Residuals were then randomly sign-flipped at the subject level, preserving the within-subject dependence structure and the effects of age. For each permutation, a permuted outcome was reconstructed by adding the sign-flipped residuals to the fitted values, and the full mixed-effects model including the behavioral predictor was refit. Repeating this procedure across 1000 permutations yielded a null distribution of the behavioral regression coefficient, against which the observed coefficient was compared to obtain an empirical two-sided permutation p-value (minimum 0.001).

Permutation testing confirmed that the observed association between Language network laterality and VDQ remained statistically significant (p_perm = 0.012), consistent with the parametric mixed-effects result reported in the main text. In contrast, associations involving NVDQ did not reach significance under permutation testing (p_perm = 0.150), in line with parametric estimates. Default Anterolateral network and VDQ (p_perm = 0.116) or NVDQ (p_perm = 0.533) associations were also not significant. Together, these results indicate that the reported language-specific laterality effects are robust to distributional assumptions and are not driven by violations of model assumptions or repeated-measures structure."

3. In line 500, the reported number of participants (n = 341) appears to be inconsistent with Table 2 (150 + 178 = 328). Please check and clarify this discrepancy.

Thank you for catching this error; that line has now been corrected. There were some older BCP subjects who had watched movies instead of sleeping and they had initially been counted when this table was created. However, given possible variation due to state differences, participants watching movies were excluded from the manuscript.

4. It would be helpful to include information on the age distribution of participants from each dataset in Table 2.

Supplementary Figures 2 and 21 plot the age distribution of participants from each dataset. This has now been clarified in the text of the methods section just before Table 2, which now reads: *"Age and sex distributions are provided in Supplementary Figures 2 and 21 "*

5. In line 586, please specify which infant MNI template was used and provide the corresponding reference.

The infant-specific MNI template reference was added and the original paragraph was revised to read as:
"**MRI data preprocessing.** Data processing used the DCAN-Labs infant-abcd-bids-pipeline (v0.0.22), which largely follows the HCP processing (Glasser et al., 2013) and the steps described previously for the ABCD dataset (Feczko et al., 2021). *The processing steps were similar to the adult data except for the use of infant-specific MNI templates at ages 08-11/11-14/14-17/17-21/21-27/27-33/33-44/44-60 months (Fonov et al., 2009) to better register the structural data*."

Reviewer #3 (Remarks to the Author):

In this study, the authors highlight the importance of individualized brain network extraction in developmental cognitive neuroscience and propose a novel framework for identifying subject-specific functional networks from single resting-state fMRI scans. Their findings suggest that several large-scale brain networks—and their hemispheric lateralization—emerge before the age of five. Notably, they report that left-lateralization within language-related networks is associated with cognitive abilities such as verbal performance. Overall, the manuscript presents an interesting concept with potentially meaningful implications for understanding early brain functional organization. However, the paper exhibits notable limitations in structure, clarity, and methodological transparency, particularly concerning the presentation and interpretation of methods and results. Major concerns include an overly broad and methodologically diffuse ("kitchen-sink") analytical approach, ambiguously defined network "templates," insufficient justification for evaluation metrics, and an unclear rationale for dataset selection. The following points outline key issues and recommendations for improvement.

1. Insufficient Dataset Description and Selection Criteria
The manuscript lacks a clear and consistent description of the datasets utilized. The reported sample sizes in the text do not align with those presented in the tables, and no explanation is provided for these discrepancies. The study appears to involve two independent datasets, yet their respective roles and comparative purposes are not clearly articulated. For instance, the authors mention using a subset of the first dataset to build the network template, but it remains unclear how the remaining data were incorporated into subsequent analyses. Moreover, if the study does not aim to examine pathological or atypical populations, the inclusion of certain datasets requires further justification.

For the discrepancy between text and tables, please see response to Reviewer 2 comment 3.

We have updated the rationale for choosing the two datasets:
"We used two high-quality infant/toddler datasets spanning approximately 1-5 years (total number of participants = 328) *sampling distinct socioeconomic backgrounds* for the current study *to ensure robustness of the findings*."

Preterm birth occurs in 1 in 10 women in the United States and is more frequent in populations with an adverse socioeconomic background. Since we wanted to sample a diverse demographic for our results, and for increasing statistical power, we did not feel there was a strong need to exclude the preterm-born subjects, if that is what the reviewer was referring to as "pathological or atypical populations". We have updated the relevant sentence to read as:

"A subset of 81 sessions (40 from Y2 and 41 from Y3) from healthy term-born toddlers had more than 20 min of usable low-motion data were used to generate the network templates, *and vertexwise assignments of individualized networks were created for all sessions. Since we wanted to sample a diverse demographic for our results, and for increasing statistical power, we did not exclude the 20 preterm-born subjects*.

2. Clarity of Abstract and Introduction
The abstract is underdeveloped and omits several key elements. For example, it does not explicitly mention the development of functional reference templates or report statistical significance levels. Additionally, some statements in both the abstract and introduction are inaccurate or overstated. The concept of individualized network extraction is not novel per se; numerous approaches—including ICA, PCA, and other data-driven frameworks—can achieve similar objectives. Prior research has also delineated individualized brain networks in children and infants, including hemispheric lateralization patterns, and several age-specific brain templates have been established in earlier work. The authors should more clearly delineate how their approach advances beyond existing methodologies or provides distinct contributions to the field.

We appreciate the reviewer's comment and agree that individualized network estimation per se is not novel, as approaches such as ICA, PCA, and template-based dual regression have been widely used in both adult and developmental populations. We have revised the abstract and Introduction to clarify that the primary contribution of our work is not the introduction of individualized network extraction, but rather the characterization of early functional organization using a shared, age-appropriate functional network reference that preserves individual-specific and subgroup-specific patterns.

Specifically, many existing age-specific networks are derived from group-averaged data, which can attenuate inter-individual variability and suppress network patterns that are not uniformly expressed across children during early development. Our approach uses clustering on concatenated individual functional connectivity profiles to identify stable functional patterns present across the cohort, followed by template-based individual network mapping to ensure cross-subject correspondence.

We have revised the abstract to emphasize these points and to better delineate how our work complements and extends prior developmental studies by providing a finer-grained, developmentally appropriate network characterization linked to longitudinal stability and behavioral measures.

“Large-scale functional brain networks undergo rapid reorganization during early childhood, yet most existing age-specific networks are derived from group-averaged data, which can obscure both inter-individual variability and network patterns that are not uniformly expressed across children during this highly dynamic period. This limitation is particularly pronounced in association cortices, where functional organization emerges later and is more heterogeneous across individuals. Here, we characterized early functional network organization while preserving individual-specific features within a shared, age-appropriate functional network reference. Using resting-state fMRI data from two independent cohorts of children aged 8–60 months (total N = 328), we identified 23 reproducible large-scale functional connectivity patterns via data concatenation across individuals, capturing shared developmental structure without suppressing less ubiquitous patterns. We then applied template-based individual network mapping to delineate each child’s network organization, enabling direct comparison across individuals and developmental stages. This fine-grained division extends prior developmental studies that have largely focused on coarser network representations.

We find that stable, individualized network organization is evident well before age 5. Sensory networks showed greater cross-subject consistency than association networks, consistent with known maturational gradients. Person-specific network configurations exhibited moderate longitudinal stability over 2–3 years (normalized mutual information ≈ 0.52). Notably, emerging left-lateralization of language-related networks during early childhood was significantly associated with age-normalized verbal ability, linking functional network organization to developing cognitive skills. Together, these results situate individual variability within a shared developmental reference and provide a detailed framework for studying typical brain development and early markers of atypical trajectories.”

3. Presentation of Results
The results section lacks clear organization and coherence, making it difficult for readers to follow the progression of analyses. Critical methodological details—such as the parcellation scheme and the mapping between functional connectivity measures and brain networks—are insufficiently described or introduced too late. As a result, interpretation of the results is substantially impeded. Improved figure organization and clearer linkage between text and visualizations would greatly enhance comprehensibility.
Please see the response to comment 4 below, which addresses both comment 3 and comment 4.

4. Presentation of the Methods Section
The methods section is difficult to follow and would benefit from substantial reorganization and clarification. The authors are encouraged to move detailed preprocessing steps to the Supplementary Information and summarize the key procedures more concisely in the main text.

The sudden introduction of numerical quantities (e.g., “59,412”) without context is confusing. The transition from preprocessing to the construction of the functional connectome lacks clear definitions of critical terms, such as “vertex,” “parcel,” and “vertex-to-parcel connectome.” A schematic flowchart could help guide readers through the analytical pipeline. Moreover, the description of the individual-specific network derivation is unclear. After introducing the network template, the term “seed FC” is ambiguously defined—does this refer to parcel-wise functional connectivity within each scan? The statement “we identified the network template with … to the seed FC” is particularly opaque. It remains unclear how the “individual functional networks” are ultimately derived from the template.

We thank the reviewer for the constructive feedback. We agree that clearer orientation within the Results and Methods section is important, particularly given the methods-last format of the manuscript.

To address this concern, we have revised the first Results section (“Identification of age-appropriate functional connectivity patterns using concatenated individual data”), as well as the relevant parts in the Methods section to more explicitly guide the reader through the analysis pipeline. We also merged Figure 1 and the original Supplementary Figure 5 to provide a clean schematic flowchart. In particular, we now explicitly define parcels, vertices, parcel-wise FC profiles, and vertex-to-parcel connectivity clearly distinguish two conceptual stages of the analysis:

1. Identification of age-appropriate functional connectivity patterns using parcel-wise FC profiles concatenated across individuals, and
2. Delineation of individual-specific, fine-grained network topography by mapping these parcel-level patterns back to the cortical surface at the vertex level via template matching.

We added brief orienting language at the point where the analysis transitions from parcel-level clustering to vertex-level network assignment, clarifying the data representation, rationale for this transition, and how each step contributes to subsequent results. Detailed methodological descriptions (e.g., parcellation scheme, FC construction, clustering and template-matching procedures) remain in the Materials and Methods section. The new relevant results section reads:

“*Identification of age-appropriate functional connectivity patterns using concatenated individual data*

Previously, we identified functional networks based in 2-year-old children using group-averaged functional connectivity (FC) computed within an age-specific area parcellation (Tu et al., 2025a). While this approach captures dominant population-level organization, anatomically matched cortical areas can exhibit distinct FC profiles across individuals during early development. As a result, averaging FC across participants may obscure both inter-individual variability and connectivity patterns that are consistently expressed only in subsets of children (Supplementary

Figure 1; see Supplementary Text). To mitigate this limitation, we identified age-appropriate functional connectivity patterns by clustering concatenated individual FC profiles, rather than group-averaged connectivity. Using the same area parcellation as in prior work (Tu et al., 2025a), FC was computed between all pairs of parcels within each session, yielding parcel-wise FC profiles in which each parcel served both as a feature (connectivity target) and as a sample, pooled across individuals. Concatenating these parcel-wise FC profiles across participants allows reproducible connectivity patterns to emerge without requiring them to be present in every individual. Using this approach, we clustered FC profiles from 81 fMRI sessions (72 unique children aged 2–3 years, including 9 children with longitudinal data at Y2 and Y3; all sessions >20 minutes of low-motion data; eLABE Dataset 1; Supplementary Figure 2), analogous to prior literature (Yeo et al., 2011; Zhu et al., 2025). A split-half stability analysis revealed a local peak at K = 23 clusters (Supplementary Figure 3). Full methodological details are provided in Materials and Methods.

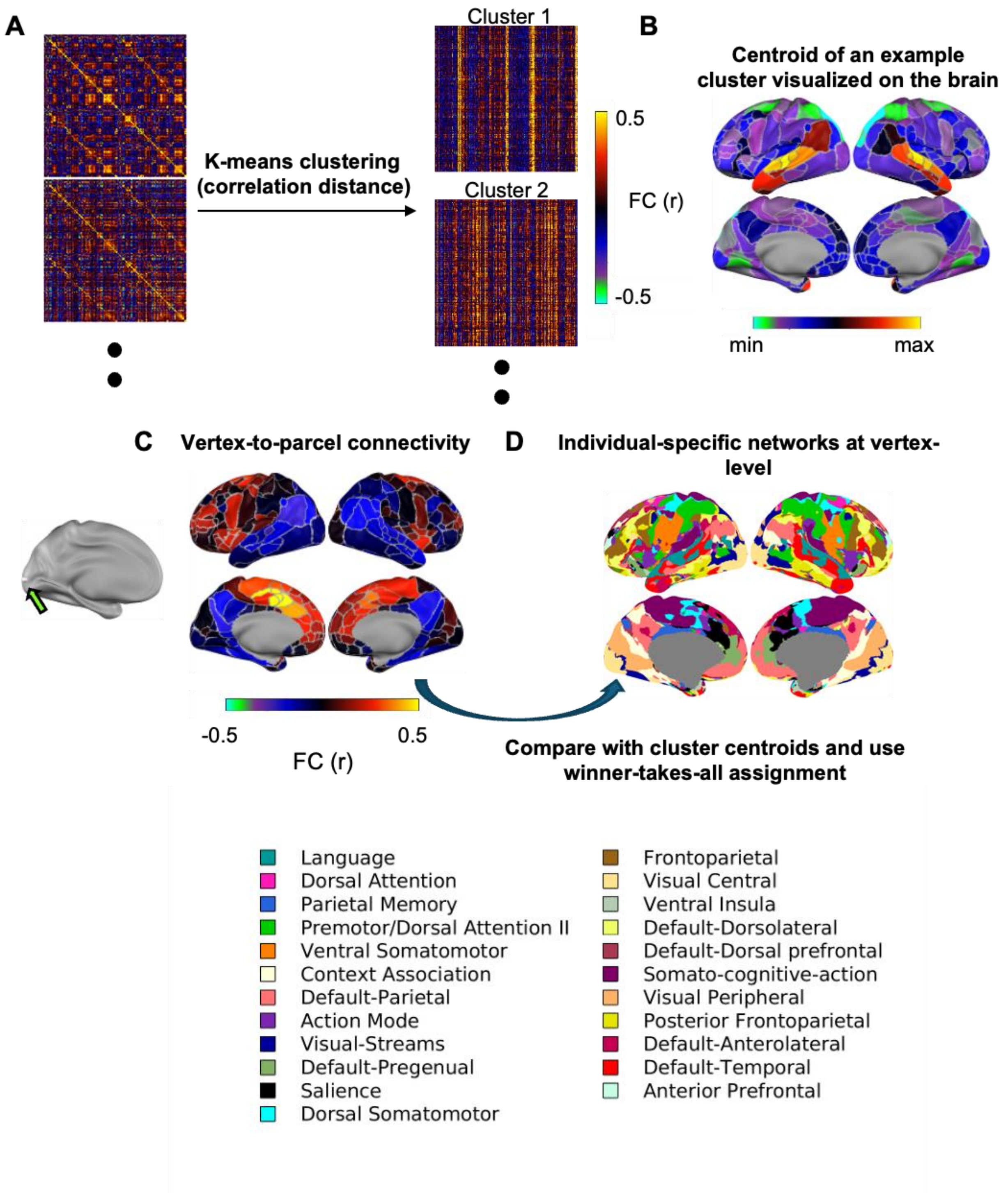

Figure 1. Creation of group-level reference template and individual-specific network mapping. A) Example FC matrices being concatenated and clustered into internally similar clusters of FC profiles. B) Example template FC profiles (centroids) for the Language network. C) Vertex-to-parcel connectivity. D) Individual-specific networks at vertex-level. Network names and colors were chosen based on anatomy and prior literature.

Individual-specific network mapping

The resulting 23 resting-state network profiles represent reproducible functional connectivity patterns observed across subjects at 2 to 3 years. The cluster centroids therefore serve as age-appropriate reference FC profiles or "network templates" (example in Figure 1F;Supplementary Figure 4). These reference profiles were subsequently used to delineate individual-specific network organization at finer spatial resolution. Specifically, vertex-level FC profiles (vertex-to-parcel connectivity maps, Figure 1C) were compared with the 23 reference profiles using Pearson's correlation, and each vertex was assigned to the most similar network using a winner-take-all procedure, following prior work (Gordon et al., 2017a). This step enables detailed vertex-wise network mapping (Figure 1D) while maintaining correspondence with the age-appropriate connectivity patterns identified above. "

And we updated the relevant methods sections to be:

"Functional connectivity representations
Analyses were conducted using an age-specific cortical parcellation previously demonstrated to fit this developmental period (Tu et al., 2025a). Parcels refer to anatomically defined cortical regions from this parcellation. Vertices refer to surface mesh points on the cortical surface. Two types of functional connectivity (FC) representations were used, each serving a distinct analytical role. Parcel-to-parcel FC was used to identify reproducible connectivity patterns across individuals. Vertex-to-parcel FC was used to assign network identities to individual cortical vertices based on data-driven network templates. In both cases, FC was defined as the Pearson correlation between BOLD time series and computed across the neocortex in both hemispheres.

Identification of age-appropriate functional connectivity patterns
For each fMRI session, a parcel-by-parcel FC matrix was computed. Each parcel's connectivity profile to all other parcels (i.e., one row of the parcel-to-parcel FC matrix) was treated as a single parcel-wise FC profile. In this representation, parcels serve both as samples (rows) and as features (connectivity targets). Parcel-wise FC profiles from all sessions were concatenated across individuals prior to clustering. The analysis included 81 fMRI sessions from 72 unique children aged 2–3 years (9 children contributed longitudinal data at Y2 and Y3; all sessions contained >20 minutes of low-motion data; eLABE Dataset 1). Clustering was performed using K-means with correlation distance. The resulting cluster centroids represent reproducible parcel-wise FC patterns and were used as age-appropriate reference network profiles. Initial centroid position was determined by the K-means++ algorithm (default). Because the K-means solution is susceptible to the initial centroid location, we set the number of times to repeat the clustering to 1000 to minimize the possibility of stopping at a local minimum. The output K cluster centroids were subsequently used as network templates for template matching.

Selection of the number of clusters
The number of clusters (K) was selected using a split-half stability analysis. The 81 sessions were randomly divided into two halves 20 times. For each split, parcel-wise FC profiles were

concatenated separately and clustered using k-means with K ranging from 1 to 40. Cluster stability was quantified as the normalized mutual information (NMI) between cluster assignments predicted across split halves. A local maximum in stability was observed at K = 23, which was selected for all subsequent analyses. This choice favors sensitivity to finer-grained connectivity patterns, including patterns that may be consistently expressed in only a subset of individuals.

Individual-specific network mapping

To derive individual-specific network organization at finer spatial resolution, reference network profiles were mapped onto the cortical surface at the vertex level. For each session, vertex-to-parcel FC was computed by correlating the BOLD time series of each cortical vertex with the mean BOLD time series of each parcel, yielding a vertex-wise FC profile for every vertex. Each vertex-wise FC profile was compared with the parcel-level reference network profiles using Pearson's correlation. Vertices were assigned to the network with the highest correlation using a winner-take-all procedure, similar to prior studies (Gordon et al., 2017). No magnitude thresholding or distance-based exclusion was applied to the FC profiles. We retained all connections, including weak and anticorrelated values, as these may contribute to network differentiation, particularly in early development."

Additionally, we reorganized the mention of "59412" for better context: "We then focused on only the cortical vertices on the fs_LR-32k surface *(59412 after excluding medial masks)* for the subsequent analyses."

We believe these revisions improve the logical flow and interpretability of the Results section without altering the analyses or conclusions.

5. Statistical Analysis

The statistical framework is insufficiently described. It is unclear how associations were modeled or whether random effects were included to account for repeated scans within subjects. The manuscript does not specify whether outlier detection or exclusion procedures were applied (as seen clear outliers in the figures), nor does it indicate whether multiple comparison corrections were implemented. Furthermore, the choice of covariates in the regression models is not discussed. These omissions raise concerns regarding the robustness and interpretability of the reported findings.

We have revised Figure 7, Supplementary Figure 25 and relevant sections that analyzed the relationship between network laterality and age/language ability to use a linear mixed model with a random intercept in subject, instead of Pearson's correlation. Age was included as a covariate in all models due to its strong developmental relevance for both network lateralization and behavioral measures. Mean framewise displacement was included in secondary analyses to assess robustness to head motion. No additional covariates were included to avoid overfitting and because other demographic variables were not hypothesized to confound the specific associations tested. This modification created complexity and complicated interpretation on mediation, so we removed the mediation analysis from the text. Now the results reads:

" To quantify network lateralization, we calculated a "laterality index" (LI) defined as the difference in the network surface area between the left and right hemispheres, expressed as a percentage of the total network surface area. In children at 8–60 months (Dataset 2), the Language network showed weak left-lateralization (LI = 0.07 ± 0.13; one-sample t-test, $t(300) = 9.2858$, $p < 0.001$), whereas the Default-Anterolateral network showed strong left-lateralization (LI = 0.40 ± 0.19; $t(300) = 37.1876$, $p < 0.001$). Using linear mixed-effects models with a random intercept for subject, laterality of the Language network increased with age ($\beta = 0.025$ per year, SE = 0.011, $t = 2.29$, df = 299, $p = 0.023$, Figure 6A). Laterality of the Default-Anterolateral network also increased with age ($\beta = 0.059$ per year, SE = 0.015, $t = 3.82$, df = 299, $p < 0.001$, Figure 6B).

Associations between network laterality and behavioral measures were assessed using linear mixed-effects models including age and behavioral scores as fixed effects and a random intercept for subject. Verbal and non-verbal abilities were quantified using the Mullen Verbal Developmental Quotient and Non-verbal Developmental Quotient (VDQ and NVDQ; age-normalized scores from the Mullen Scales of Early Learning (Mullen, 1995)), excluding sessions without available scores (21 without VDQ and 19 without NVDQ). Language network laterality was positively associated with verbal ability beyond the effect of age ($\beta = 0.00122$ per VDQ point, SE = 0.00044, $t = 2.79$, df = 271.6, $p = 0.0057$, Figure 6C). In contrast, laterality of the Default-Anterolateral network did not significantly associate with VDQ ($\beta = 0.00107$ per VDQ point, SE = 0.00062, $t = 1.72$, df = 269.1, $p = 0.087$, Figure 6D). As a control analysis, Language network laterality did not significantly associate with NVDQ ($\beta = 0.00090$ per NVDQ point, SE = 0.00049, $t = 1.85$, df = 254.0, $p = 0.066$, Figure 6E), nor did the Default-Anterolateral network ($\beta = 0.00055$ per NVDQ point, SE = 0.00069, $t = 0.79$, df = 247.2, $p = 0.430$, Figure 6F). Associations in the same direction were observed in the longitudinal dataset (Dataset 1), but statistical significance was reduced potentially due to a smaller sample size, a more restricted age range centered around 2–3 years, and the use of the Bayley Scales of Infant and Toddler Development language composite as a proxy for verbal ability (Supplementary Materials, Supplementary Figure 25).

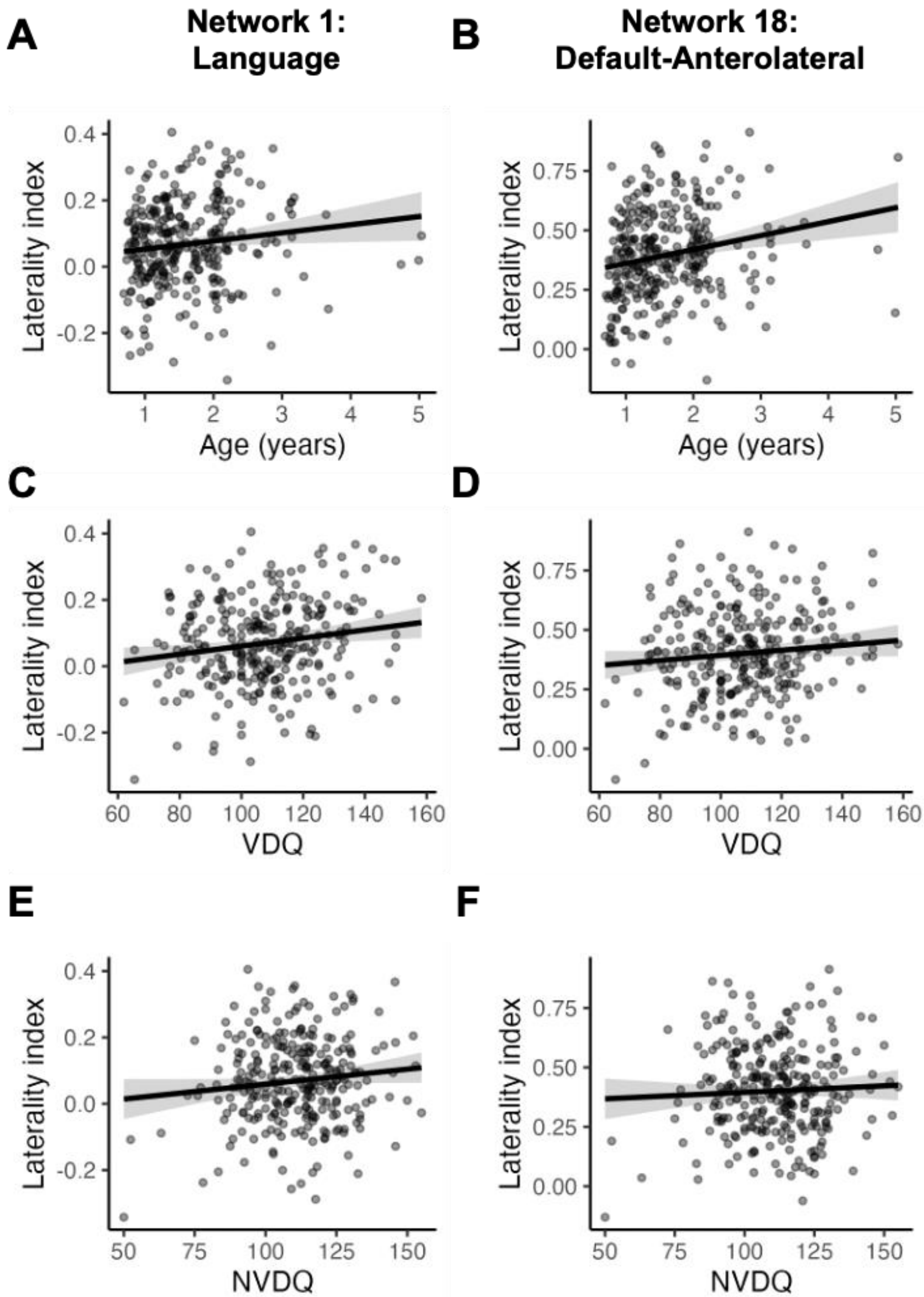
A
Network 1:
Language
B
Network 18:
Default-Anterolateral
Laterality index
0.4
0.2
0.0
-0.2
Age (years)
1
2
3
4
5
0.75
0.50
0.25
0.00
C
D
VDQ
60
80
100
120
140
160
E
F
NVDQ
50
75
100
125
150

***Figure 7**. The relationship between functional network laterality and age or Mullen Scales of Early Learning developmental quotients. Laterality index against A-B) age, C-D) verbal developmental quotient (VDQ), missing 21 scores, E-F) non-verbal developmental quotient (NVDQ), missing 19 scores. Shaded band = 95% CI of fixed effect (age was held constant in C-F).*

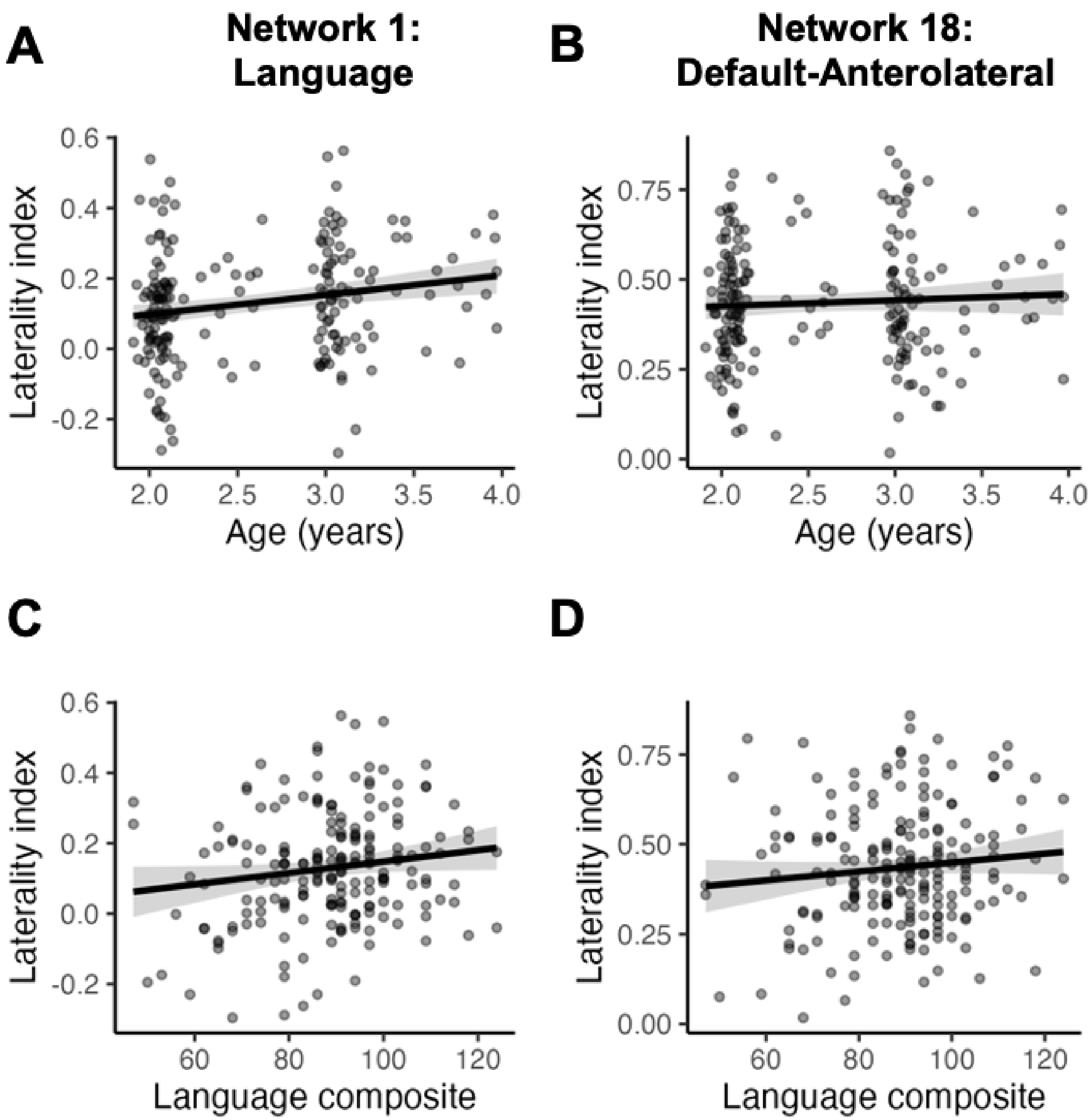

***Supplementary Figure 25**. The relationship between functional network laterality and age or Bayley Scales of Infant and Toddler Development age-normalized composite scores. A-B) Age, C-D) Language composite scores. Shaded band = 95% CI of fixed effect (age was held constant in C-D).*

In Methods:

"Statistics and Reproducibility
Associations between network laterality and age or behavioral measures were assessed using linear mixed-effects models implemented in R (lme4), with fixed effects for predictors of interest and a random intercept for subjects to account for repeated scans. Age was included as a covariate in all models due to its developmental relevance; mean framewise displacement was included in secondary analyses to assess robustness to motion. Model diagnostics of "outliers" included inspection of standardized residuals and subject-level influence using Cook's distance (Supplementary materials - "outlier analysis"); no outcome-based exclusions were applied. Analyses were hypothesis-driven and focused on predefined language-related networks and measures, and p-values are therefore reported without correction for multiple comparisons. The analysis code has been made publicly available to ensure reproducibility of the results: https://github.com/cindyhfls/Tu-2026-IndividualBabyNetworks."

And in Supplementary Materials:
"Outlier analysis
To assess whether associations between network laterality and age or behavioral measures were driven by extreme observations or influential participants, we conducted residual and influence diagnostics for all linear mixed-effects models. Specifically, we computed the proportion of observations with standardized residuals exceeding |3| and calculated subject-level Cook's distance, which quantifies the influence of each participant on fixed-effect estimates. For each association, the proportion of extreme residuals and the maximum Cook's distance are reported in Supplementary Table 2. Across all analyses, the proportion of observations exceeding |3| standardized residuals was small (≤0.3%), and Cook's distance values were modest, with no participant exerting disproportionate influence on model estimates. Accordingly, no outcome-based exclusions were applied.

Supplementary Table 2. Outlier analysis

Dependent variable: Laterality of Network 1 ("Language"), Dataset 2			
Independent variable of interest	*Outlier proportion*	*Max. Cook's distance*	*Corresponding figure*
age	*0.003*	*0.064*	*Figure 7A*
VDQ	*0*	*0.102*	*Figure 7C*
NVDQ	*0*	*0.200*	*Figure 7E*
Dependent variable: Laterality of Network 18 ("Default-Anterolateral"), Dataset 2			
Independent variable of interest	*Outlier proportion*	*Max. Cook's distance*	*Corresponding figure*
age	*0*	*0.274*	*Figure 7B*

VDQ	0	0.164	Figure 7D
NVDQ	0	0.216	Figure 7F
Dependent variable: Laterality of Network 1 (“Language”), Dataset 1			
Independent variable of interest	Outlier proportion	Max. Cook’s distance	Corresponding figure
age	0	0.060	Supplementary Figure 25A
Language composite	0	0.057	Supplementary Figure 25C
Dependent variable: Laterality of Network 18 (“Default-Anterolateral”), Dataset 1			
Independent variable of interest	Outlier proportion	Max. Cook’s distance	Corresponding figure
age	0	0.037	Supplementary Figure 25B
Language composite	0	0.069	Supplementary Figure 25D

”

“Lateralization of the functional networks in Dataset 1
The left lateralization of networks were replicated in Dataset 1 (longitudinal data at 22-47 months), both the Language network (LI = 0.13 ± 0.16, one-sample t-test, $t(198) = 11.2807$, $p < 0.001$) and the Default Anterolateral network (LI = 0.44 ± 0.17, one-sample t-test, $t(198) = 37.0012$, $p < 0.001$) were left lateralized. Laterality of the Language network increased significantly with age ($\beta = 0.055$ per year, $SE = 0.016$, $t = 3.50$, $df = 91.9$, $p = 0.0007$, Supplementary Figure 25A), but the laterality of the Default-Anterolateral network did not ($\beta = 0.017$ per year, $SE = 0.020$, $t = 0.86$, $df = 140.6$, $p = 0.39$, Supplementary Figure 25B). Verbal ability was measured with Bayley Scales of Infant and Toddler Development age-normalized language composite scores, which was calculated from 49 items in the receptive and 48 items in the expressive domain(Balasundaram and Avulakunta, 2025). There is a weak positive association between laterality of the Language network ($\beta = 0.0016$ per composite score point, $SE = 0.0008$, $t = 0.86$, $df = 175.2$, $p = 0.052$, Supplementary Figure 25C) and the Bayley language composite scores that did not reach statistical significance. Similarly, the association between the Default-Anterolateral network laterality and the Bayley language composite scores did not reach significance ($\beta = 0.0012$ per composite score point, $SE = 0.0008$, $t = 0.86$, $df = 154.7$, $p = 0.144$, Supplementary Figure 25D). ”

6. Overall Recommendation

The manuscript would benefit from substantial reorganization, greater methodological transparency, and a more comprehensive review of relevant literature. The introduction and discussion sections should more clearly situate the study within the context of existing work, emphasizing its distinct contributions and methodological advances. A thorough rewrite focused on improving clarity, coherence, and scientific rigor is strongly recommended prior to resubmission.

Thank you for your constructive feedback. We have heavily edited our language and organization of the manuscript to improve clarity, coherence, and scientific rigor.

We appreciate the reviewers' time and effort to read and comment on our manuscript. Please see below for the response to specific points in blue, with the changes in text in *Italics*.

Reviewers' comments:

Reviewer #2 (Remarks to the Author):

The authors have addressed my comments appropriately in the revision.

Reviewer #4 (Remarks to the Author):

In "Commonality and Variability in Functional Networks In Children Under 5 Years Old", Tu and authors deploy and validate an individualized network approach in a cohort of young children. They then utilize this approach to specifically explore lateralization and behavioral associations of the language network. Overall, the paper demonstrates methodological novelty in using a precision functional network approach with such a young subject cohort, providing a useful resource for the field. Furthermore, I found the review and discussion of the existing literature to be very thorough. While I was not a reviewer for the original submission, I see that the authors have made extensive and meaningful changes to address the initial comments. However, I do share some of the previous reviewer's concerns regarding the clarity, organization, and claims within the manuscript – especially with the behavioral modeling. I have a couple of points that the authors could address to enhance the impact and rigor of the submission.

1) I find the overall organization and flow of the paper to be lacking in clear direction. The choice of modeling language specifically feels a bit arbitrary and unmotivated. Given the wide scope of the dataset generated, a more comprehensive evaluation of networks, metrics (such as lateralization), and behavioral associations is warranted to better motivate the focus on language specifically. Additionally, the emphasis on network definitions impedes the overall narrative and could be condensed. For instance, Table 1 could be moved to the supplemental document and the in-text discussion could be shortened.

We thank the reviewer for this constructive feedback. To better motivate the focus on language, we have edited the final paragraph of the introduction, which now reads:

"Here, we investigate functional network organization and its variability in children aged 1–5 years using two pediatric datasets with high-quality low-motion data. We first derive age-specific group-level network templates from extended scan sessions and then use these as priors to estimate vertex-level individual-specific networks from shorter acquisitions. This approach allows us to characterize fine-grained network organization that is not readily observable from group-average data, while enabling robust estimation in very young children.
Given the strong prior literature demonstrating early emergence and protracted development of hemispheric specialization—particularly for the language network—we focus our primary analyses on language-related systems as a well-characterized model of functional specialization during early development. Language networks provide a sensitive testbed for evaluating

whether individual-specific network organization captures meaningful neurodevelopmental variation, as they exhibit robust left-hemisphere lateralization in adults and show developmental strengthening across childhood. We therefore examine how language network lateralization emerges across early childhood and whether it relates to early behavioral language abilities."

To address concerns about narrative flow and the emphasis on network definitions, we have also moved the network correspondence table along with its associated text to the Supplementary Materials under the section "Finding the correspondence of networks to literature mentions."

2) The language behavior modeling analysis (figure 7) lacks depth and the proper motivation. Ex. Why was the default-anterolateral network chosen specifically to be the comparison? Furthermore, the effect sizes reported are very small with seemingly minimal differences between networks, the wording in the manuscript should better reflect this. A more comprehensive comparison across networks and behavioral measures would help. For instance, a statistically stringent identification/ranking of networks that associate with a given behavior. At the very least, a more detailed explanation for the analysis choices made and a more thorough behavioral comparison of the networks is warranted.

Thank you to the authors for their hard work and an interesting read!

We thank the reviewer for this comment and would like to clarify the motivation and design of this analysis. The selection of both networks was entirely hypothesis-driven, based on prior literature identifying both the language network and the default-anterolateral network as containing regions involved in language functions (Braga et al., 2020; Lipkin et al., 2022; Zhu et al., 2025), as detailed in Supplementary Table 1 and the relevant text in results section “We focused on two networks that contain regions commonly reported to be involved in language functions (Braga et al., 2020; Lipkin et al., 2022; Zhu et al., 2025): the language network (Network 1) and the default-anterolateral network (Network 18).” These two networks were not intended as a network-of-interest versus a comparison network — rather, both were selected as language-relevant networks to characterize developmental lateralization. The analysis was not designed as a contrast between networks, nor as a data-driven ranking across all networks, which would be exploratory in nature and introduce multiple comparison concerns beyond the scope of a hypothesis-driven study. We have revised the manuscript to make this framing clearer and to ensure the wording does not imply a competitive contrast between the two networks.

Specifically, we replaced this sentence:

"In contrast, laterality of the Default-Anterolateral network did not significantly associate with VDQ"

With:

"*The Default-Anterolateral network showed a similar trend, though this did not reach statistical significance ...*"

We also note that the raw β coefficients reported are in the units of the laterality index per behavioral score point and should not be interpreted as standardized effect sizes. The similar magnitude of associations across the two networks (β = 0.00122 vs. β = 0.00107 for VDQ) is in fact consistent with both being language-relevant networks, rather than reflecting a failure to detect differences. The difference in statistical significance between the two reflects differences in precision of estimation rather than a meaningful difference in effect magnitude. We have revised the manuscript wording to make this distinction clear.

Specifically, we added the standardized beta coefficients for each reported beta:
"Using linear mixed-effects models with a random intercept for subject, laterality of the Language network increased with age (β = 0.025 per year, SE = 0.011, t = 2.29, df = 299, p = 0.023, *standardized β = 0.131*, Figure 7A). Laterality of the Default-Anterolateral network also increased with age (β = 0.059 per year, SE = 0.015, t = 3.82, df = 299, p < 0.001, *standardized β = 0.216*, Figure 7B). These associations were robust to the addition of mean framewise displacement of the scan session.
Associations between network laterality and behavioral measures were assessed using linear mixed-effects models including age and behavioral scores as fixed effects and a random intercept for subject. Verbal and non-verbal abilities were quantified using the Mullen Verbal Developmental Quotient and Non-verbal Developmental Quotient (VDQ and NVDQ; age-normalized scores from the Mullen Scales of Early Learning (Mullen, 1995)), excluding sessions without available scores (21 without VDQ and 19 without NVDQ). Language network laterality was positively associated with verbal ability beyond the effect of age (β = 0.00122 per VDQ point, SE = 0.00044, t = 2.79, df = 271.6, p = 0.0057, *standardized β = 0.169*, Figure 7C). The Default-Anterolateral network showed a similar trend, though this did not reach statistical significance (β = 0.00107 per VDQ point, SE = 0.00062, t = 1.72, df = 269.1, p = 0.087, *standardized β = 0.102*, Figure 7D). To assess specificity to verbal ability, neither Language network nor Default-Anterolateral network laterality significantly associated with non-verbal ability (NVDQ). Language network laterality did not significantly associate with NVDQ (β = 0.00090 per NVDQ point, SE = 0.00049, t = 1.85, df = 254.0, p = 0.066, *standardized β = 0.115*, Figure 7E), nor did the Default-Anterolateral network (β = 0.00055 per NVDQ point, SE = 0.00069, t = 0.79, df = 247.2, p = 0.430, *standardized β = 0.0482,* Figure 7F). Associations in the same direction were observed in the longitudinal dataset (Dataset 1), but statistical significance was reduced potentially due to a smaller sample size, a more restricted age range centered around 2–3 years, and the use of the Bayley Scales of Infant and Toddler Development language composite as a proxy for verbal ability (Supplementary Materials, Supplementary Figure 26). "

And we softened the existing "control analysis" framing slightly, changing:

"As a control analysis, Language network laterality did not significantly associate with NVDQ..."

to:

"*To assess specificity to verbal ability, neither Language network nor Default-Anterolateral network laterality significantly associated with non-verbal ability (NVDQ)...*"
