# Supplementary material for: Commonality and variability in functional networks in children under 5 years old
Source: Commun Biol. 2026 Jul 7;9:1421. doi: 10.1038/s42003-026-10599-w (PMC13719990; doi:10.1038/s42003-026-10599-w)
Supplement: Supplementary file 2 [file 42003_2026_10599_MOESM2_ESM.pdf]

Supplementary Materials

Supplementary Note 1. Group-averaged FC may obscure finer individual-specific features present in individual participants' data

To illustrate this, consider each row of the parcel-by-parcel functional connectome as an FC profile—the connectivity pattern between one parcel and all others (**Supplementary Figure 1A**). These profiles can also be mapped onto a standard brain surface for spatial visualization (**Supplementary Figure 1A**). Averaging FC matrices from 81 template sessions (examples in **Supplementary Figure 1A & 1B**) yields a group-level FC (**Supplementary Figure 1C**). However, individual variability is evident in the distribution of pairwise correlations between FC profiles, such as those from the same lateral prefrontal cortex parcel across sessions (**Supplementary Figure 1D**). Some correlations are low, near or below zero (**Supplementary Figure 1D**, with an example pair in **Supplementary Figure 1E & 1F**), indicating weak consistency across individuals. Consequently, the group-averaged FC (**Supplementary Figure 1C & 1G**) may obscure finer individual-specific features in individual participants' data.

Supplementary Note 2. Comparison with alternative network templates from a generic group-average network atlas

An alternative to a data-driven clustered centroid on FC profiles of all individuals for the network templates (**Figure 1**) is to use the network assignments from group-average FC (Tu-19 networks[1], **Supplementary Figure 5**). For most networks that appear in both the data-driven and group-average priors, their spatial topographies were largely similar. However, the data-driven prior more finely differentiated certain regions (**Supplementary Figure 6**). For example, it separates the "Dorsal Somatomotor" found in the group-average prior into two distinct networks: a refined "Dorsal Somatomotor" network, encompassing hand and foot effector regions, and a "Somatic-Cognitive Action" network for integrating goals, physiology and body movement[2]. Additionally, when using the group-average prior, we observed broader regions with low correlation to templates around network boundaries (**Supplementary Figure 7**). This likely reflects the fact that the group-average templates are spatially blurred and may contain mixed signals from adjacent networks, leading to low-confidence assignments at the edges under the winner-takes-all matching approach.

Supplementary Note 3. Finding the correspondence of networks to literature mentions

We used two major toolboxes to put our networks in context with prior literature mentions. Specifically, we assessed the spatial correspondence between our 23 functional networks and canonical networks from prior literature using the "Network Correspondence Toolbox" version 0.2.0[3], and we reported the most frequently reported labels to our network topography using the "Neurosynth discrete decoding" in "NiMARE: Neuroimaging Meta-Analysis Research Environment" version 0.5.0[4]. Both are open-source Python-based software for neuroimaging meta-analyses.

For the analysis with the Network Correspondence Toolbox, nine representative atlases from different research groups were selected as references: "Du2024-15"[5], "Woodward2024-Task-12"[6], "Yan2023-400+Kong2019-17"[7,8], "Gordon2024-5"[2], "Gordon2017-17"[9], "Shen2013-268-8"[10] and "Glasser2016-360+Ji2019-12"[11,12], "Tu2025-19"[1], "Kardan2022-10"[13]. All reference atlases were projected to the fs_LR-32k surface space to

match the input data using the toolbox. A binarized map with network locations that are present with probability greater than 0.35 in the 81 template sessions was compared with the references using a Dice similarity coefficient. To determine statistical significance, we applied a spin test with 1000 surface-based rotations of each input map, generating a null distribution of Dice coefficients. The empirical p-value was defined as the proportion of rotated Dice values exceeding the observed Dice coefficient. Reference networks with $p < 0.05$ were considered to exhibit significant spatial similarity. The significantly overlapping networks in each atlas and the corresponding dice coefficient are visualized in circular bar chart plots (**Supplementary Figures 8-19**), and the raw dice coefficients and p-value statistics of all networks in all atlases are available in csv files at Supplementary Data. For all but one atlas (a.k.a. "Woodward2024-Task-12"), the network acronyms are intuitive and have been used widely in the systems neuroscience literature. However, the task-based network acronyms are less commonly encountered, so we provide a key here for reference:

1RESP – Response One-Handed
2RESP – Response Two-Handed
AAR – Auditory Attention Response
AUD – Auditory Primary Sensory
DMNA – DMN Novel
DMNB – DMN Traditional
FoVF – Focus on Visual Features
INIT – Initiation
LN – Language
MAIN – Maintaining
MDN – Multiple Demand
RE-EV – Re-evaluation

For the analysis with the "NiMARE toolbox, we first created a binarized map with network locations that are present with probability > 0.35 and transformed it to the MNI space using the Connectome Workbench command "metric-to-volume-mapping" with the nearest vertex interpolation method and filled in the holes with "-volume-fill-holes". Subsequently, we employed the "ROIAssociationDecoder" tool to perform a region-based discrete decoding by correlating each binarized map with term-specific meta-analytic maps. These maps were derived from Neurosynth v7, a large-scale database of over 14,000 fMRI studies[14]. Following standard Neurosynth procedures, we used annotations based on a vocabulary of 3,229 curated cognitive and functional terms. These terms were selected from study abstracts using a Term Frequency–Inverse Document Frequency (TF-IDF) approach, which prioritizes words that are frequent in specific documents but uncommon across the full corpus. This selection process ensures that the vocabulary contains meaning and discriminative concepts in neuroimaging literature. For each of the 23 binarized maps, NiMARE computed the Pearson correlation coefficient with every term's meta-analytic activation map, which quantifies their spatial alignment. From the resulting associations, the 15 top-ranked terms per map were selected to represent the dominant cognitive functions associated with the network. To facilitate interpretation, we excluded general terms such as "network", "cortex", "area", "lobe", "region",

"system", "activation", "brain", "functional", "related", "associated", "healthy", "task", "mode" and "connectivity". Morphological variants of the same word, such as past-tense verbs and plural nouns, were removed through lemmatization using the Natural Language Toolkit Python package[15] version 3.9.1. These selected terms were visualized as word clouds, where font size was scaled by the strength of the correlation to the meta-analytic activation maps (N.B. same scales were used for all networks), to highlight the most relevant labels for each network.

To aid interpretation of the function of these networks and put our networks in the context of existing findings, we made a table summary of previous nomenclature applied to similar network topography in the literature (**Supplementary Table 1**), with the caveat that this is only a guideline and some networks may not perfectly overlap with what we described here. This table was made both with the quantitative spatial and connectivity correspondence measures mentioned above, and with expert visual assessment of spatial correspondence, used to complement the quantitative measures.

Supplementary Table 1. Network names and prior mentions

**Index**	**Network name**	**Prior network definitions in literature that overlap with this network**
1	Language	Language/auditory[5,12,16,17], language[18], auditory language[19], auditory[7], ventral attention[20,21]
2	Frontoparietal	Frontoparietal[9,17], anterior frontoparietal[22,23], lateral prefrontal[1]
3	Dorsal Attention	Dorsal attention[17], Salience[16], CO1/task-control cingulo-opercular subsystem[24], premotor-posterior parietal rostral[5]
4	Visual Central	Visual central[5,25]
5	Parietal Memory	Parietal memory[9,17], Medial parietal[26]
6	Ventral Insula	Ventral insula[27], orbito-affective[12], limbic[25,28], ventral anterior insula[29]
7	Premotor/Dorsal Attention II	Premotor/Dorsal attention II[17]
8	Default-Dorsolateral	Default-dorsolateral[17,30],
9	Ventral Somatomotor	Ventral somatomotor[26],Somatomotor face[9,17], Lateral somatomotor[31]
10	Default - Dorsal prefrontal	Dorsal prefrontal[32], prefrontal cortex[33]
11	Context association	Context association[9], parietal-occipital[26,31], default-retrosplenial[17]

12	Somato-cognitive-action	Somato-cognitive-action[2,17]
13	Default-Parietal	Default-parietal[17,30], posterior parietal[32]
14	Visual Peripheral	Visual peripheral[5,25]
15	Action Mode	Action mode[34,35], cingulo-opercular[9,24,26,31]
16	Posterior fronto-parietal	Posterior frontoparietal[1,22,23]
17	Visual-Streams	Visual-streams[17], visual association[10], dorsal attention[5]
18	Default-Anterolateral	Default-anterolateral[17,30], language[16], default semantic [19]
19	Default-Pregenual	Default-pregenual[30], pregenual medial prefrontal cortex (mPFC)[36], pregenual anterior cingulate (pACC)[37]
20	Default-Temporal	Temporal[23]
21	Salience	Salience[9,17,26,31]
22	Anterior Prefrontal	Anterior prefrontal[1]
23	Dorsal Somatomotor	Dorsal somatomotor[26], Somatomotor[31], Somatomotor-hand[9,17]

Supplementary Note 4. Replicating the split-half reliability analysis of individual-specific functional networks with no overlapping runs across split halves

For the split-half reliability analysis of individual-specific functional networks, we evenly divided the low-motion frames across all fMRI runs in a single scan session to maximize the amount of data per split half. However, due to temporal autocorrelations and shared preprocessing, having neighboring frames within a run in the two split halves could artificially inflate the NMI. Therefore, we repeated the same analysis but calculated the split-halves, ensuring no overlapping runs in the two split halves (e.g., the middle of 5 runs is dropped). The overall NMI is lower, potentially due to both less data per split half and reduced temporal dependence between split halves, but the conclusions remain the same (**Supplementary Figure 23**). The consistency of network assignment across the split halves (measured with normalized mutual information, NMI) was still significantly higher (two-sample t-test $p<0.001$) within individual (Y2: NMI = 0.4416 ± 0.0934, Y3: NMI = 0.4710 ± 0.0882) than between individuals (Y2: NMI = 0.3037 ± 0.0517, Y3: NMI = 0.3123 ± 0.0409).

Supplementary Note 5. Effect of data amount on longitudinal network stability

Because within-subject NMI is higher with more data, we conducted additional analysis to assess the effect of the data amount on longitudinal network stability in the 49 subjects with longitudinal sessions by sampling parts of the data from the whole session. Notably, due to the

temporal autocorrelation in BOLD data, random sampling of the same number of fMRI volumes (TRs) within a long window tends to increase data reliability than contiguous data sampling[31,38]. Therefore, the use of the term “minutes” to refer to random sampling can be misleading and not really informative for future data collection. We sampled contiguous data for the number of frames that approximately match the amount of target data length based on a TR of 0.8 seconds in the fMRI data to better simulate actual data collection. For example, we sampled the first 375 frames of low-motion data to obtain a target length of 5 minutes. Because of the frame censoring procedure, the acquired data length is often slightly greater than 5 minutes and may be variable across subjects, but we omit that detail for simplicity. Since there is a sharp drop-off in available subjects at a threshold of 20 minutes’ low-motion data (**Supplementary Figure 26A**), we sampled data segments with lengths of 2.5 to 20 minutes at a step of 2.5 minutes for both Y2 and Y3 data. The number of available subjects went down with longer data length, with only 11 subjects in the most sparse bin (20 minutes in Y2 and 20 minutes in Y3). When comparing the networks obtained from an increasing amount of data from 5 minutes to 20 minutes in Y3 to networks obtained from a fixed 20 minutes of data in Y2, both within and between network NMI increases (**Supplementary Figure 26B**), but within-subject NMI is always higher than between-subject NMI. We also calculated the mean within-subject (**Supplementary Figure 26C**) and between-subject (**Supplementary Figure 26D**) NMI for each data-length pair, and found that increasing the data amount in both Y2 and Y3 increased the within-subject and between-subject NMI, but the within-subject NMI is always higher than the respective between-subject NMI. The difference between within- and between-subject NMI is significant even when both Y2 and Y3 have 2.5 minutes of low-motion data $t(2399) = 14.30$, $p < 0.0001$ (**Supplementary Table 2**), and the 83.4% confidence interval started to have no overlap before 7.5 minutes. An 83.4% confidence interval was deemed more suitable for evaluating the difference between two means rather than comparing a mean to a fixed value, as this approach maintains a Type I error rate close to $\alpha = 0.05$ when the standard errors of the samples are comparable[39,40].

Supplementary Table 2. NMI between longitudinal sessions with different amounts of low-motion minutes.

Minutes	Within	Between	Degrees of freedom	T-stat	P
2.5	0. 2415 ± 0.0475	0. 1790 ± 0.0208	2399	14.30	<0.0001
5	0. 3511 ± 0.0467	0. 2604 ± 0.0308	2399	20.17	<0.0001
7.5	0. 4145 ± 0.0425	0. 3054 ± 0.0297	2399	25.19	<0.0001
10	0. 4564 ± 0.0392	0. 3339 ± 0.0299	2350	27.91	<0.0001
12.5	0. 4839 ± 0.0369	0. 3519 ± 0.0306	2062	27.54	<0.0001
15	0. 5038 ± 0.0376	0. 3643 ± 0.0316	1888	27.13	<0.0001
17.5	0. 5250 ± 0.0379	0. 3744 ± 0.0333	804	18.94	<0.0001

20	0. 5371 ± 0.0460	0. 3848 ± 0.0343	570	14.47	<0.0001
*Statistics from a two-sample t-test (two-tailed).					

Supplementary Note 6. Lateralization of the functional networks in Dataset 1

The left lateralization of networks were replicated in Dataset 1 (longitudinal data at 22-47 months), both Network 1 (LI = 0.13 ± 0.16, one-sample t-test, $t(198) = 11.2807$, $p < 0.001$) and Network 18 (LI = 0.44 ± 0.17, one-sample t-test, $t(198) = 37.0012$, $p < 0.001$) were left lateralized. Laterality of Network 1 increased significantly with age ($\beta = 0.055$ per year, $SE = 0.016$, $t = 3.50$, $df = 91.9$, $p = 0.0007$, **Supplementary Figure 28A**, *standardized* $\beta = 0.202$), but the laterality of Network 18 did not ($\beta = 0.017$ per year, $SE = 0.020$, $t = 0.86$, $df = 140.6$, $p = 0.39$, *standardized* $\beta = 0.059$, **Supplementary Figure 28B**). Verbal ability was measured with Bayley Scales of Infant and Toddler Development age-normalized language composite scores, which was calculated from 49 items in the receptive and 48 items in the expressive domain[41]. There is a weak positive association between laterality of the Language network ($\beta = 0.0016$ per composite score point, $SE = 0.0008$, $t = 0.86$, $df = 175.2$, $p = 0.052$, *standardized* $\beta = 0.147$, **Supplementary Figure 28C**) and the Bayley language composite scores that did not reach statistical significance. Similarly, the association between the Default-Anterolateral network laterality and the Bayley language composite scores did not reach significance ($\beta = 0.0012$ per composite score point, $SE = 0.0008$, $t = 0.86$, $df = 154.7$, $p = 0.144$, , *standardized* $\beta = 0.109$, **Supplementary Figure 28D**).

Supplementary Note 7. Outlier analysis

To assess whether associations between network laterality and age or behavioral measures were driven by extreme observations or influential participants, we conducted residual and influence diagnostics for all linear mixed-effects models. Specifically, we computed the proportion of observations exceeding a magnitude of 3 standardized residuals and calculated subject-level Cook's distance, which quantifies the influence of each participant on fixed-effect estimates. For each association, the proportion of extreme residuals and the maximum Cook's distance are reported in **Supplementary Table 3**. Across all analyses, the proportion of observations exceeding a magnitude of 3 standardized residuals was small (≤0.3%), and Cook's distance values were modest, with no participant exerting disproportionate influence on model estimates. Accordingly, no outcome-based exclusions were applied.

Supplementary Table 3. Outlier analysis

Dependent variable: Laterality of Network 1 ("Language"), Dataset 2			
Independent variable of interest	Outlier proportion	Max. Cook's distance	Corresponding figure
age	0.003	0.064	Figure 7A
VDQ	0	0.102	Figure 7C
NVDQ	0	0.200	Figure 7E

Dependent variable: Laterality of Network 18 (“Default-Anterolateral”), Dataset 2			
Independent variable of interest	Outlier proportion	Max. Cook’s distance	Corresponding figure
age	0	0.274	Figure 7B
VDQ	0	0.164	Figure 7D
NVDQ	0	0.216	Figure 7F
Dependent variable: Laterality of Network 1 (“Language”), Dataset 1			
Independent variable of interest	Outlier proportion	Max. Cook’s distance	Corresponding figure
age	0	0.060	Supplementary Figure 28A
Language composite	0	0.057	Supplementary Figure 28C
Dependent variable: Laterality of Network 18 (“Default-Anterolateral”), Dataset 1			
Independent variable of interest	Outlier proportion	Max. Cook’s distance	Corresponding figure
age	0	0.037	Supplementary Figure 28B
Language composite	0	0.069	Supplementary Figure 28D

Supplementary Note 8. Non-parametric permutation test for brain-behavioral relationship

To validate the statistical significance of associations between functional network laterality and behavioral measures, we performed nonparametric permutation testing in addition to the parametric linear mixed-effects analyses reported in the main text. Because behavioral measures varied across scan sessions and some children contributed multiple scans, permutation testing was implemented using a subject-level residual sign-flipping procedure derived from a reduced mixed-effects model[42]. Specifically, we first fit a reduced model including age as a fixed effect and a random intercept for subject (LI ~ age + (1 | subject)) to obtain fitted values and residuals under the null hypothesis of no behavioral effect. Residuals were then randomly sign-flipped at the subject level, preserving the within-subject dependence structure and the effects of age. For each permutation, a permuted outcome was reconstructed by adding the sign-flipped residuals to the fitted values, and the full mixed-effects model including the behavioral predictor was refit. Repeating this procedure across 1000 permutations yielded a null distribution of the behavioral regression coefficient, against which the observed coefficient was compared to obtain an empirical two-sided permutation p-value (minimum 0.001).

Permutation testing confirmed that the observed association between Language network laterality and VDQ remained statistically significant (p_perm = 0.012), consistent with the parametric mixed-effects result reported in the main text. In contrast, associations involving

NVDQ did not reach significance under permutation testing (p_perm = 0.150), in line with parametric estimates. Default Anterolateral network and VDQ (p_perm = 0.116) or NVDQ (p_perm = 0.533) associations were also not significant. Together, these results indicate that the reported language-specific laterality effects are robust to distributional assumptions and are not driven by violations of model assumptions or repeated-measures structure.

Supplementary Figures

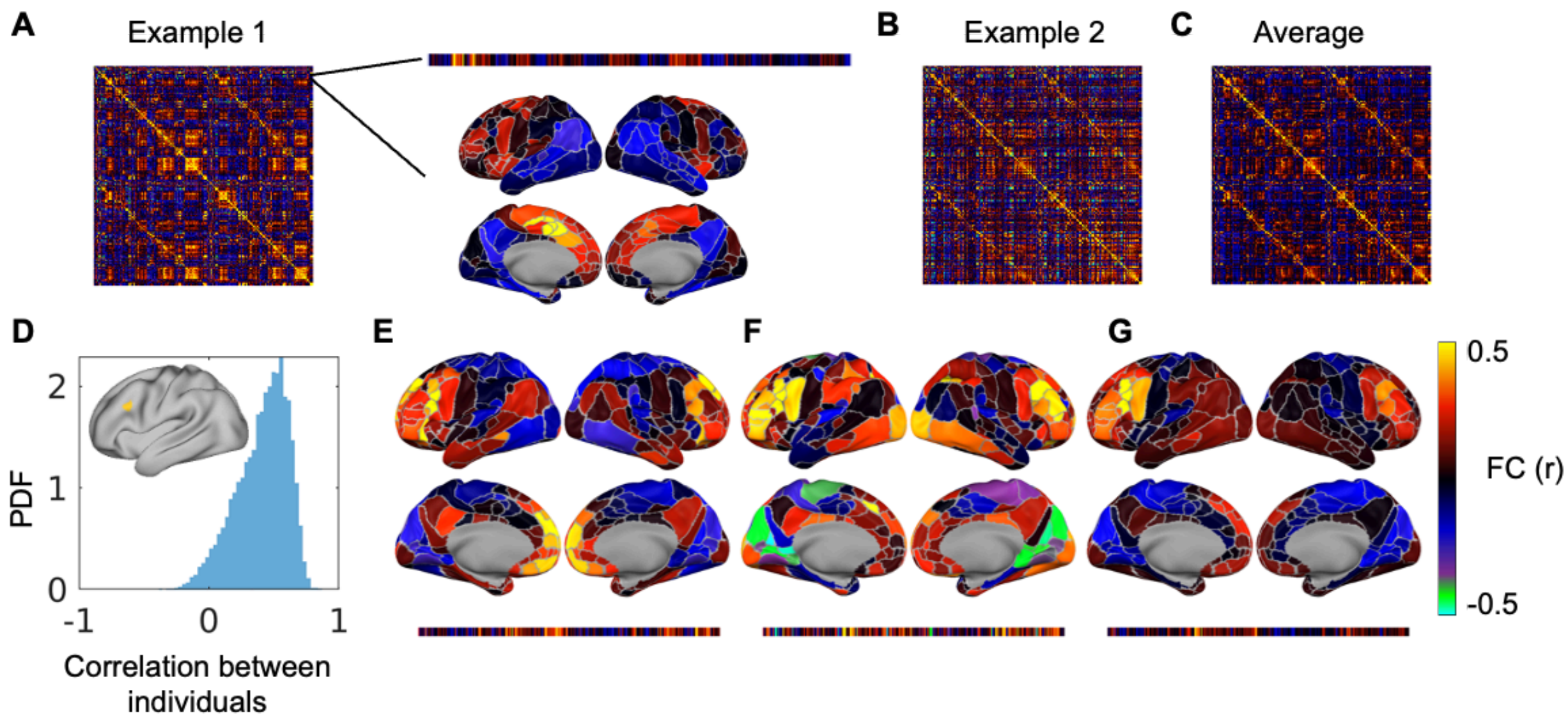

Supplementary Figure 1. *Functional connectivity (FC) variability across individuals.* A) Parcel-to-parcel FC matrix from one example session. The individual rows can be seen as the FC from one example parcel to all cortical parcels, which can also be visualized on a standard brain surface. B) FC from another session. C) Average FC across 81 fMRI sessions with > 20 minutes of low-motion data from Dataset 1. D) The correlation between pairs of FC profiles (out of 81 sessions) from one example parcel at the lateral prefrontal cortex. E-F) Example pairs of FC profiles of the example parcel. G) Average FC from the example parcel across 81 template sessions.

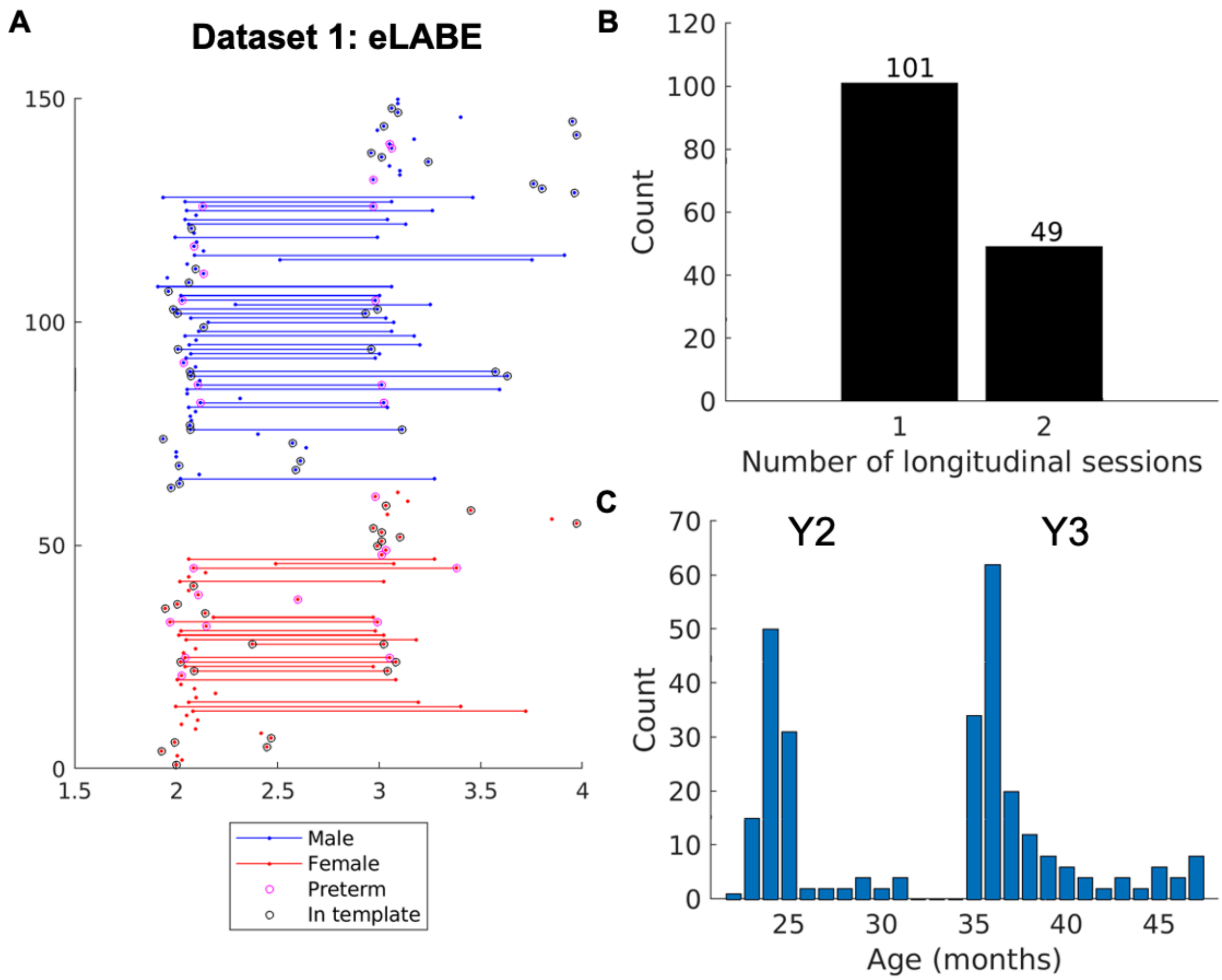

Supplementary Figure 2. *Dataset demographics (eLABE).* A) The age at fMRI session for each participant, with participants sorted by Male (blue) and Female (red), with preterm participants marked with magenta circles and sessions used in creating the template marked with black circles. B) Number of participants with 1-6 longitudinal sessions. C) Number of sessions each month.

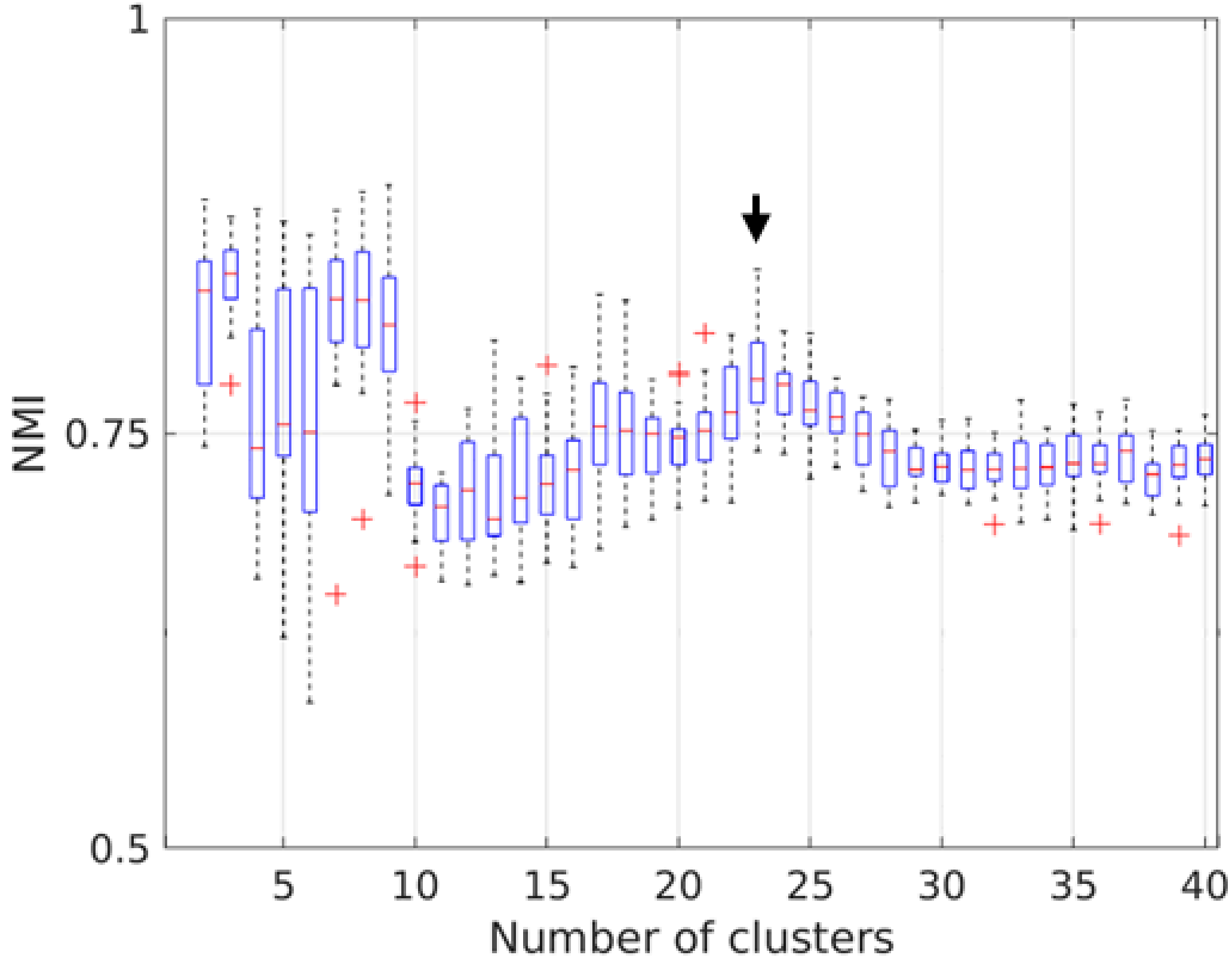

Supplementary Figure 3. *K-means clustering stability across 20 split-half samples of sessions.*

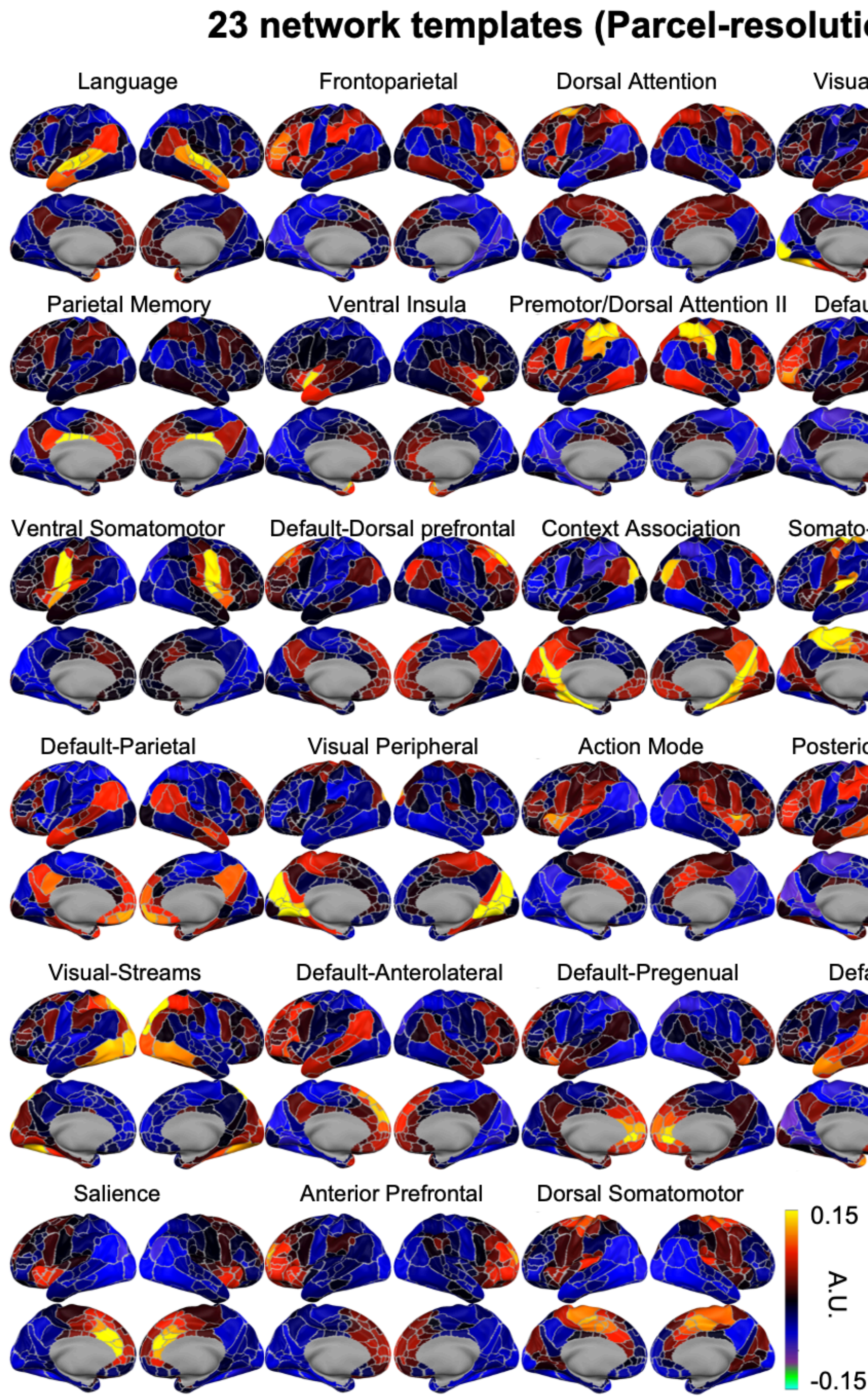

Supplementary Figure 4. *23 network templates at the resolution of parcels.* The values represent the mean of normalized vectors (a.k.a. cluster centroids using correlational distance) in arbitrary units (A.U.).

Tu-19 network templates (parcel-resolution)

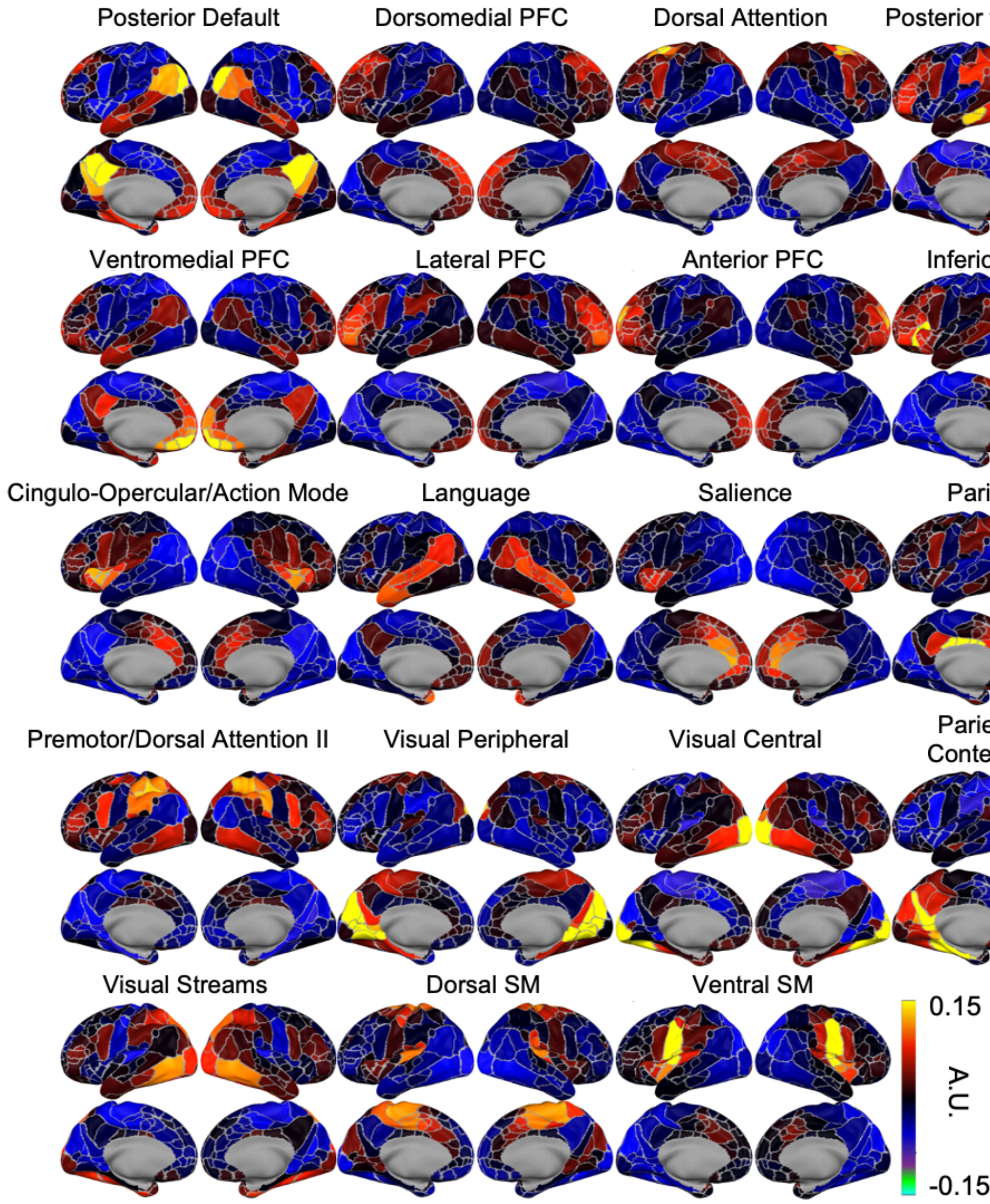

Supplementary Figure 5*. Tu-19 network templates at the resolution of parcels. PFC, prefrontal cortex. SM, Somatomotor.* The values represent the mean of normalized vectors (a.k.a. cluster centroids using correlational distance) in arbitrary units (A.U.).

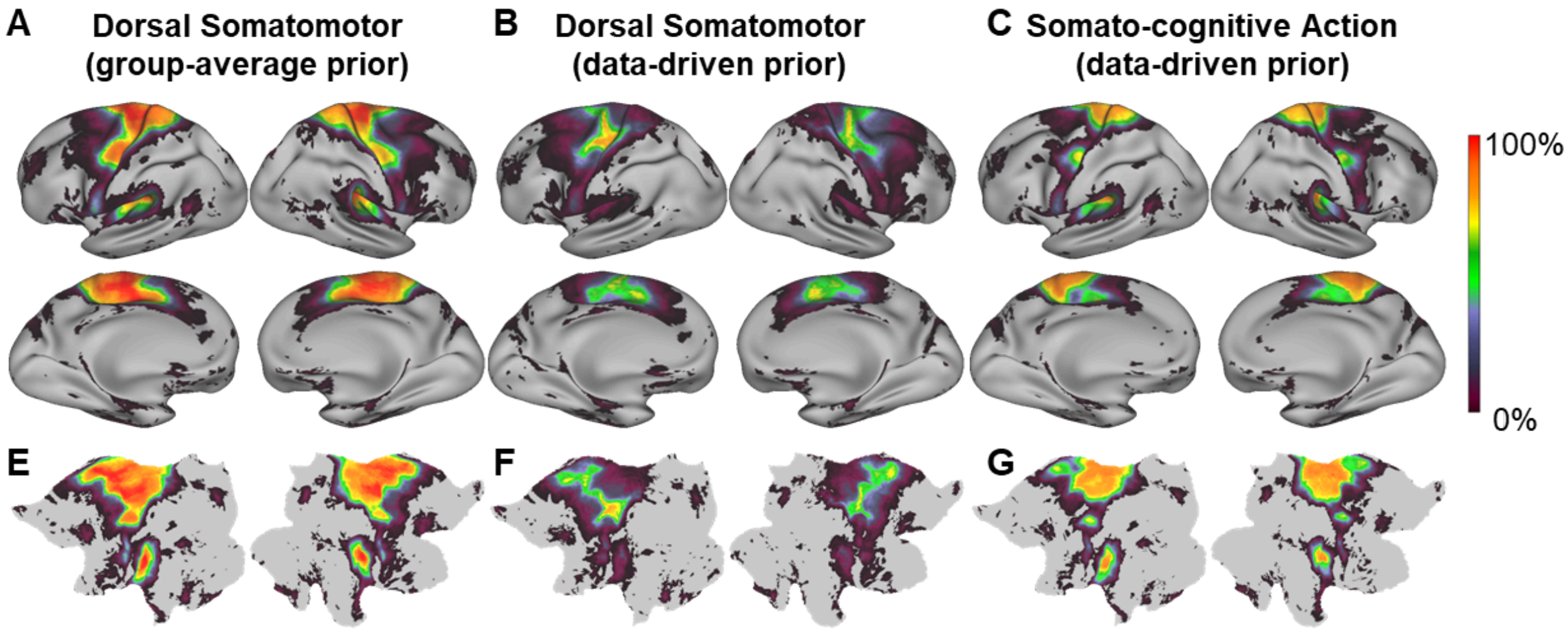

Supplementary Figure 6. *Network spatial topography of similar networks using group-average prior versus data-driven prior.* A) Dorsal Somatomotor (group-average prior). B) Dorsal Somatomotor (data-driven prior). C) Somato-cognitive Action (data-driven prior). D-F) Same as A-C but viewed in flat maps.

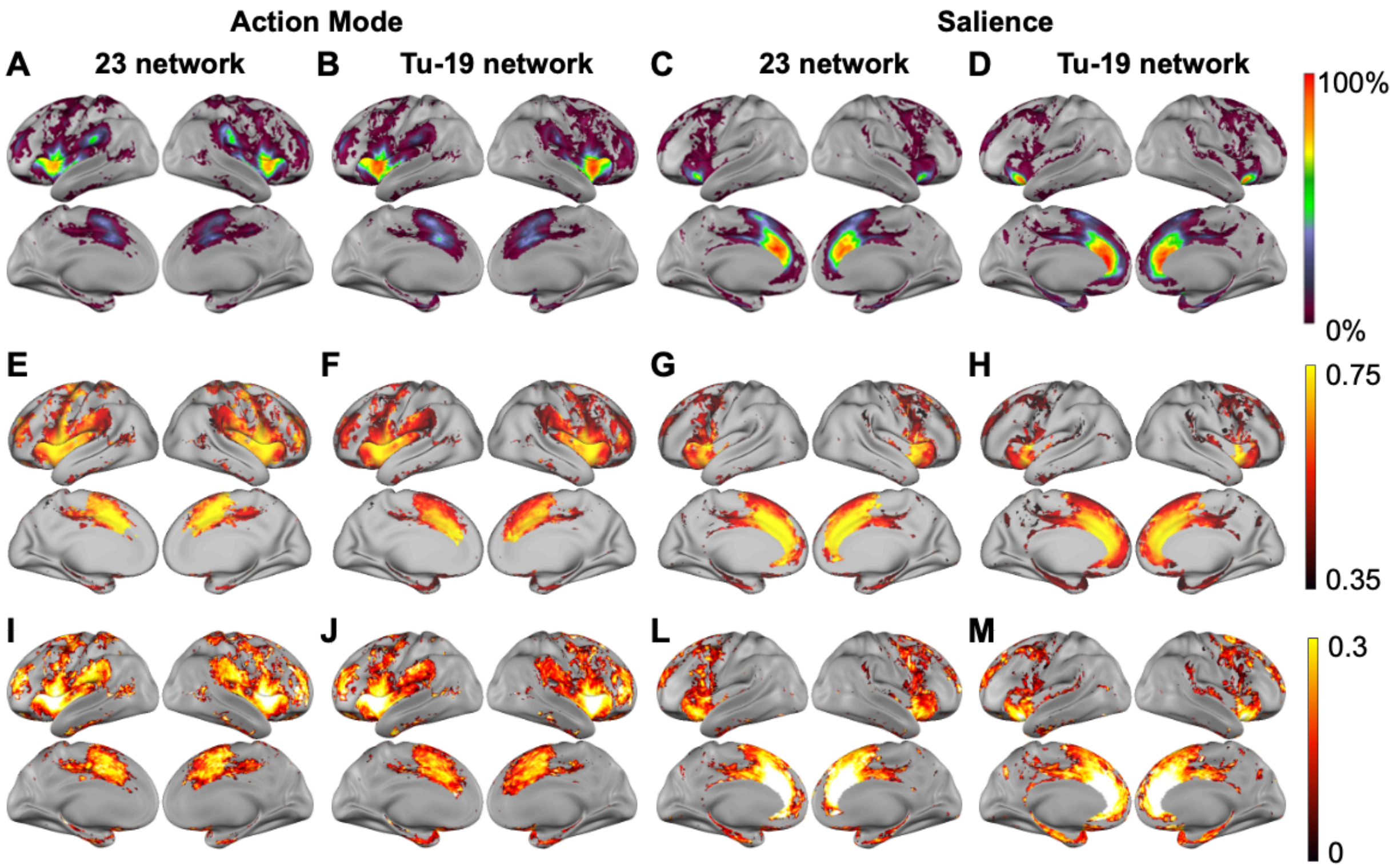
Action Mode
Salience
A
23 network
B
Tu-19 network
C
23 network
D
Tu-19 network
100%
0%
E
F
G
H
0.75
0.35
I
J
L
M
0.3
0

Supplementary Figure 7. *Comparing the network topography of the same network using different network templates*. A-B) Spatial probability E-H) Mean Pearson's correlation to the network template. I-M) Silhouette Index (Confidence) with correlational distance.

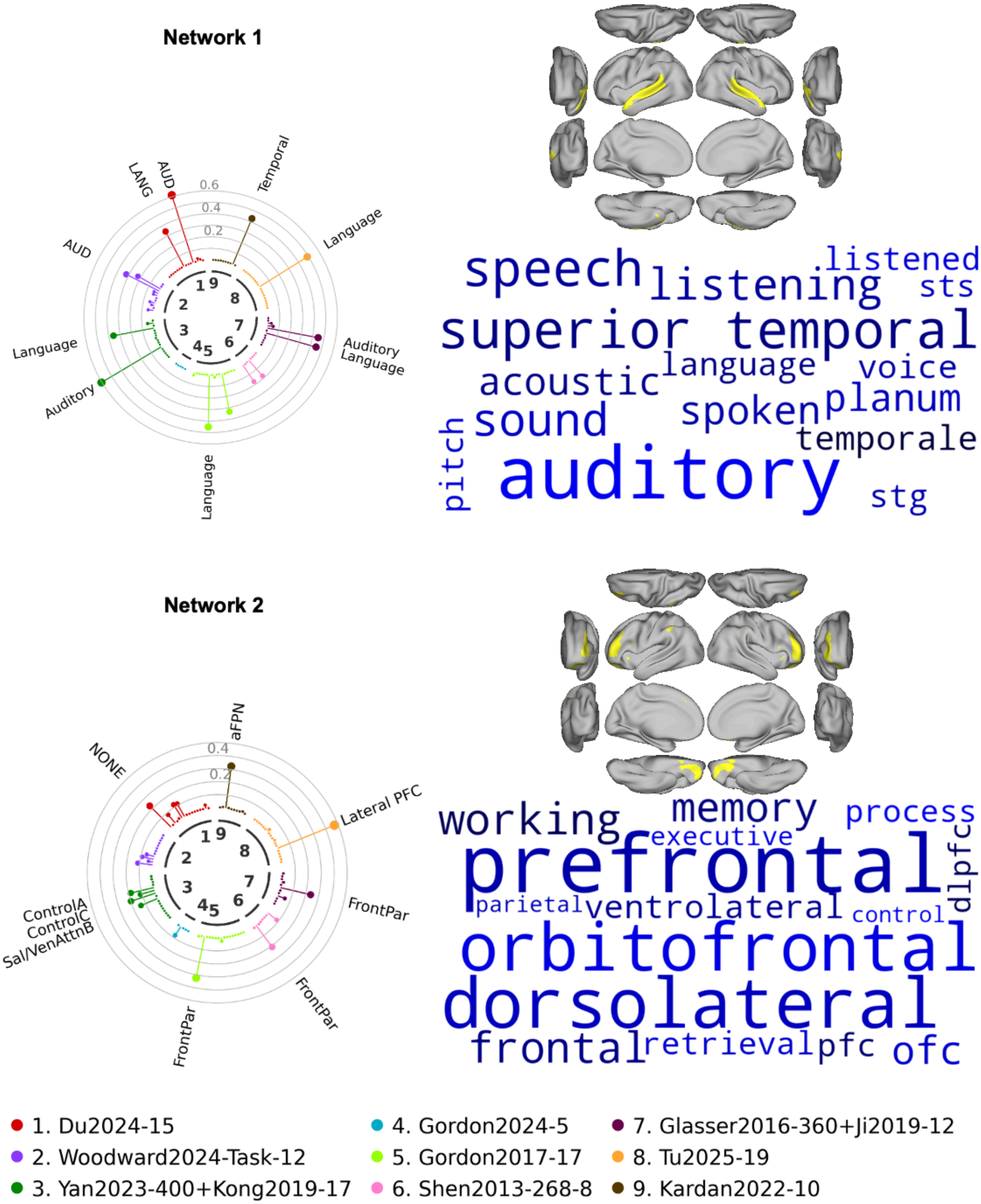

Supplementary Figure 8. *Correspondence between the 23 networks and previously reported functional networks/decoded terms (Networks 1-2)*. Circular bar chart plot from the network correspondence toolbox for spatial correspondence with the network locations with >0.35 probability and 7 atlases from the literature. Circles illustrate the magnitude of the dice

coefficient in intervals of 0.1. Only significant networks from the spatial permutation test ($p<0.05$) were named. Top right: the network locations with >0.35 probability visualized on a template brain surface. Top bottom: terms with highly correlated meta-analytic activation maps to the network topography.

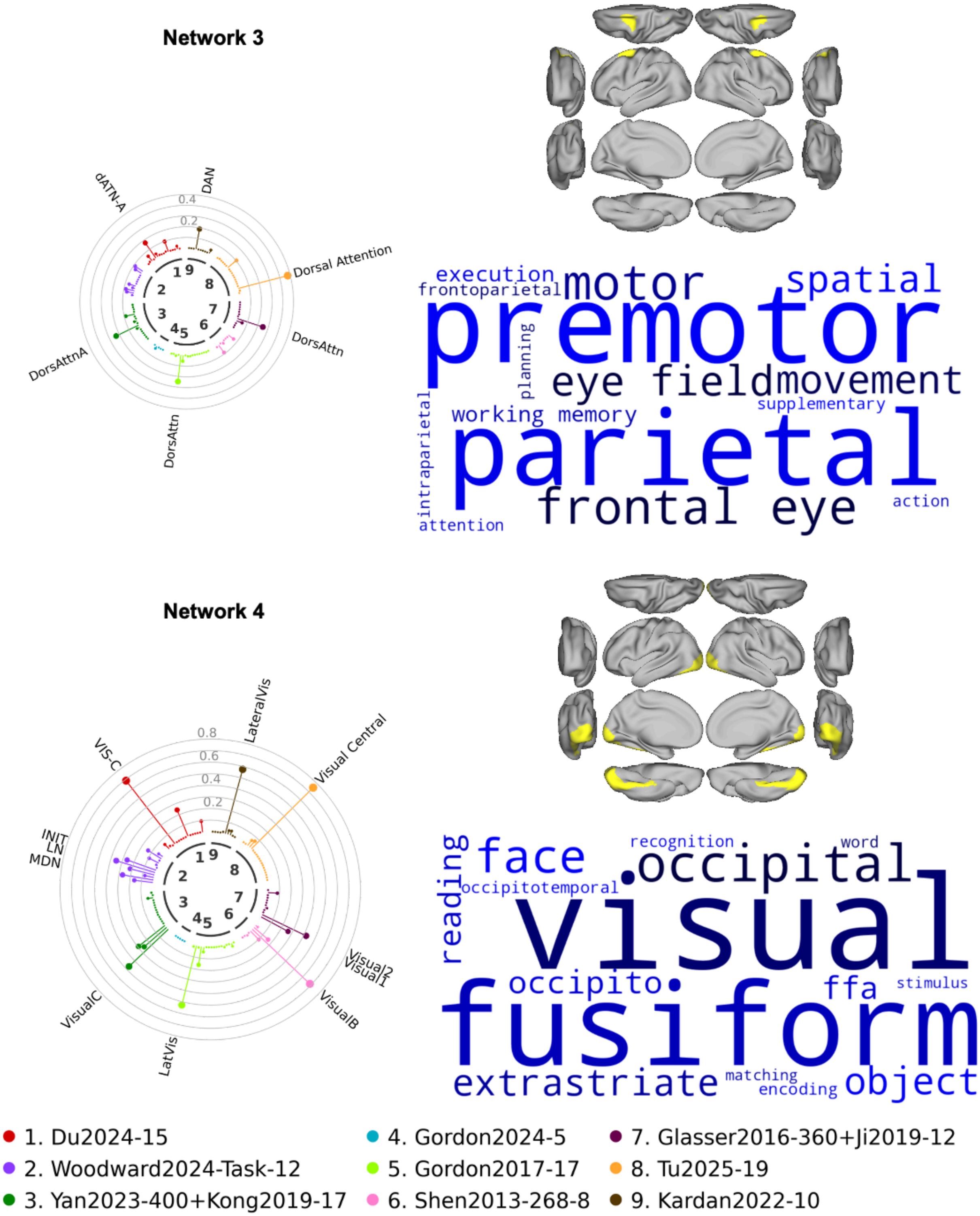

Supplementary Figure 9. *Correspondence between the 23 networks and previously reported functional networks/decoded terms (Networks 3-4).*Left: Circular bar chart plot from the network correspondence toolbox for spatial correspondence with the network locations with >0.35 probability and 7 atlases from the literature. Circles illustrate the magnitude of the dice

coefficient in intervals of 0.1. Only significant networks from the spatial permutation test ($p<0.05$) were named. Top right: the network locations with >0.35 probability visualized on a template brain surface. Top bottom: terms with highly correlated meta-analytic activation maps to the network topography.

Network 5

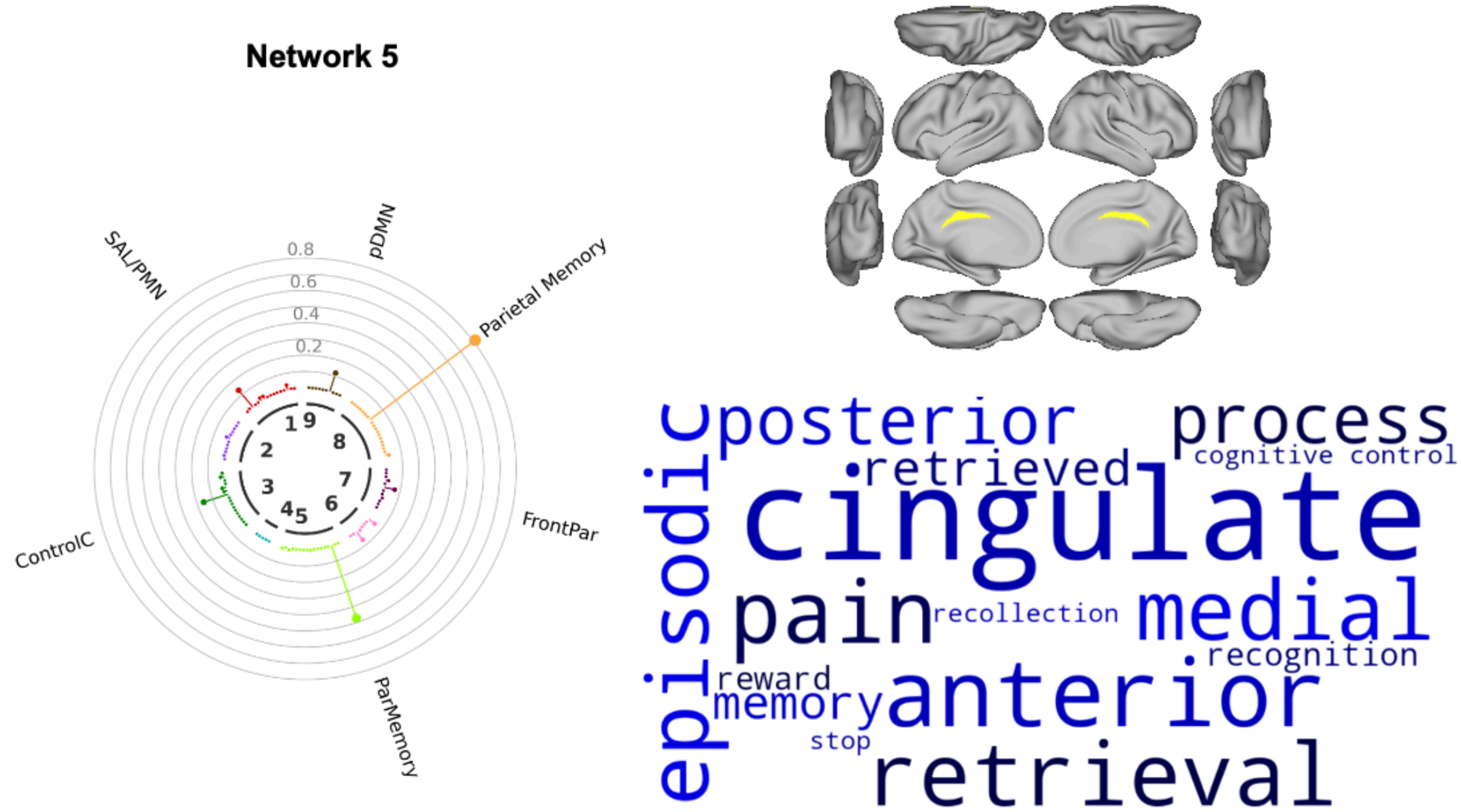

Network 6

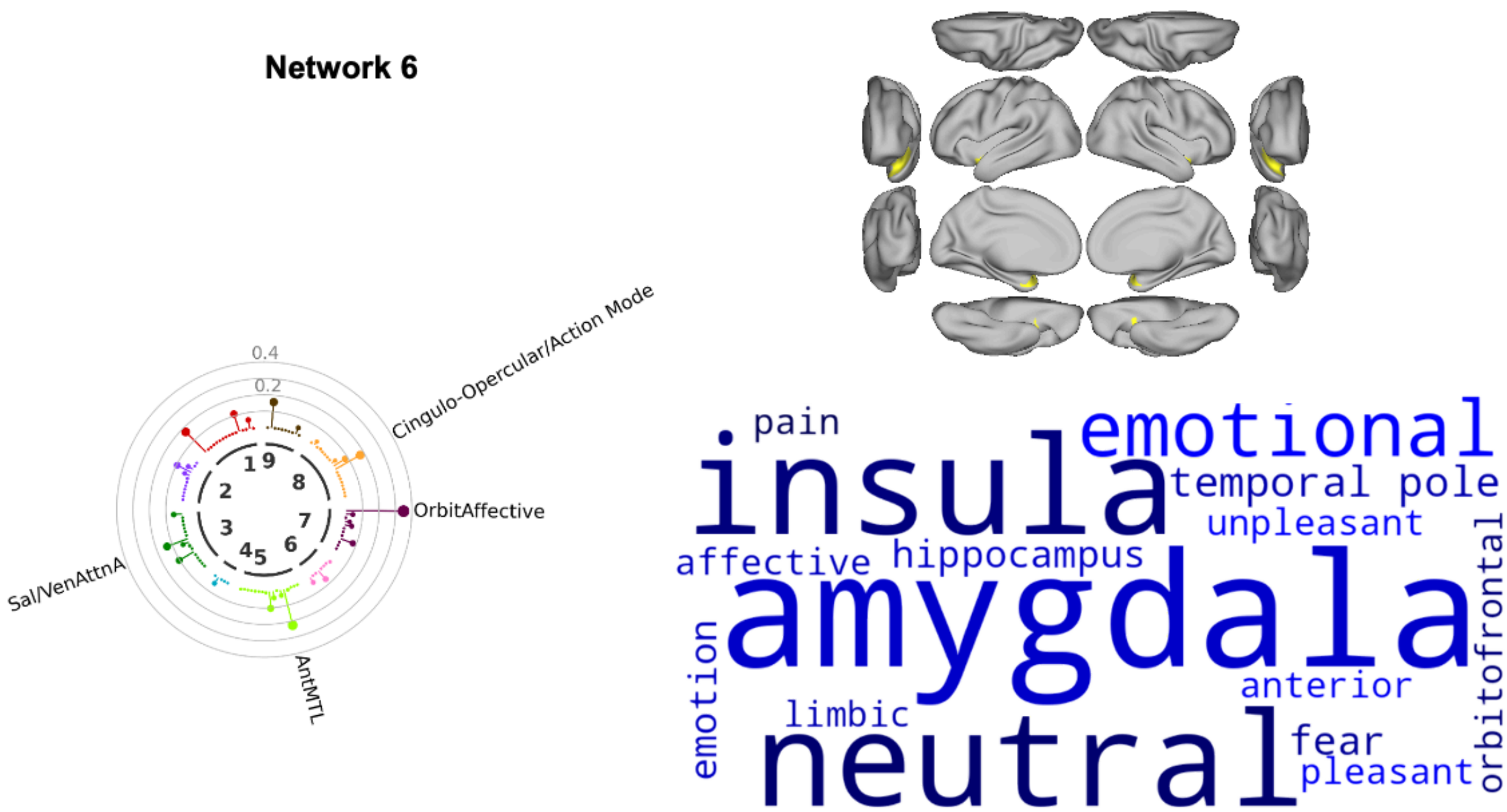

- 1. Du2024-15
- 2. Woodward2024-Task-12
- 3. Yan2023-400+Kong2019-17
- 4. Gordon2024-5
- 5. Gordon2017-17
- 6. Shen2013-268-8
- 7. Glasser2016-360+Ji2019-12
- 8. Tu2025-19
- 9. Kardan2022-10

Supplementary Figure 10. *Correspondence between the 23 networks and previously reported functional networks/decoded terms (Networks 5-6)*. Left: Circular bar chart plot from the network correspondence toolbox for spatial correspondence with the network locations with >0.35 probability and 7 atlases from the literature. Circles illustrate the magnitude of the dice coefficient in intervals of 0.1. Only significant networks from the spatial permutation test ($p<0.05$) were named. Top right: the network locations with >0.35 probability visualized on a template brain surface. Top bottom: terms with highly correlated meta-analytic activation maps to the network topography.

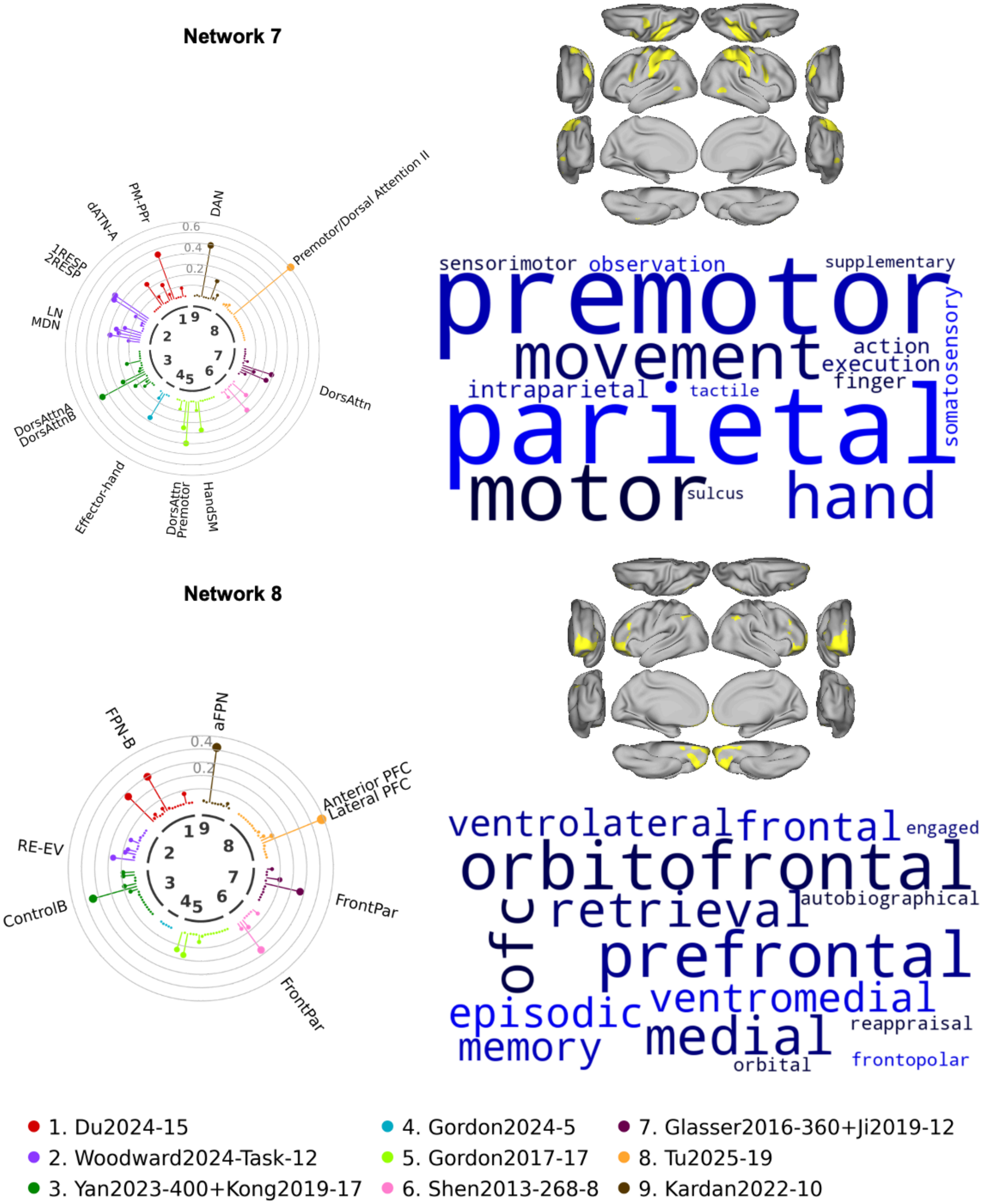

Network 7
DAN
PM-PPr
dATN-A
1RESP
2RESP
LN
MDN
Premotor/Dorsal Attention II
DorsAttn
DorsAttnA
DorsAttnB
Effector-hand
DorsAttn
Premotor
HandSM
0.6
0.4
0.2
sensorimotor
observation
supplementary
premotor
movement
action
execution
finger
somatosensory
intraparietal
tactile
parietal
motor
sulcus
hand
Network 8
FPN-B
aFPN
Anterior PFC
Lateral PFC
RE-EV
ControlB
FrontPar
FrontPar
0.4
0.2
ventrolateral
frontal
engaged
orbitofrontal
ofc
retrieval
autobiographical
prefrontal
episodic
ventromedial
memory
medial
reappraisal
orbital
frontopolar
1. Du2024-15
2. Woodward2024-Task-12
3. Yan2023-400+Kong2019-17
4. Gordon2024-5
5. Gordon2017-17
6. Shen2013-268-8
7. Glasser2016-360+Ji2019-12
8. Tu2025-19
9. Kardan2022-10

Supplementary Figure 11. *Correspondence between the 23 networks and previously reported functional networks/decoded terms (Networks 7-8)*. Left: Circular bar chart plot from the network correspondence toolbox for spatial correspondence with the network locations with >0.35 probability and 7 atlases from the literature. Circles illustrate the magnitude of the dice coefficient in intervals of 0.1. Only significant networks from the spatial permutation test ($p<0.05$) were named. Top right: the network locations with >0.35 probability visualized on a template brain surface. Top bottom: terms with highly correlated meta-analytic activation maps to the network topography.

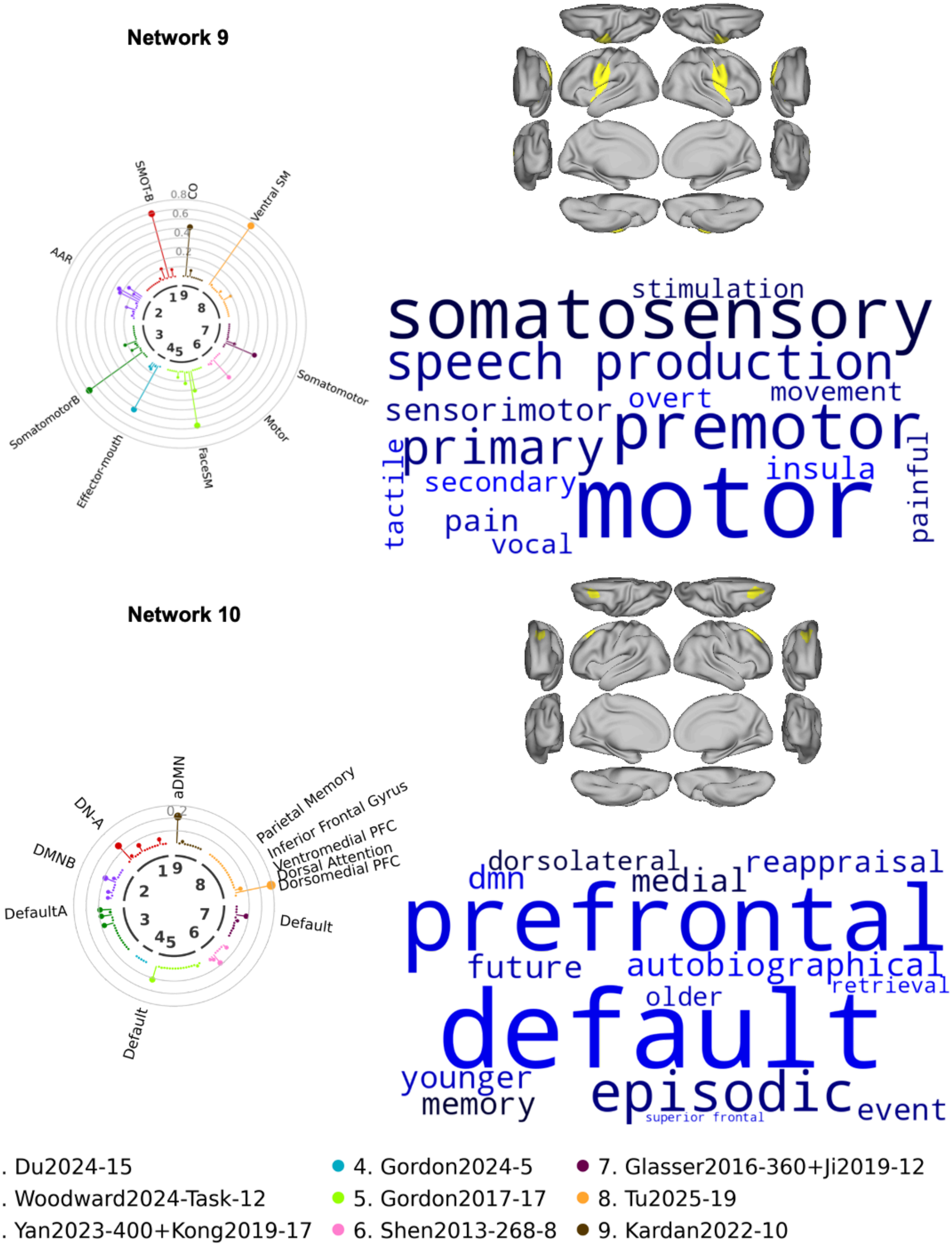

Supplementary Figure 12. *Correspondence between the 23 networks and previously reported functional networks/decoded terms (Networks 9-10)*. Left: Circular bar chart plot from the network correspondence toolbox for spatial correspondence with the network locations with

>0.35 probability and 7 atlases from the literature. Circles illustrate the magnitude of the dice coefficient in intervals of 0.1. Only significant networks from the spatial permutation test ($p<0.05$) were named. Top right: the network locations with >0.35 probability visualized on a template brain surface. Top bottom: terms with highly correlated meta-analytic activation maps to the network topography.

Network 11

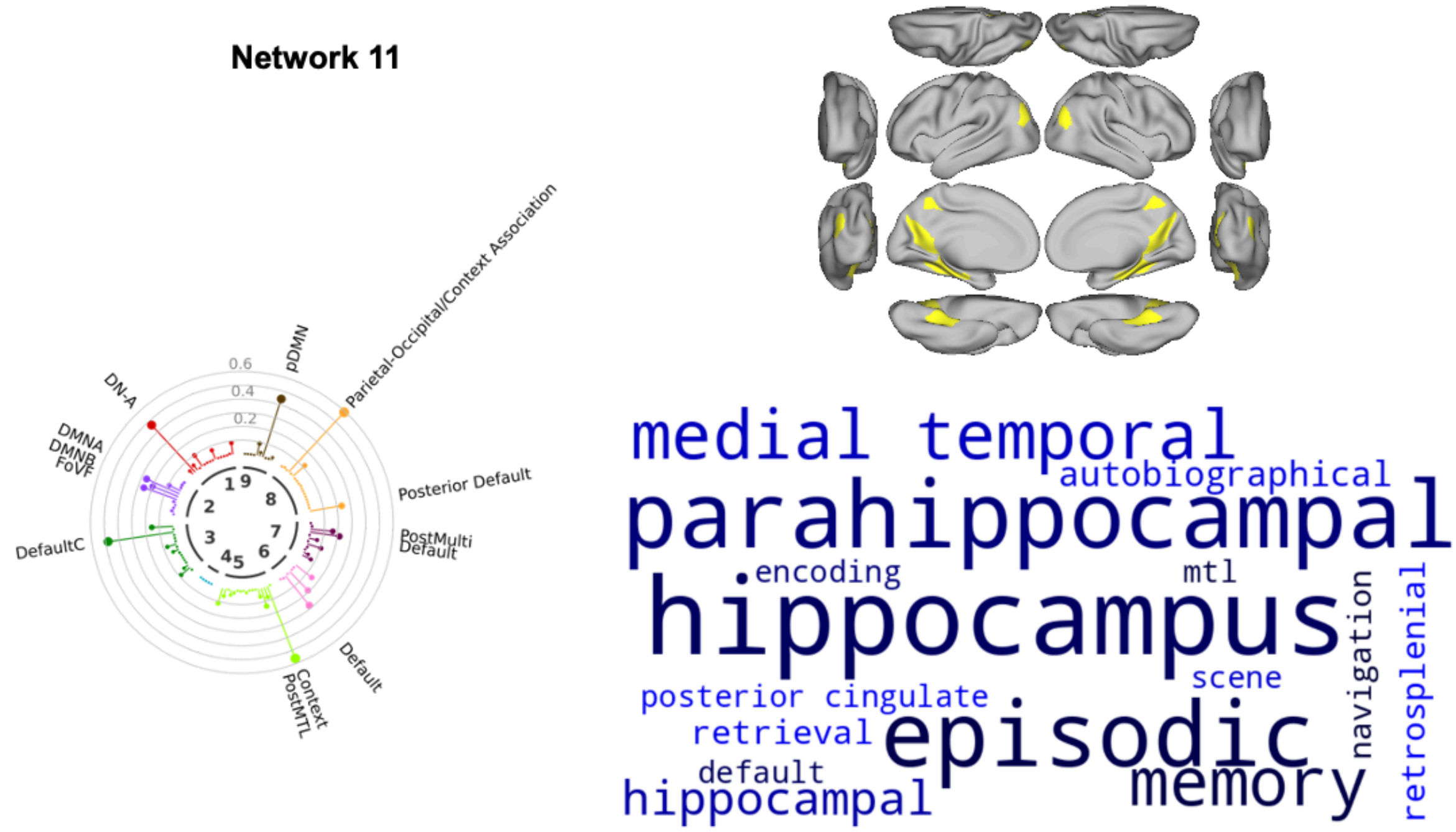

Network 12

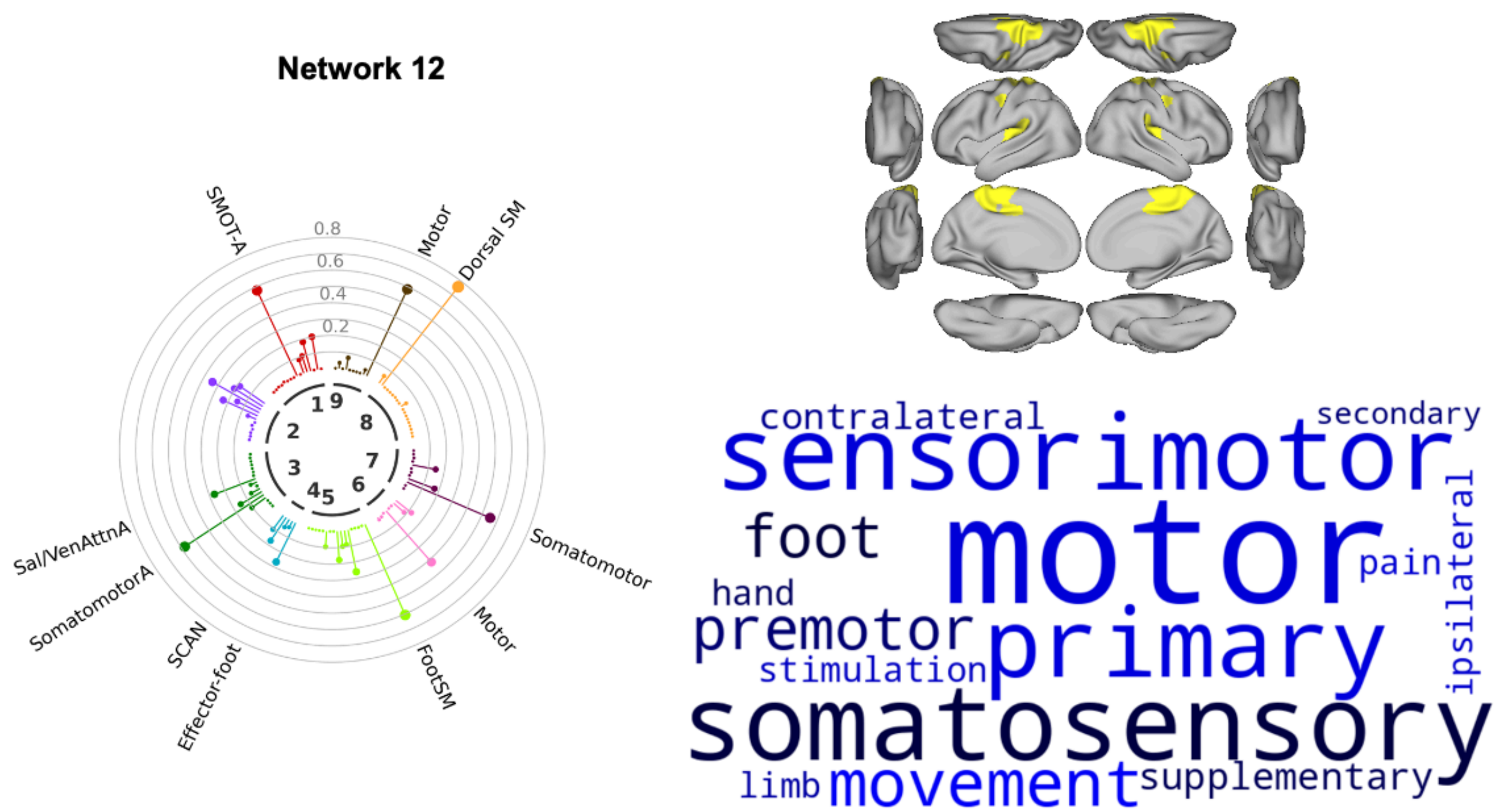

- 1. Du2024-15
- 2. Woodward2024-Task-12
- 3. Yan2023-400+Kong2019-17
- 4. Gordon2024-5
- 5. Gordon2017-17
- 6. Shen2013-268-8
- 7. Glasser2016-360+Ji2019-12
- 8. Tu2025-19
- 9. Kardan2022-10

Supplementary Figure 13. *Correspondence between the 23 networks and previously reported functional networks/decoded terms (Networks 11-12)*. Left: Circular bar chart plot from the network correspondence toolbox for spatial correspondence with the network locations with >0.35 probability and 7 atlases from the literature. Circles illustrate the magnitude of the dice coefficient in intervals of 0.1. Only significant networks from the spatial permutation test ($p<0.05$) were named. Top right: the network locations with >0.35 probability visualized on a template brain surface. Top bottom: terms with highly correlated meta-analytic activation maps to the network topography.

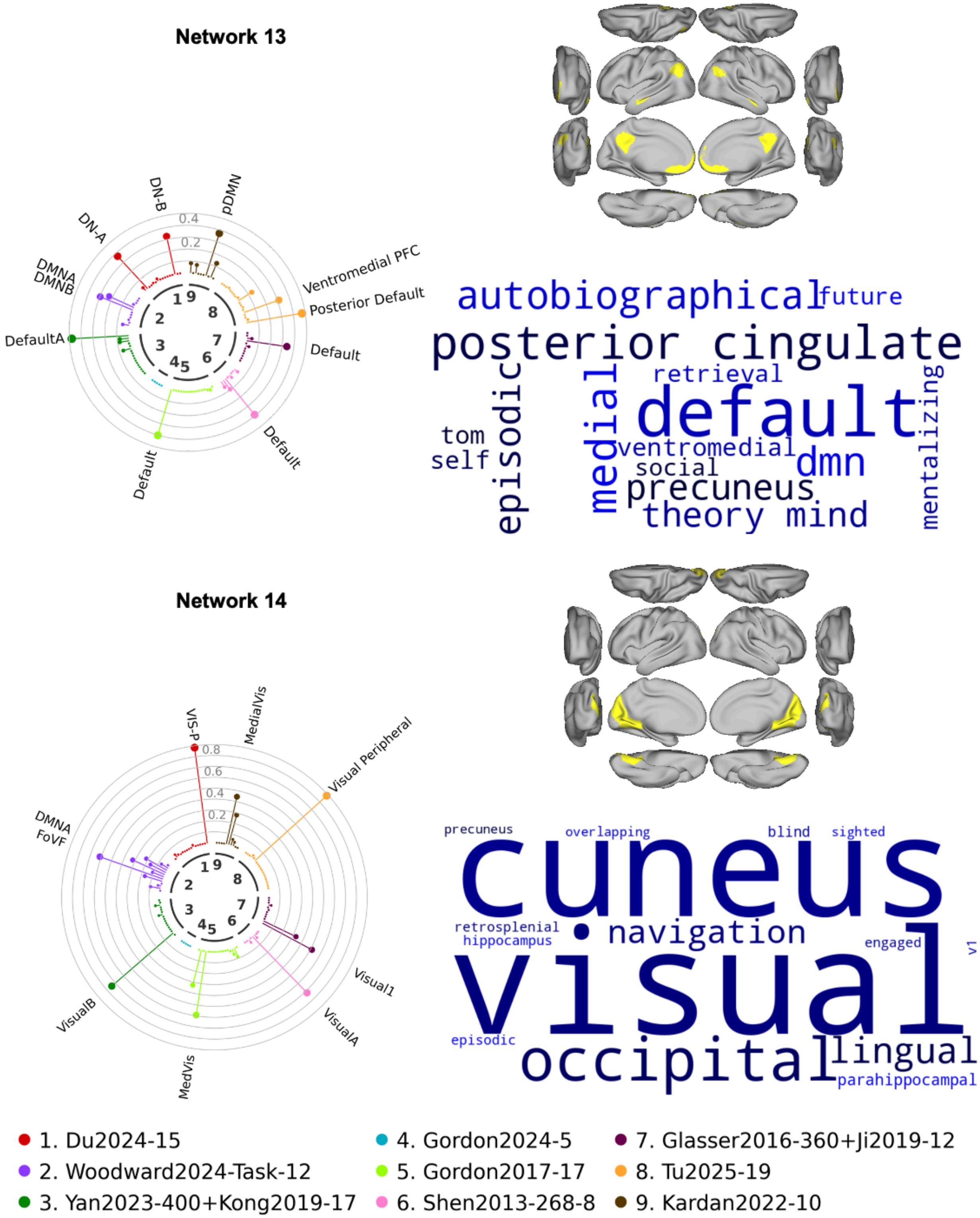

Network 13
0.4
0.2
DN-B
pDMN
DN-A
DMNA
DMNB
Ventromedial PFC
Posterior Default
DefaultA
Default
Default
Default
1
2
3
4
5
6
7
8
9
autobiographical
future
posterior cingulate
retrieval
episodic
medial
default
mentalizing
tom
self
ventromedial
social
dmn
precuneus
theory mind
Network 14
0.8
0.6
0.4
0.2
VIS-P
MedialVis
Visual Peripheral
DMNA
FoVF
Visual1
VisualA
VisualB
MedVis
precuneus
overlapping
blind
sighted
cuneus
retrosplenial
hippocampus
navigation
engaged
v1
visual
episodic
occipital
lingual
parahippocampal
1. Du2024-15
2. Woodward2024-Task-12
3. Yan2023-400+Kong2019-17
4. Gordon2024-5
5. Gordon2017-17
6. Shen2013-268-8
7. Glasser2016-360+Ji2019-12
8. Tu2025-19
9. Kardan2022-10

Supplementary Figure 14. *Correspondence between the 23 networks and previously reported functional networks/decoded terms (Networks 13-14)*. Left: Circular bar chart plot from the network correspondence toolbox for spatial correspondence with the network locations with >0.35 probability and 7 atlases from the literature. Circles illustrate the magnitude of the dice coefficient in intervals of 0.1. Only significant networks from the spatial permutation test ($p<0.05$) were named. Top right: the network locations with >0.35 probability visualized on a template brain surface. Top bottom: terms with highly correlated meta-analytic activation maps to the network topography.

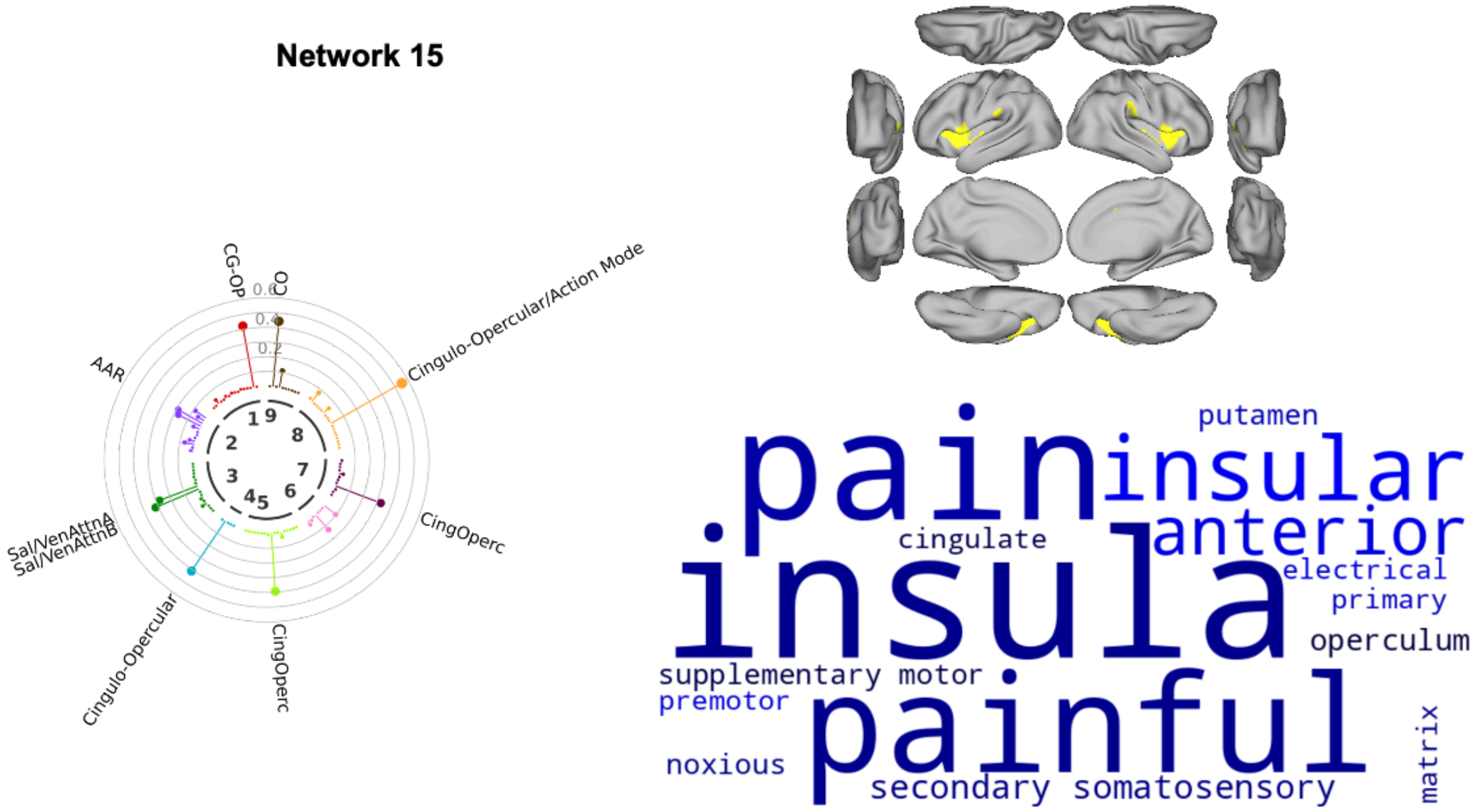
Network 15
CG-OP
CO
Cingulo-Opercular/Action Mode
AAR
Sal/VenAttnA
Sal/VenAttnB
CingOperc
Cingulo-Opercular
CingOperc
0.6
0.4
0.2
pain
insular
putamen
anterior
cingulate
insula
electrical
primary
operculum
supplementary motor
premotor
painful
noxious
secondary somatosensory
matrix

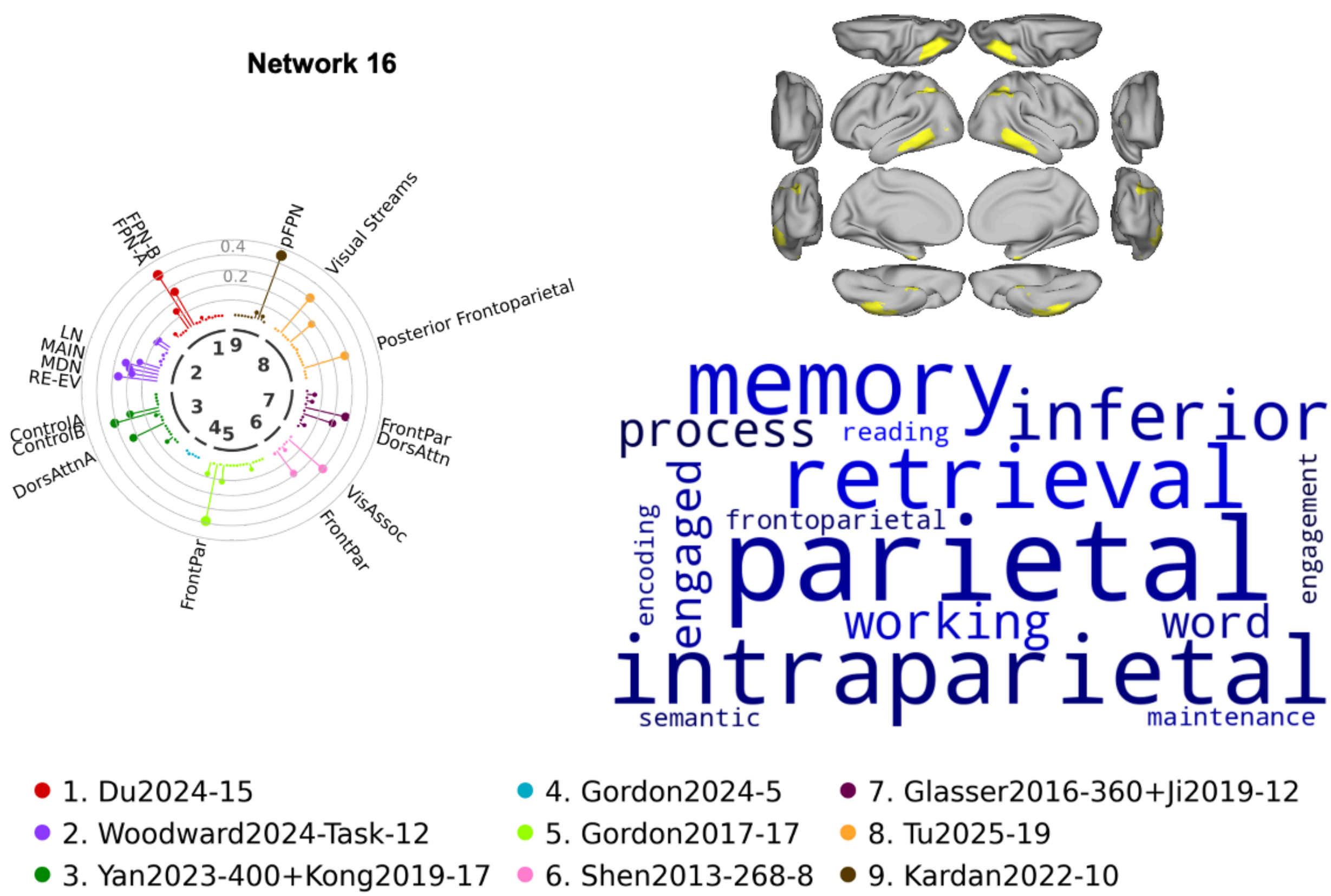
Network 16
FPN-B
FPN-A
pFPN
Visual Streams
Posterior Frontoparietal
LN
MAIN
MDN
RE-EV
ControlA
ControlB
DorsAttnA
FrontPar
DorsAttn
VisAssoc
FrontPar
FrontPar
0.4
0.2
memory
process
reading
inferior
retrieval
engaged
encoding
frontoparietal
parietal
engagement
working
word
intraparietal
semantic
maintenance
1. Du2024-15
2. Woodward2024-Task-12
3. Yan2023-400+Kong2019-17
4. Gordon2024-5
5. Gordon2017-17
6. Shen2013-268-8
7. Glasser2016-360+Ji2019-12
8. Tu2025-19
9. Kardan2022-10

Supplementary Figure 15. *Correspondence between the 23 networks and previously reported functional networks/decoded terms (Networks 15-16)*. Left: Circular bar chart plot from the network correspondence toolbox for spatial correspondence with the network locations with >0.35 probability and 7 atlases from the literature. Circles illustrate the magnitude of the dice coefficient in intervals of 0.1. Only significant networks from the spatial permutation test ($p<0.05$) were named. Top right: the network locations with >0.35 probability visualized on a template brain surface. Top bottom: terms with highly correlated meta-analytic activation maps to the network topography.

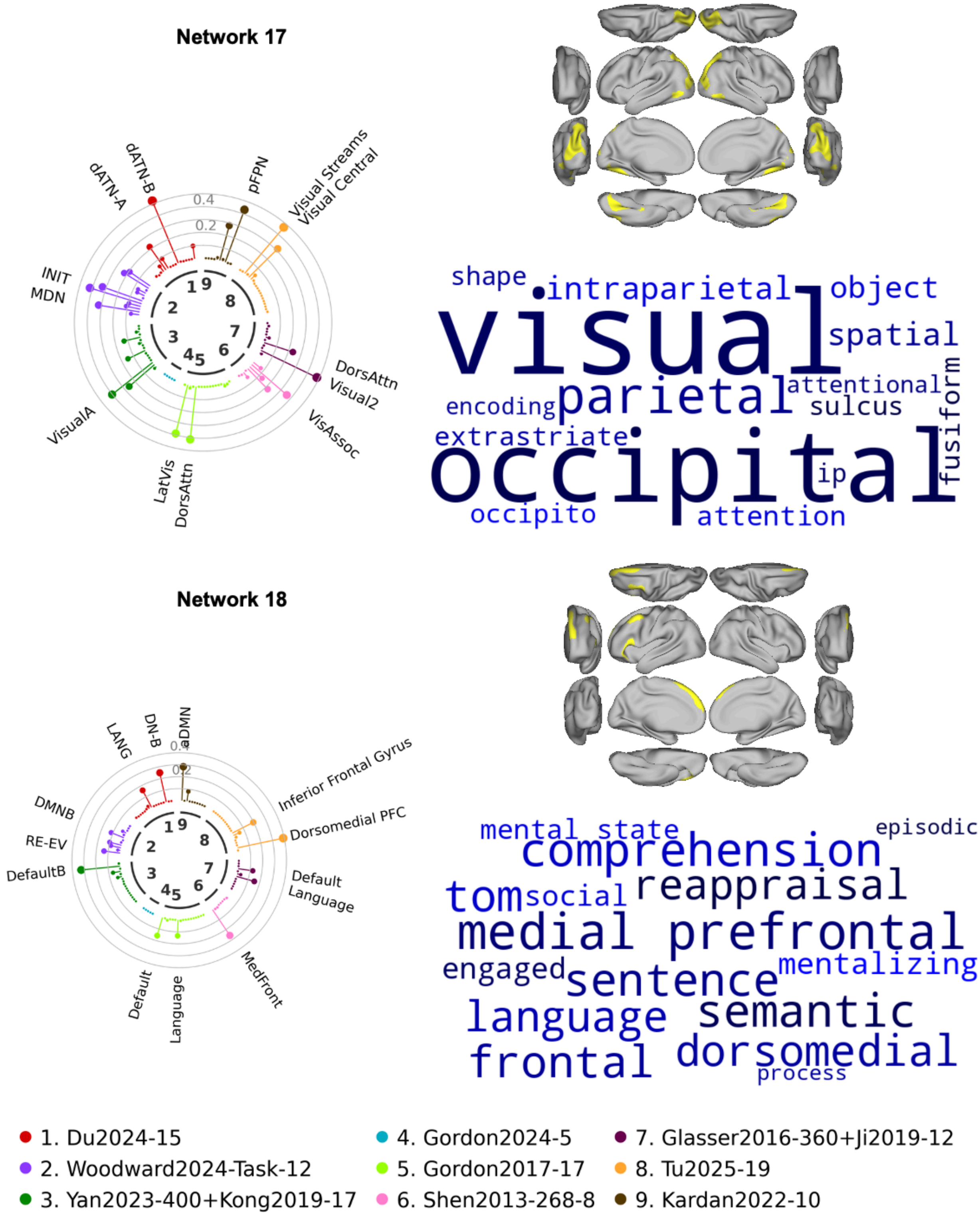
Network 17
dATN-A
dATN-B
pFPN
Visual Streams
Visual Central
INIT
MDN
DorsAttn
Visual2
VisualA
VisAssoc
LatVis
DorsAttn
0.4
0.2
1 9
2
8
3
7
6
4 5
shape
intraparietal
object
visual
spatial
parietal
attentional
fusiform
encoding
sulcus
extrastriate
occipital
ip
occipito
attention
Network 18
LANG
DN-B
aDMN
Inferior Frontal Gyrus
DMNB
Dorsomedial PFC
RE-EV
DefaultB
Default
Language
MedFront
Default
Language
mental state
episodic
comprehension
tom
social
reappraisal
medial prefrontal
engaged
sentence
mentalizing
language
semantic
frontal
dorsomedial
process
1. Du2024-15
2. Woodward2024-Task-12
3. Yan2023-400+Kong2019-17
4. Gordon2024-5
5. Gordon2017-17
6. Shen2013-268-8
7. Glasser2016-360+Ji2019-12
8. Tu2025-19
9. Kardan2022-10

Supplementary Figure 16. *Correspondence between the 23 networks and previously reported functional networks/decoded terms (Networks 17-18)*. Left: Circular bar chart plot from the network correspondence toolbox for spatial correspondence with the network locations with >0.35 probability and 7 atlases from the literature. Circles illustrate the magnitude of the dice coefficient in intervals of 0.1. Only significant networks from the spatial permutation test ($p<0.05$) were named. Top right: the network locations with >0.35 probability visualized on a template brain surface. Top bottom: terms with highly correlated meta-analytic activation maps to the network topography.

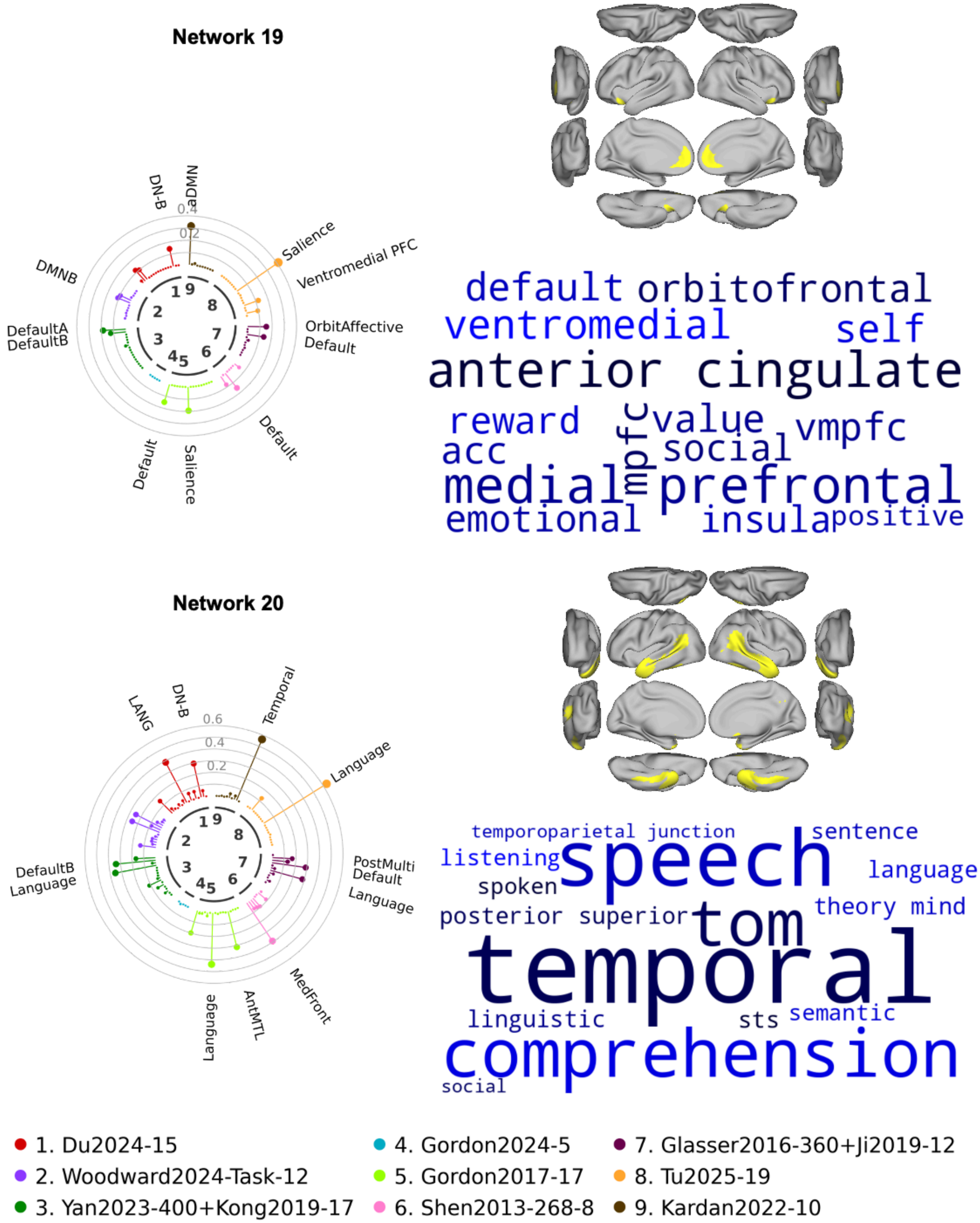
Network 19
aDMN
DN-B
0.4
0.2
Salience
Ventromedial PFC
DMNB
DefaultA
DefaultB
OrbitAffective
Default
Default
Default
Salience
1 9
2
8
3
7
4 5
6
default orbitofrontal
ventromedial self
anterior cingulate
reward value vmpfc
acc social
mpfc
medial prefrontal
emotional insula positive
Network 20
Temporal
LANG
DN-B
0.6
0.4
0.2
Language
DefaultB
Language
PostMulti
Default
Language
MedFront
AntMTL
Language
temporoparietal junction
sentence
listening
speech
language
spoken
posterior superior
tom
theory mind
temporal
linguistic
sts
semantic
comprehension
social
1. Du2024-15
2. Woodward2024-Task-12
3. Yan2023-400+Kong2019-17
4. Gordon2024-5
5. Gordon2017-17
6. Shen2013-268-8
7. Glasser2016-360+Ji2019-12
8. Tu2025-19
9. Kardan2022-10

Supplementary Figure 17. *Correspondence between the 23 networks and previously reported functional networks/decoded terms (Networks 19-20)*. Left: Circular bar chart plot from the network correspondence toolbox for spatial correspondence with the network locations with >0.35 probability and 7 atlases from the literature. Circles illustrate the magnitude of the dice coefficient in intervals of 0.1. Only significant networks from the spatial permutation test ($p<0.05$) were named. Top right: the network locations with >0.35 probability visualized on a template brain surface. Top bottom: terms with highly correlated meta-analytic activation maps to the network topography.

Network 21

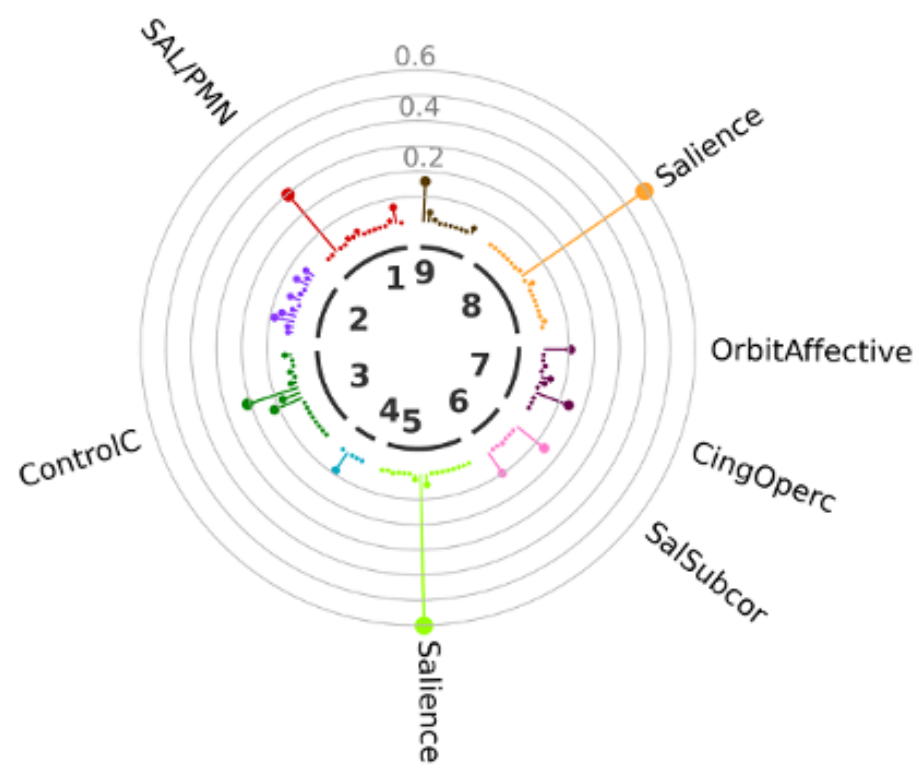

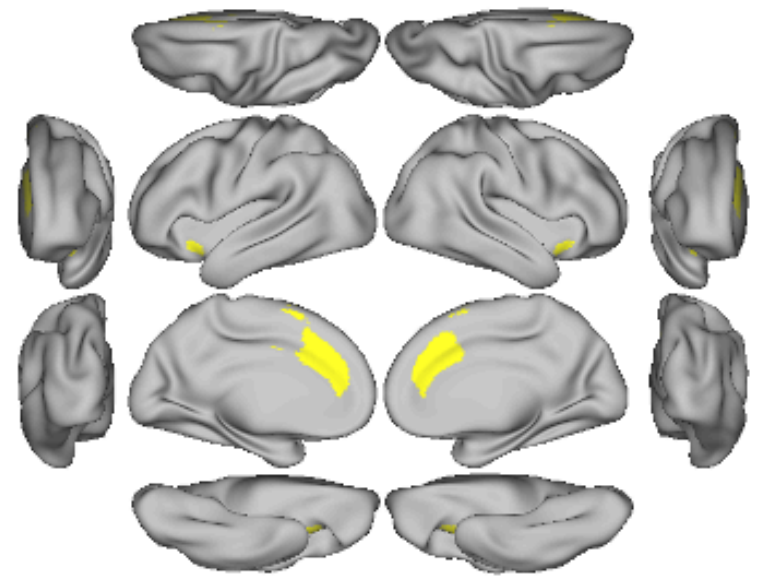

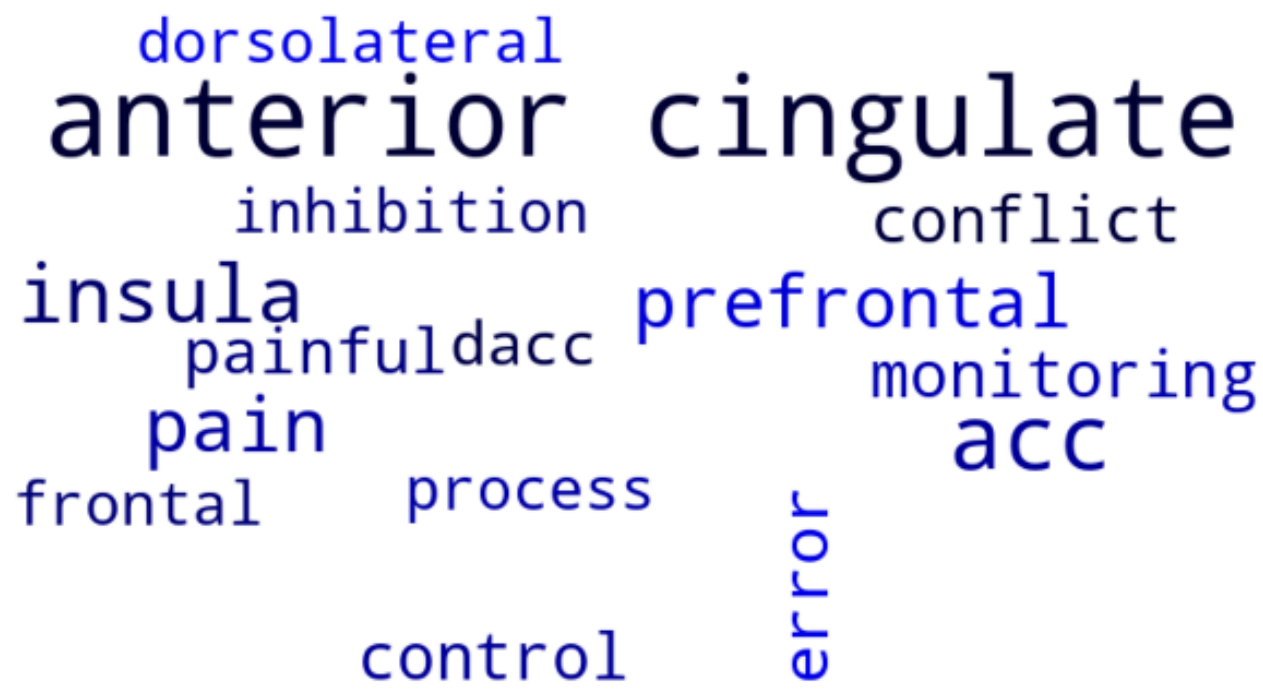

Network 22

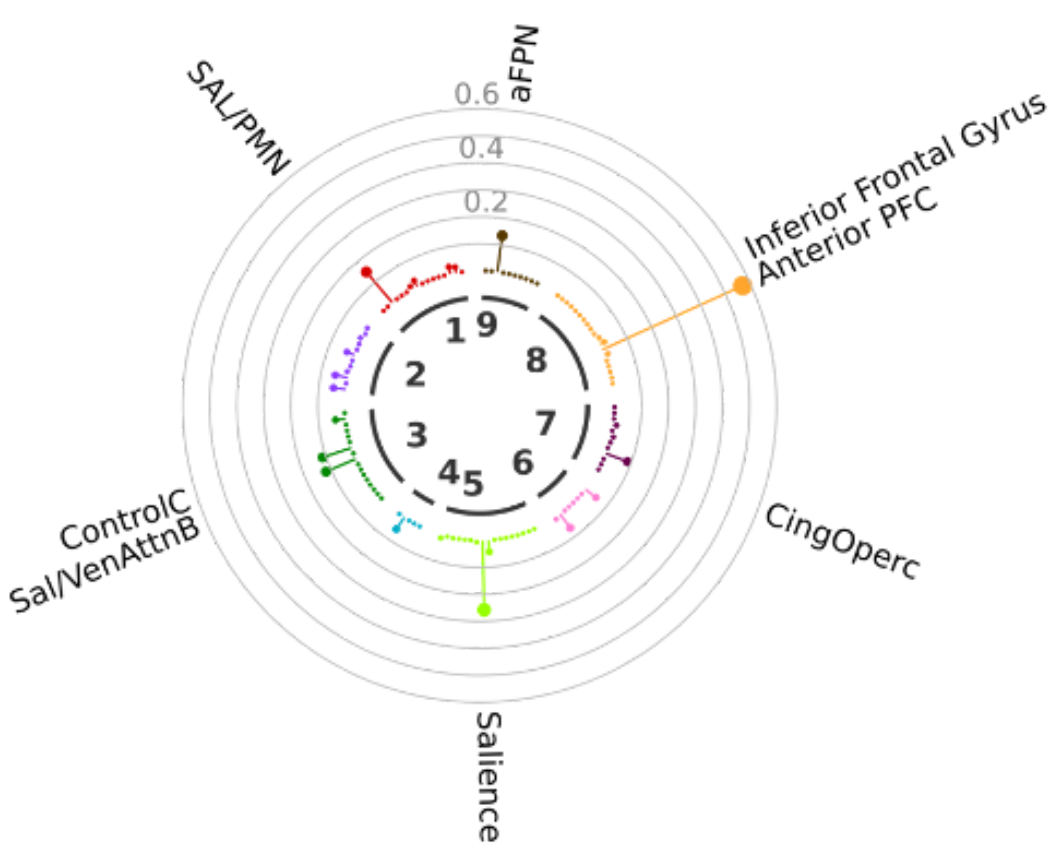

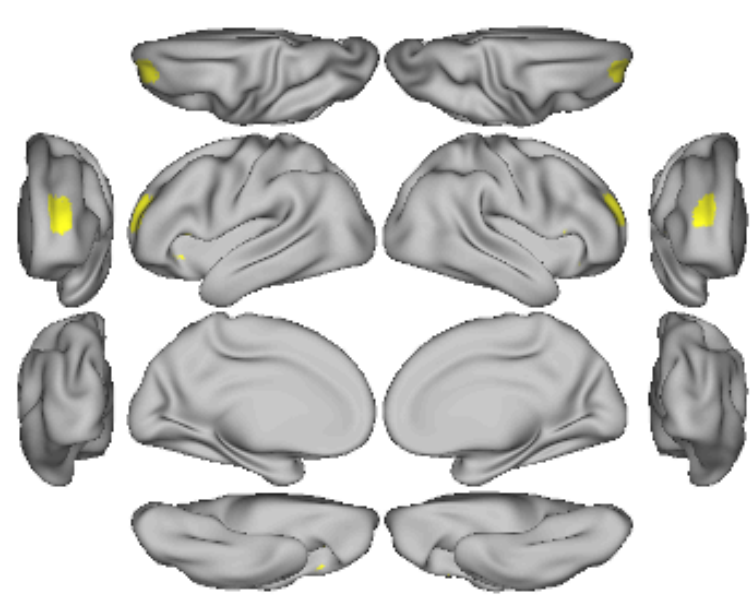

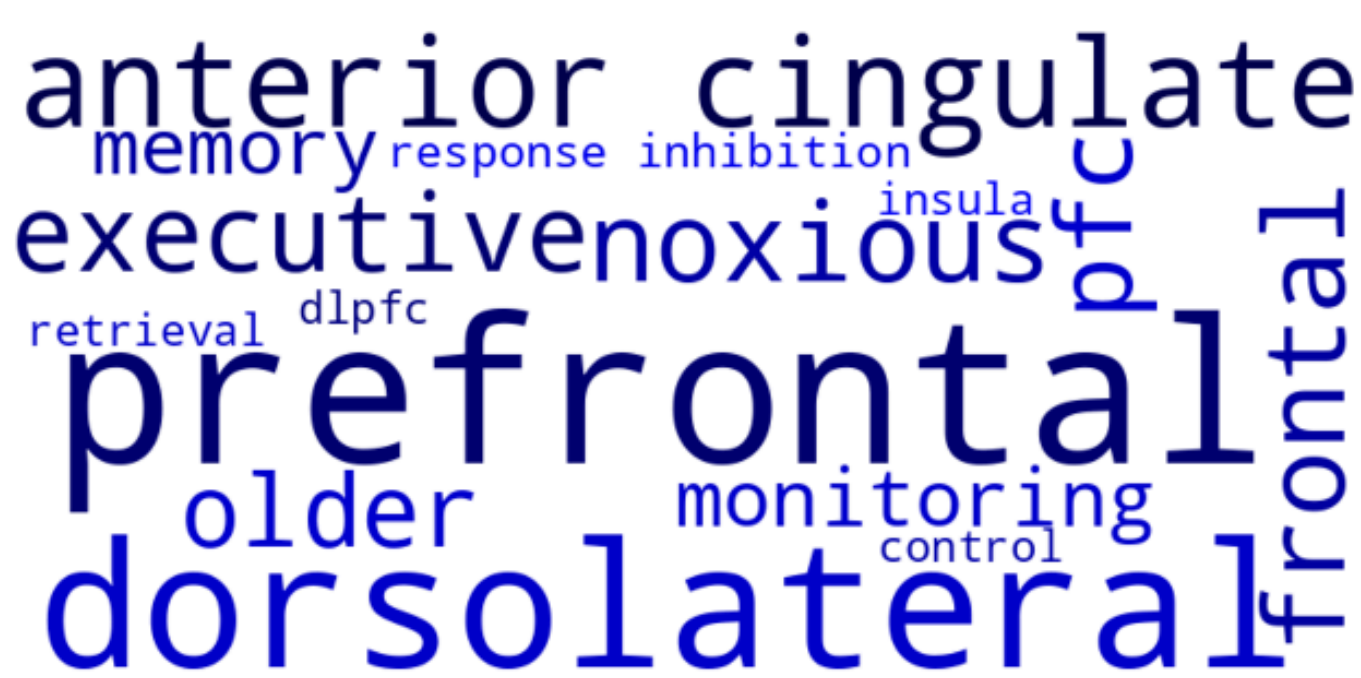

- 1. Du2024-15
- 2. Woodward2024-Task-12
- 3. Yan2023-400+Kong2019-17
- 4. Gordon2024-5
- 5. Gordon2017-17
- 6. Shen2013-268-8
- 7. Glasser2016-360+Ji2019-12
- 8. Tu2025-19
- 9. Kardan2022-10

Supplementary Figure 18. *Correspondence between the 23 networks and previously reported functional networks/decoded terms (Networks 21-22)*. Left: Circular bar chart plot from the network correspondence toolbox for spatial correspondence with the network locations with >0.35 probability and 7 atlases from the literature. Circles illustrate the magnitude of the dice coefficient in intervals of 0.1. Only significant networks from the spatial permutation test (p<0.05) were named. Top right: the network locations with >0.35 probability visualized on a template brain surface. Top bottom: terms with highly correlated meta-analytic activation maps to the network topography.

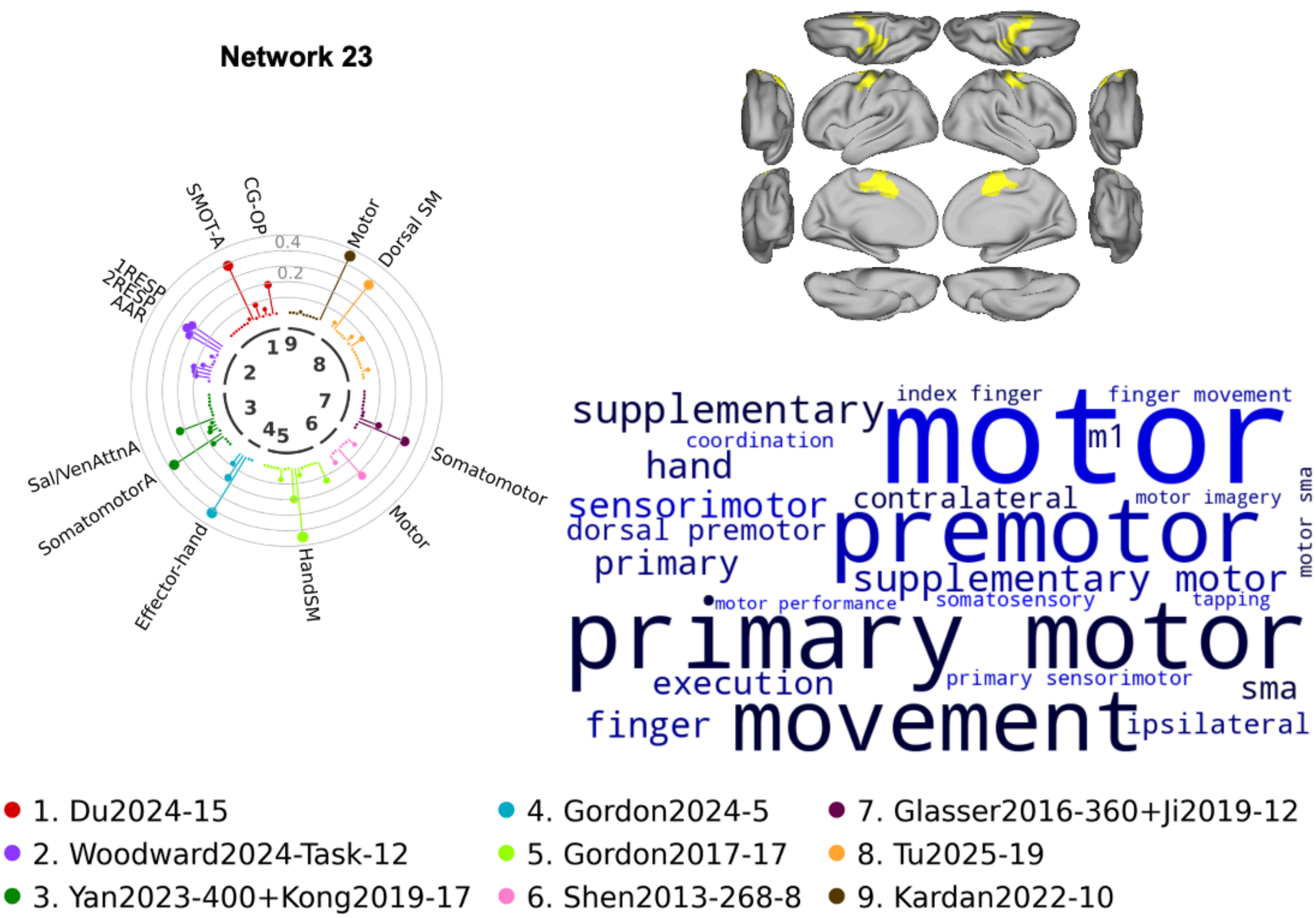

Supplementary Figure 19. *Correspondence between the 23 networks and previously reported functional networks/decoded terms (Networks 23)*. Left: Circular bar chart plot from the network correspondence toolbox for spatial correspondence with the network locations with >0.35 probability and 7 atlases from the literature. Circles illustrate the magnitude of the dice coefficient in intervals of 0.1. Only significant networks from the spatial permutation test ($p<0.05$) were named. Top right: the network locations with >0.35 probability visualized on a template brain surface. Top bottom: terms with highly correlated meta-analytic activation maps to the network topography.

23 network population-average FC (81 template sessions)

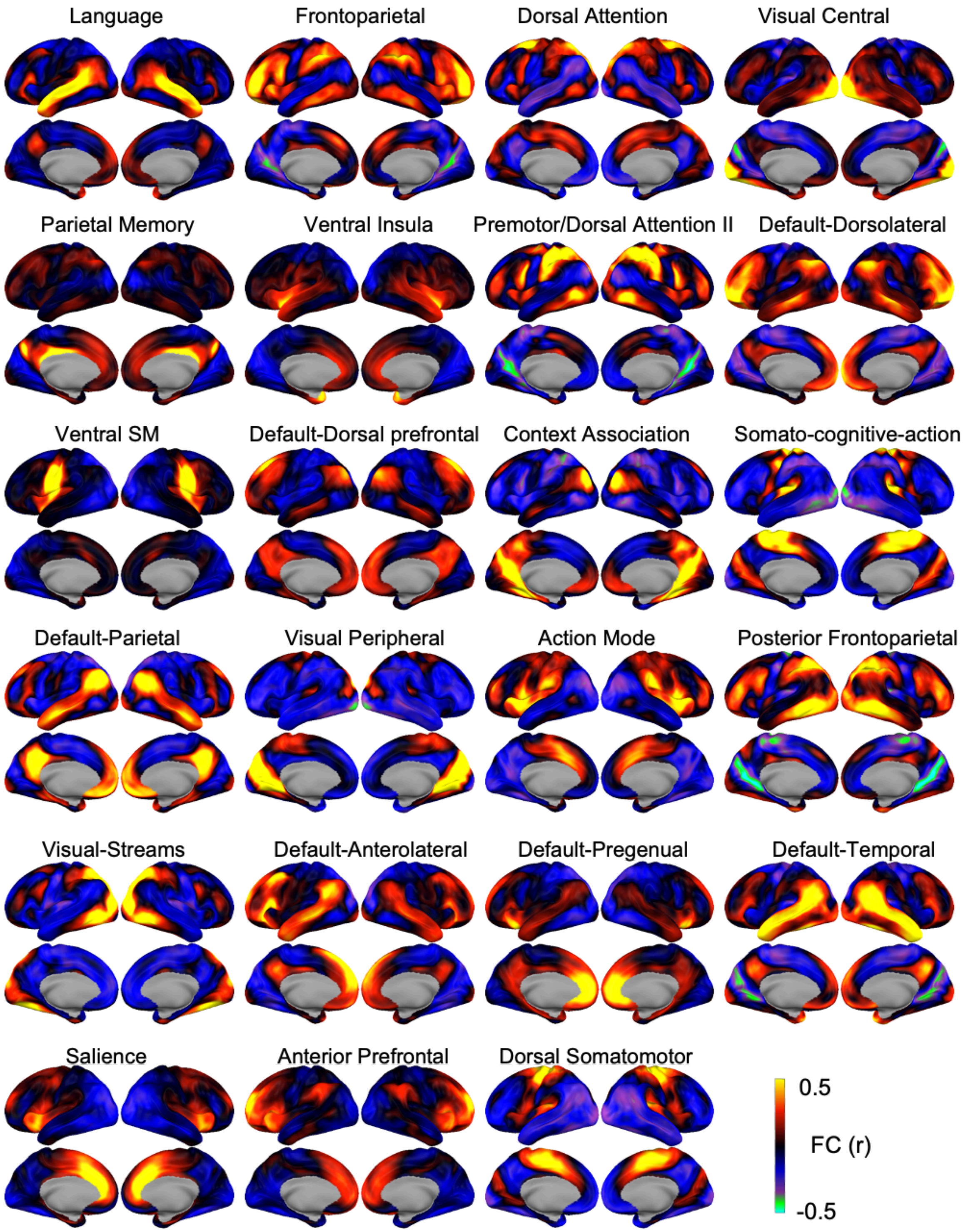

Supplementary Figure 20. *Population-average FC from the averaging of the network functional connectivity in each subject.*

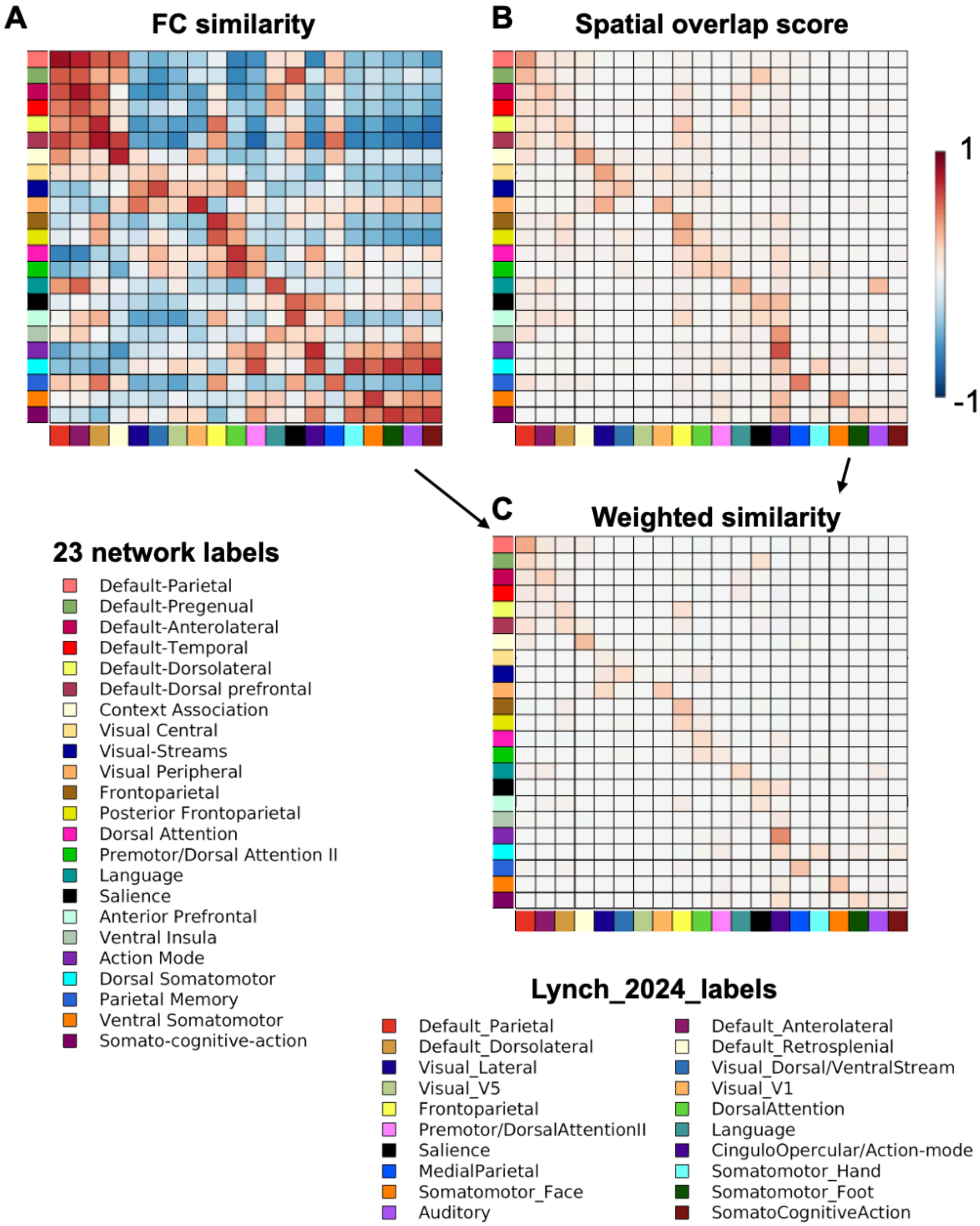

Supplementary Figure 21. *The 23 networks in the current study (rows) was matched to the 20 networks from Lynch 2024 (columns) with weighted similarity.* A) The functional connectivity (FC) similarity. B) The spatial overlap score. C) Weighted similarity.

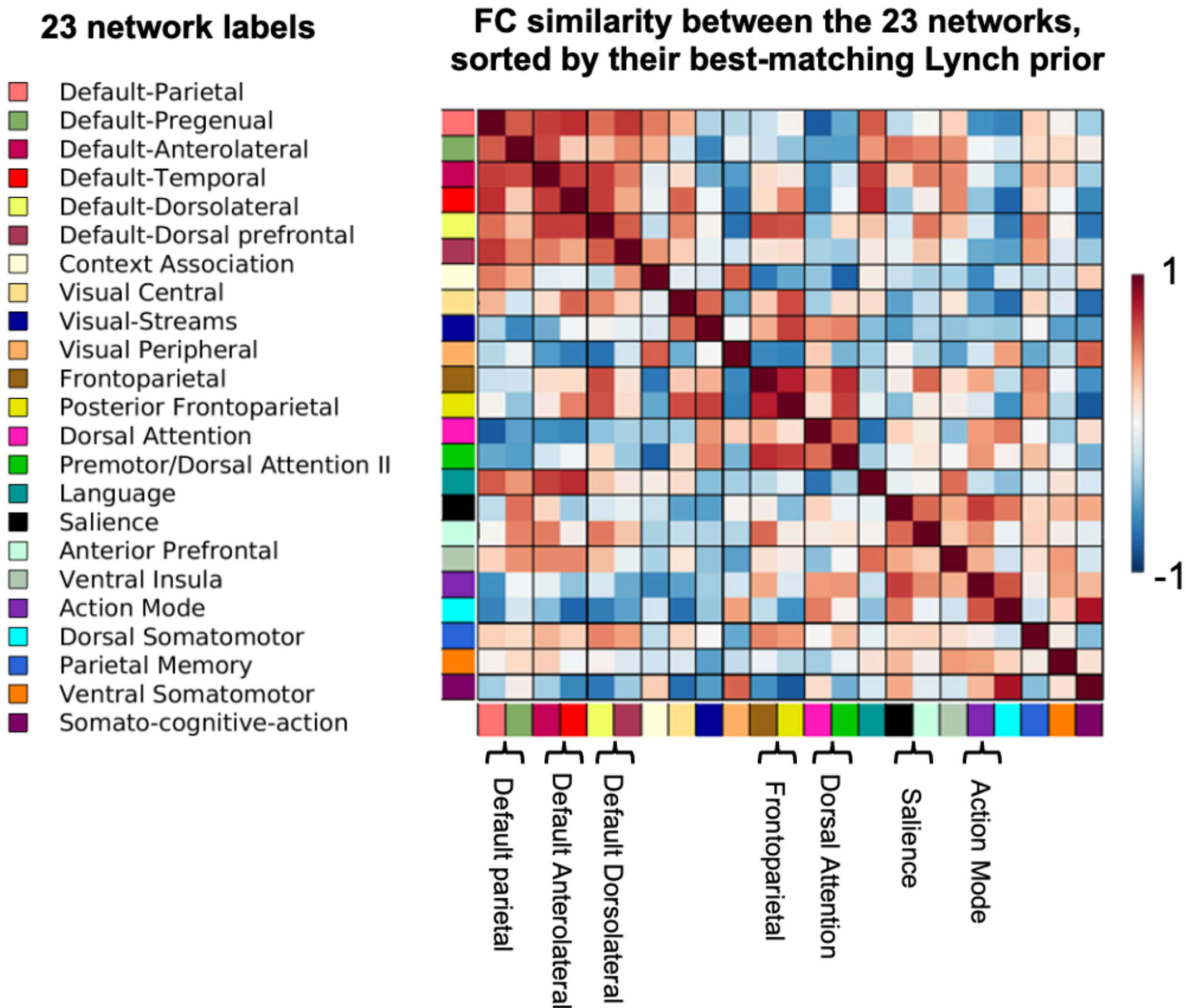

Supplementary Figure 22. *FC similarity between the 23 networks, sorted by their best-matching Lynch prior. The blocks show that some of the 23 networks match to the same network in Lynch 2024.*

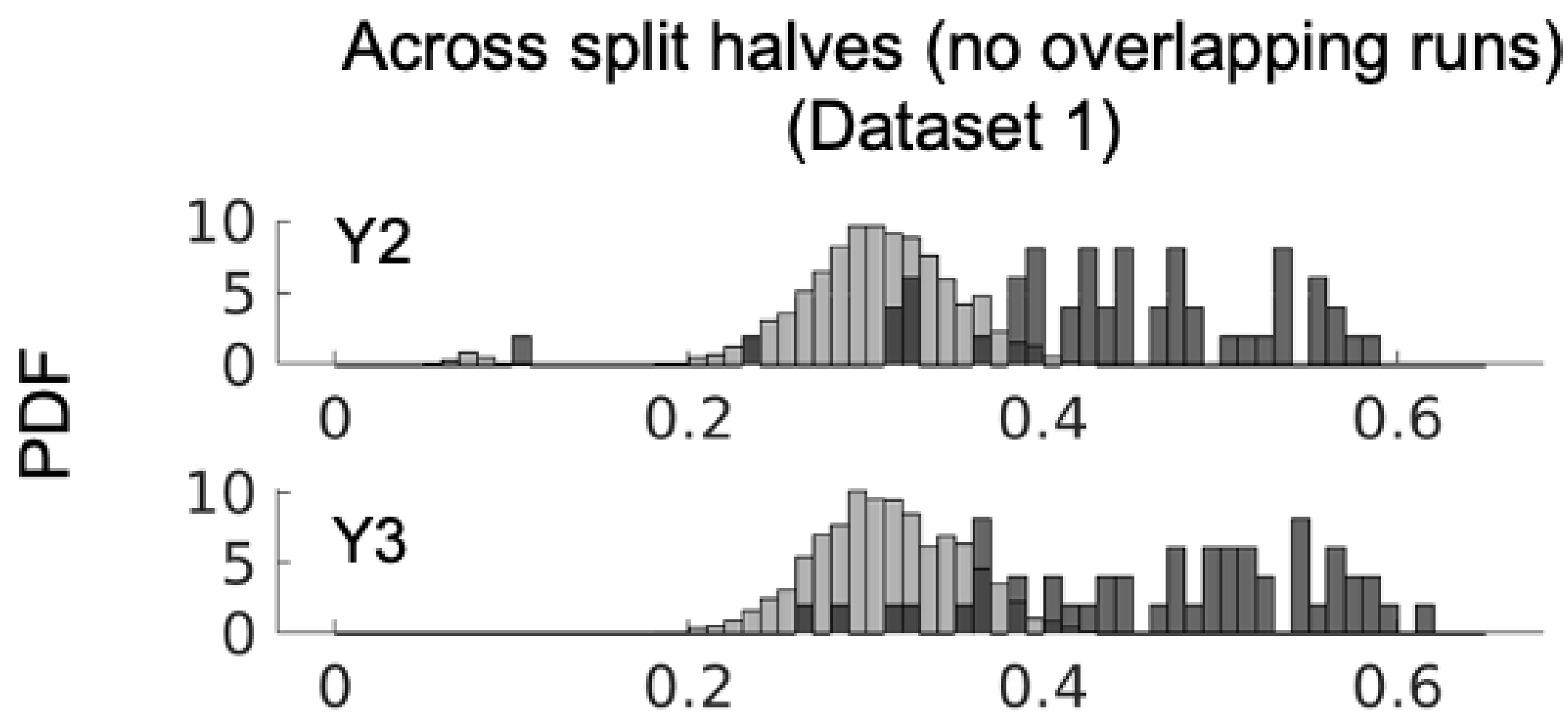

Supplementary Figure 23. Within versus between individual NMI across split halves (Dataset 1) using split halves with no overlaps in runs.

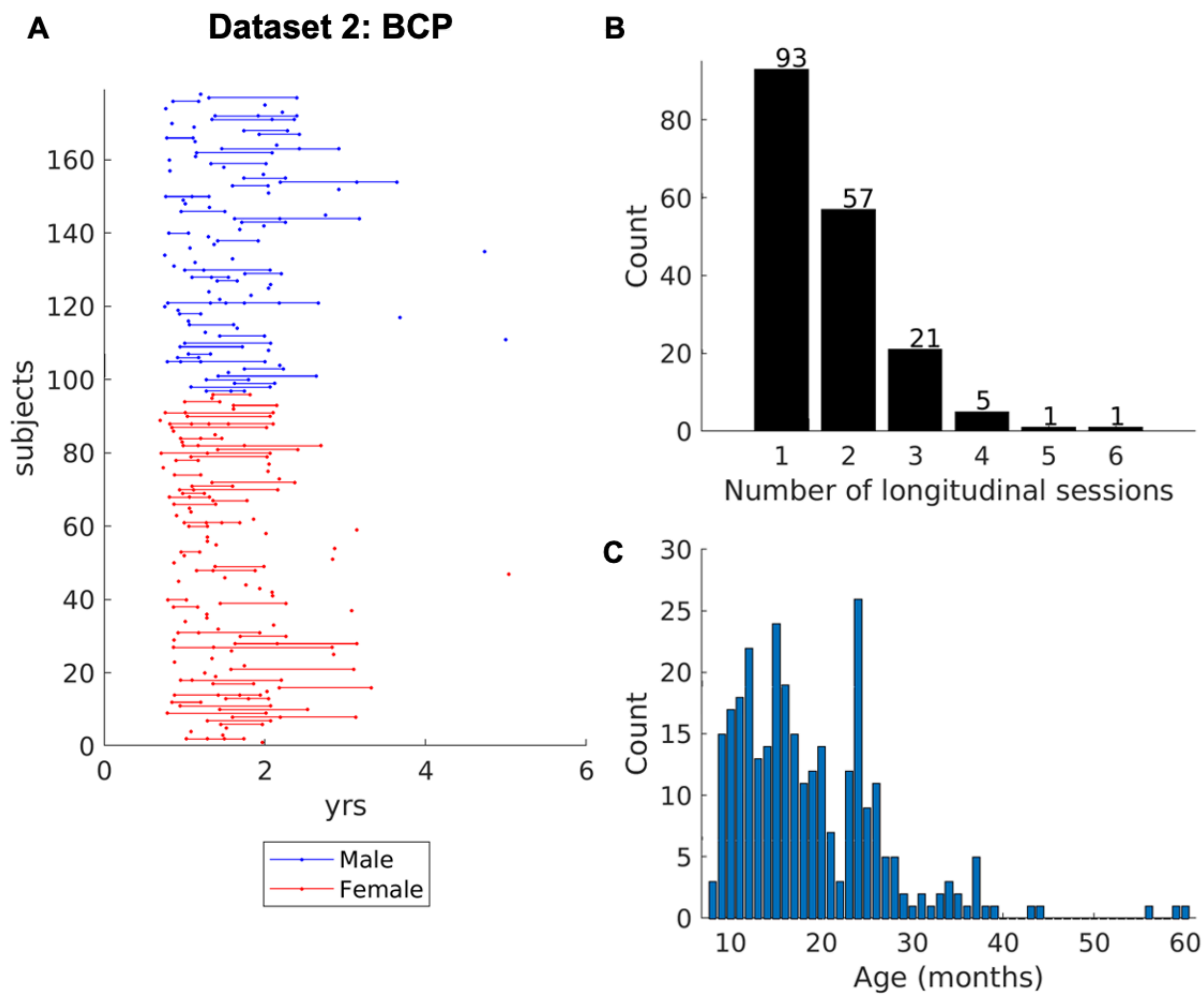

Supplementary Figure 24. *Dataset demographics (BCP).* A) The age at fMRI session for each participant, with participants sorted by Male (blue) and Female (red). B) Number of participants with 1-6 longitudinal sessions. C) Number of sessions each month.

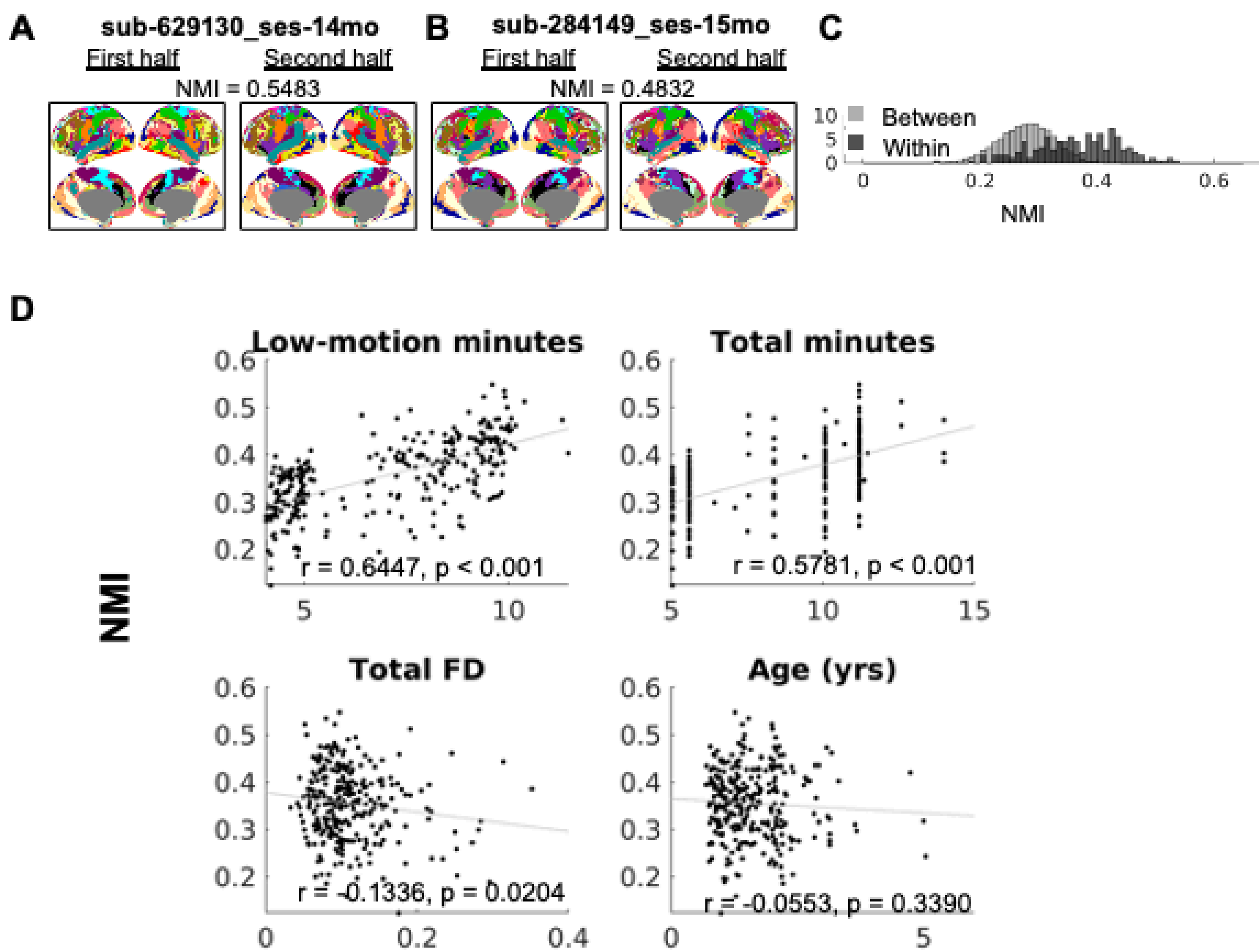

Supplementary Figure 25. *Individual-specific network topography for 23 networks (Dataset 2).* A-B) Individual-specific functional networks using fMRI data from two non-overlapping splits of two example participants. C) Within-session NMI compared to between-session NMI for 178 sessions from 178 unique participants. The session with the largest amount of usable, low-motion data was chosen for each individual when multiple longitudinal sessions exist for a participant. D) The relationship between NMI and the amount of low-motion data, total scan time, total framewise displacement, and age.

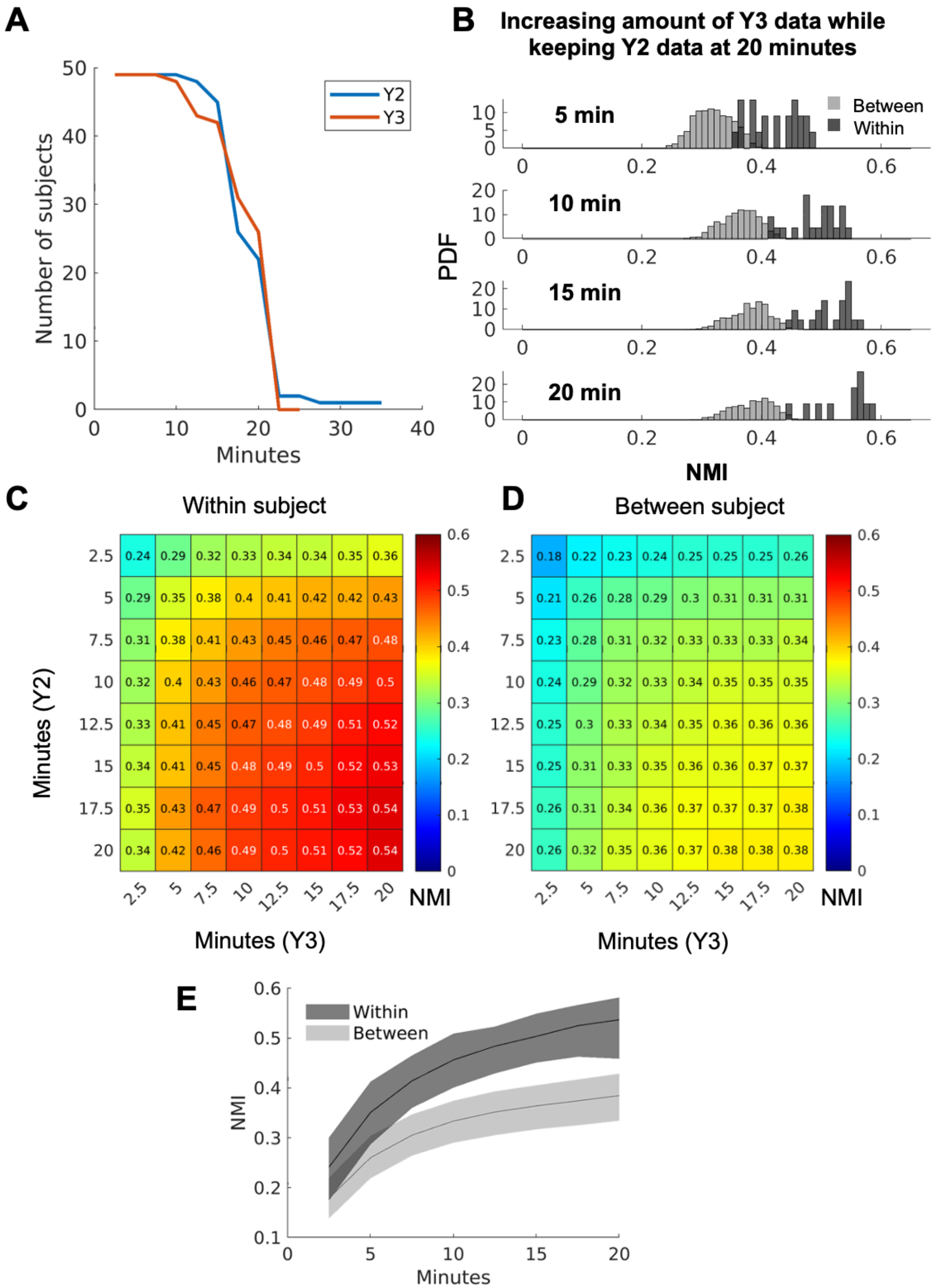
A
Number of subjects
Minutes
Y2
Y3
B
Increasing amount of Y3 data while keeping Y2 data at 20 minutes
5 min
10 min
15 min
20 min
Between
Within
PDF
NMI
C
Within subject
Minutes (Y2)
Minutes (Y3)
NMI
0.24 0.29 0.32 0.33 0.34 0.34 0.35 0.36
0.29 0.35 0.38 0.4 0.41 0.42 0.42 0.43
0.31 0.38 0.41 0.43 0.45 0.46 0.47 0.48
0.32 0.4 0.43 0.46 0.47 0.48 0.49 0.5
0.33 0.41 0.45 0.47 0.48 0.49 0.51 0.52
0.34 0.41 0.45 0.48 0.49 0.5 0.52 0.53
0.35 0.43 0.47 0.49 0.5 0.51 0.53 0.54
0.34 0.42 0.46 0.49 0.5 0.51 0.52 0.54
D
Between subject
Minutes (Y3)
NMI
0.18 0.22 0.23 0.24 0.25 0.25 0.25 0.26
0.21 0.26 0.28 0.29 0.3 0.31 0.31 0.31
0.23 0.28 0.31 0.32 0.33 0.33 0.33 0.34
0.24 0.29 0.32 0.33 0.34 0.35 0.35 0.35
0.25 0.3 0.33 0.34 0.35 0.36 0.36 0.36
0.25 0.31 0.33 0.35 0.36 0.36 0.37 0.37
0.26 0.31 0.34 0.36 0.37 0.37 0.37 0.38
0.26 0.32 0.35 0.36 0.37 0.38 0.38 0.38
E
Within
Between
NMI
Minutes

Supplementary Figure 26. *Effect of data amount on longitudinal functional network stability.* A) Number of subjects with at least X minutes of data. B) The distribution of NMI with increasing Y3 data while keeping the Y2 data the same. C) Mean within-subject NMI for different combinations of data amounts. D) Mean between-subject NMI for different combinations of data amounts. E) Mean within- and between-subject NMI when minutes in Y2 and Y3 are the same (i.e., diagonals in C and D). The shaded areas show 83.4% CI (commonly used for comparing two means, see Supplementary Note 4).

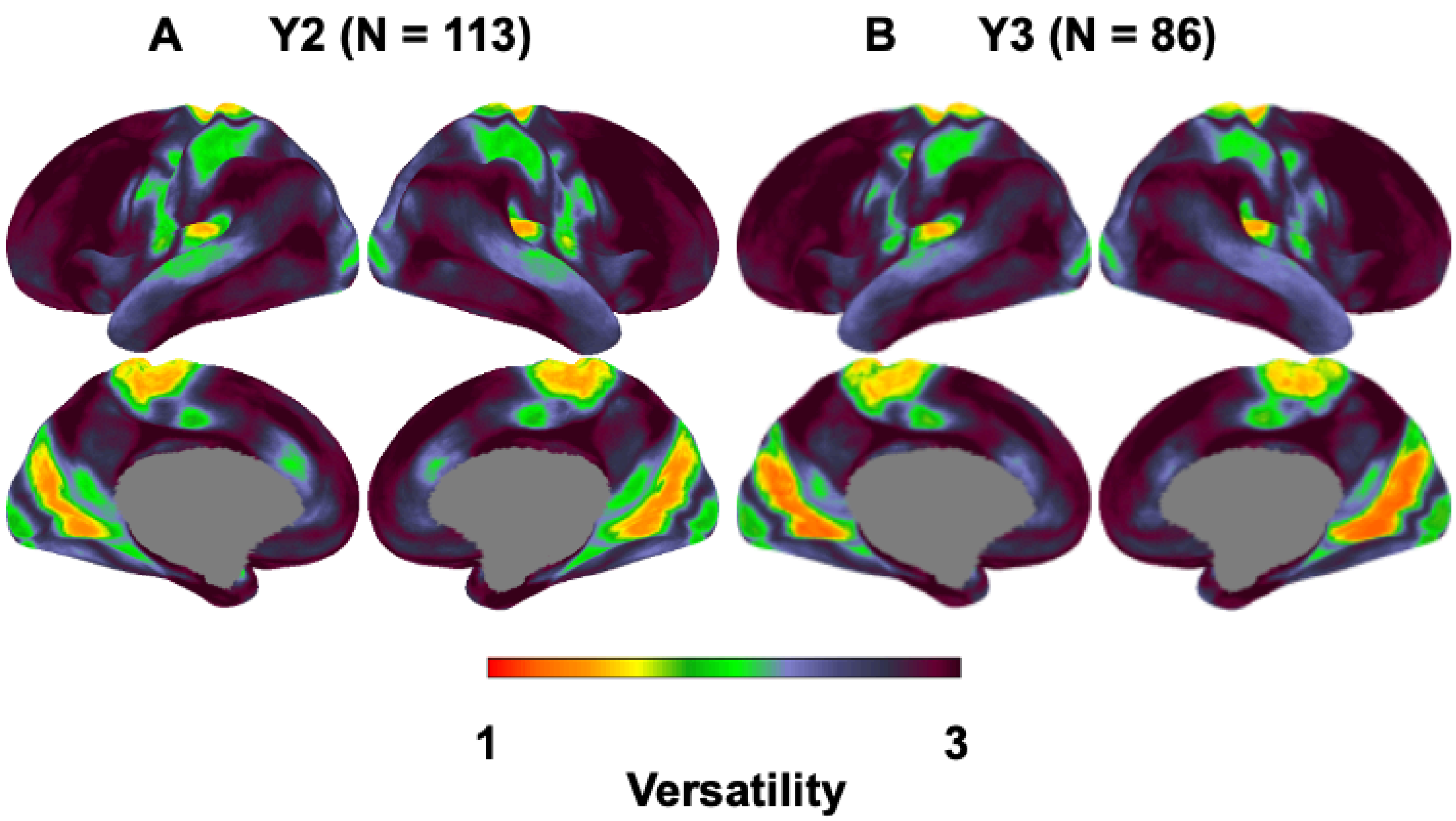

Supplementary Figure 27. *Nodal versatility across subjects in Dataset 1.* A) Y2. B) Y3.

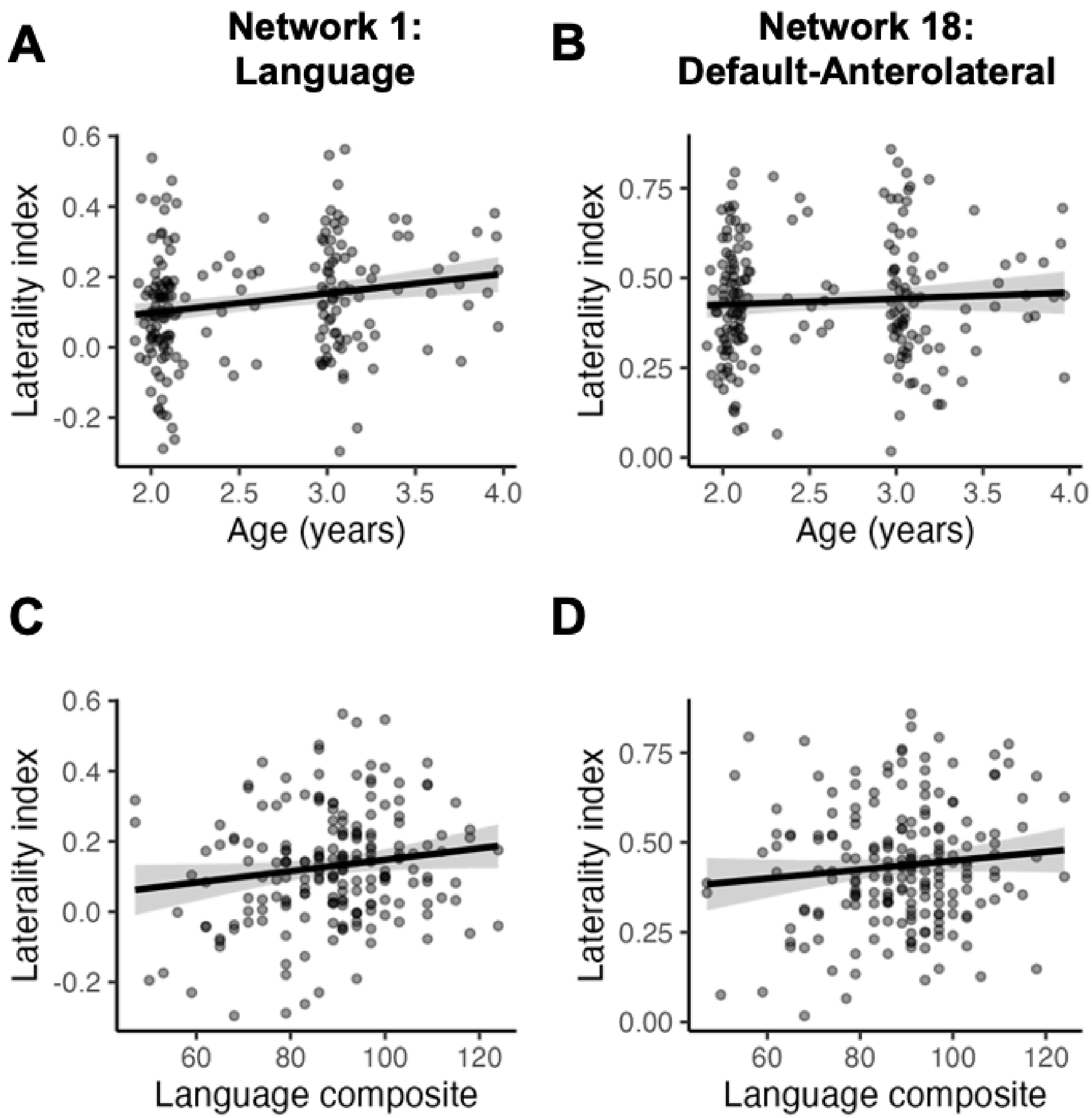

Supplementary Figure 28. *The relationship between functional network laterality and age or Bayley Scales of Infant and Toddler Development age-normalized composite scores.* A-B) Age, C-D) Language composite scores. Shaded band = 95% CI of fixed effect (age was held constant in C-D).

Supplementary References

1. Tu, J. C. *et al.* The generalizability of cortical area parcellations across early childhood. *Cerebral Cortex* **35**, bhaf116 (2025).

2. Gordon, E. M. *et al.* A somato-cognitive action network alternates with effector regions in

motor cortex. *Nature* **617**, (2023).

3. Kong, R. *et al.* A network correspondence toolbox for quantitative evaluation of novel neuroimaging results. *Nat Commun* **16**, 2930 (2025).
4. Salo, T. *et al.* NiMARE: Neuroimaging Meta-Analysis Research Environment. *Aperture Neuro* **3**, 1–32 (2023).
5. Du, J. *et al.* Organization of the human cerebral cortex estimated within individuals: networks, global topography, and function. *Journal of Neurophysiology* **131**, 1014–1082 (2024).
6. Percival, C. & Woodward, T. Cognitive modes detectable with task-based fMRI. OSF https://doi.org/10.5281/zenodo.4274397 (2025).
7. Kong, R. *et al.* Spatial Topography of Individual-Specific Cortical Networks Predicts Human Cognition, Personality, and Emotion. *Cerebral Cortex* **29**, 2533–2551 (2019).
8. Yan, X. *et al.* Homotopic local-global parcellation of the human cerebral cortex from resting-state functional connectivity. *NeuroImage* **273**, 120010 (2023).
9. Gordon, E. M. *et al.* Precision Functional Mapping of Individual Human Brains. *Neuron* **95**, (2017).
10. Shen, X., Tokoglu, F., Papademetris, X. & Constable, R. T. Groupwise whole-brain parcellation from resting-state fMRI data for network node identification. *NeuroImage* **82**, 403–415 (2013).
11. Glasser, M. F. *et al.* A multi-modal parcellation of human cerebral cortex. *Nature* **536**, 171–178 (2016).
12. Ji, J. L. *et al.* Mapping the human brain's cortical-subcortical functional network organization. *NeuroImage* **185**, 35–57 (2019).
13. Kardan, O. *et al.* Resting-state functional connectivity identifies individuals and predicts age in 8-to-26-month-olds. *Developmental Cognitive Neuroscience* **56**, 101123 (2022).
14. Yarkoni, T., Poldrack, R. A., Nichols, T. E., Van Essen, D. C. & Wager, T. D. Large-scale

automated synthesis of human functional neuroimaging data. *Nat Methods* **8**, 665–670 (2011).

15. Bird, S. & Loper, E. NLTK: The Natural Language Toolkit. in *Proceedings of the ACL Interactive Poster and Demonstration Sessions* 214–217 (Association for Computational Linguistics, Barcelona, Spain, 2004).
16. Braga, R. M., DiNicola, L. M., Becker, H. C. & Buckner, R. L. Situating the left-lateralized language network in the broader organization of multiple specialized large-scale distributed networks. *Journal of Neurophysiology* **124**, 1415–1448 (2020).
17. Lynch, C. J. *et al.* Frontostriatal salience network expansion in individuals in depression. *Nature* **633**, 624–633 (2024).
18. Lipkin, B. *et al.* Probabilistic atlas for the language network based on precision fMRI data from >800 individuals. *Sci Data* **9**, 529 (2022).
19. Zhu, Z. *et al.* Innate network mechanisms of temporal pole for semantic cognition in neonatal and adult twin studies. *Nat Commun* **16**, 3835 (2025).
20. Power, J. D. *et al.* Functional Network Organization of the Human Brain. *Neuron* **72**, 665–678 (2011).
21. Gordon, E. M. *et al.* Generation and Evaluation of a Cortical Area Parcellation from Resting-State Correlations. *Cereb. Cortex* **26**, 288–303 (2016).
22. Eggebrecht, A. T. *et al.* Joint Attention and Brain Functional Connectivity in Infants and Toddlers. *Cerebral Cortex* **27**, 1709–1720 (2017).
23. Kardan, O. *et al.* Resting-state functional connectivity identifies individuals and predicts age in 8-to-26-month-olds. *Developmental Cognitive Neuroscience* **56**, 101123 (2022).
24. Gratton, C. *et al. The Cingulo-Opercular Network Is Composed of Two Distinct Sub-Systems*. http://biorxiv.org/lookup/doi/10.1101/2022.09.16.508254 (2022) doi:10.1101/2022.09.16.508254.
25. Yeo, B. T. T. *et al.* The organization of the human cerebral cortex estimated by intrinsic

functional connectivity. *Journal of Neurophysiology* **106**, 1125–1165 (2011).

26. Gordon, E. M. *et al.* Individual-specific features of brain systems identified with resting state functional correlations. *NeuroImage* **146**, 918–939 (2017).
27. Nomi, J. S. *et al.* Dynamic functional network connectivity reveals unique and overlapping profiles of insula subdivisions. *Human Brain Mapping* **37**, 1770–1787 (2016).
28. Di Plinio, S. & Ebisch, S. J. H. Brain network profiling defines functionally specialized cortical networks. *Human Brain Mapping* **39**, 4689–4706 (2018).
29. Myoraku, A. *et al.* Age-dependent brain morphometry in Major Depressive disorder. *NeuroImage: Clinical* **33**, 102924 (2022).
30. Gordon, E. M. *et al.* Default-mode network streams for coupling to language and control systems. *Proc Natl Acad Sci U S A* **117**, 17308–17319 (2020).
31. Laumann, T. O. *et al.* Functional System and Areal Organization of a Highly Sampled Individual Human Brain. *Neuron* **87**, 657–670 (2015).
32. Alves, P. N. *et al.* An improved neuroanatomical model of the default-mode network reconciles previous neuroimaging and neuropathological findings. *Commun Biol* **2**, 370 (2019).
33. Paquola, C. *et al.* The architecture of the human default mode network explored through cytoarchitecture, wiring and signal flow. *Nat Neurosci* **28**, 654–664 (2025).
34. Badke D'Andrea, C. *et al.* Action-mode subnetworks for decision-making, action control, and feedback. *Proceedings of the National Academy of Sciences* **122**, e2502021122 (2025).
35. Dosenbach, N. U. F., Raichle, M. E. & Gordon, E. M. The brain's action-mode network. *Nat. Rev. Neurosci.* 1–11 (2025) doi:10.1038/s41583-024-00895-x.
36. Gordon, E. M. *et al.* Individualized Functional Subnetworks Connect Human Striatum and Frontal Cortex. *Cerebral Cortex* **32**, 2868–2884 (2022).
37. Palomero-Gallagher, N. *et al.* Human Pregenual Anterior Cingulate Cortex: Structural, Functional, and Connectional Heterogeneity. *Cereb Cortex* **29**, 2552–2574 (2019).

38. Hermosillo, R. J. M. *et al.* A precision functional atlas of personalized network topography and probabilities. *Nat Neurosci* **27**, 1000–1013 (2024).

39. Knol, M. J., Pestman, W. R. & Grobbee, D. E. The (mis)use of overlap of confidence intervals to assess effect modification. *Eur J Epidemiol* **26**, 253–254 (2011).

40. Payton, M. E., Greenstone, M. H. & Schenker, N. Overlapping confidence intervals or standard error intervals: What do they mean in terms of statistical significance? *Journal of Insect Science* **3**, 34 (2003).

41. Balasundaram, P. & Avulakunta, I. D. Bayley Scales Of Infant and Toddler Development. in *StatPearls* (StatPearls Publishing, Treasure Island (FL), 2025).

42. Winkler, A. M., Ridgway, G. R., Webster, M. A., Smith, S. M. & Nichols, T. E. Permutation inference for the general linear model. *Neuroimage* **92**, 381–397 (2014).
